# Open Source Brain: A Collaborative Resource for Visualizing, Analyzing, Simulating, and Developing Standardized Models of Neurons and Circuits

**DOI:** 10.1016/j.neuron.2019.05.019

**Published:** 2019-08-07

**Authors:** Padraig Gleeson, Matteo Cantarelli, Boris Marin, Adrian Quintana, Matt Earnshaw, Sadra Sadeh, Eugenio Piasini, Justas Birgiolas, Robert C. Cannon, N. Alex Cayco-Gajic, Sharon Crook, Andrew P. Davison, Salvador Dura-Bernal, András Ecker, Michael L. Hines, Giovanni Idili, Frederic Lanore, Stephen D. Larson, William W. Lytton, Amitava Majumdar, Robert A. McDougal, Subhashini Sivagnanam, Sergio Solinas, Rokas Stanislovas, Sacha J. van Albada, Werner van Geit, R. Angus Silver

**Affiliations:** 1 Department of Neuroscience, Physiology and Pharmacology, University College London, London, UK; 2 MetaCell Limited, Oxford, UK; 3 Centro de Matemática, Computação e Cognição, Universidade Federal do ABC, Santo André, Brazil; 4 Computational Neuroscience Initiative and Department of Physics and Astronomy, University of Pennsylvania, Philadelphia, PA, USA; 5 School of Life Sciences, Arizona State University, Tempe, AZ, USA; 6 Annotate Software Limited, Edinburgh, UK; 7 School of Mathematical and Statistical Sciences, Arizona State University, Tempe, AZ, USA; 8 Unité de Neuroscience, Information et Complexité, Centre National de la Recherche Scientifique, Paris, France; 9 SUNY Downstate Medical Center and Kings County Hospital, Brooklyn, NY, USA; 10 Blue Brain Project, École Polytechnique Fédérale de Lausanne (EPFL), Lausanne, Switzerland; 11 Department of Neuroscience, Yale School of Medicine, New Haven, CT, USA; 12 OpenWorm Foundation, Boston, MA, USA; 13 University of California, San Diego, San Diego, CA, USA; 14 Center for Medical Informatics, Yale University, New Haven, CT, USA; 15 Department of Biomedical Science, University of Sassari, Sassari, Italy; 16 Institute of Neuroinformatics, University of Zurich and ETH Zurich, Zurich, Switzerland; 17 Institute of Neuroscience and Medicine (INM-6), Institute for Advanced Simulation (IAS-6) and JARA-Institut Brain Structure-Function Relationships (INM-10), Jülich Research Centre, Jülich, Germany

**Keywords:** computational neuroscience, circuits, networks, neurons, modelling, standardization, collaboration, simulation, open source

## Abstract

Computational models are powerful tools for exploring the properties of complex biological systems. In neuroscience, data-driven models of neural circuits that span multiple scales are increasingly being used to understand brain function in health and disease. But their adoption and reuse has been limited by the specialist knowledge required to evaluate and use them. To address this, we have developed Open Source Brain, a platform for sharing, viewing, analyzing, and simulating standardized models from different brain regions and species. Model structure and parameters can be automatically visualized and their dynamical properties explored through browser-based simulations. Infrastructure and tools for collaborative interaction, development, and testing are also provided. We demonstrate how existing components can be reused by constructing new models of inhibition-stabilized cortical networks that match recent experimental results. These features of Open Source Brain improve the accessibility, transparency, and reproducibility of models and facilitate their reuse by the wider community.

## Highlights

• Open Source Brain: an online resource of standardized models of neurons and circuits
• Automated 3D visualization, analysis, and simulation of models through the browser
• Open source infrastructure and tools for collaborative model development and testing
• Accessible, transparent, up-to-date models from different brain regions

## Graphical Abstract

**Figure:**
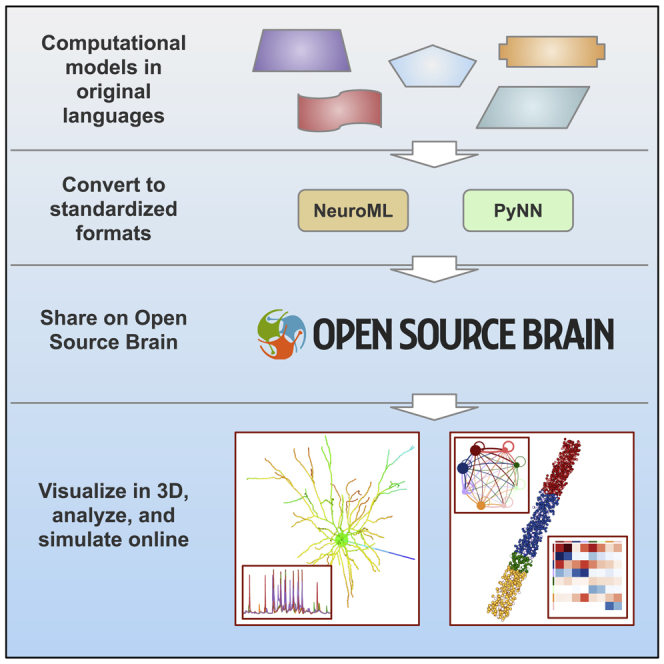

## Teaser

Open Source Brain is an online resource of neuronal and circuit models that enables browser-based visualization, analysis, and simulation. Gleeson et al. describe how the resource and tools for collaborative model development provide accessible, up-to-date models from different brain regions.

## Introduction

Computational modeling is a powerful approach for investigating and understanding information processing in neural systems (Dayan and Abbott, 2001, Herz et al., 2006, Sejnowski et al., 1988). Such models have played a central role in elucidating the mechanisms underlying synaptic transmission (Del Castillo and Katz, 1954), the action potential (Hodgkin and Huxley, 1952), dendritic integration (Rall, 1962), and, more recently, circuit function (Bezaire et al., 2016, Billings et al., 2014, Cayco-Gajic et al., 2017, Diesmann et al., 1999, Markram et al., 2015, Potjans and Diesmann, 2014, Sadeh et al., 2017).

Models range widely in their level of biological detail, ranging from reduced “top-down” models that provide insights into high-level dynamical behavior of circuits to biologically detailed “bottom-up” models (Bezaire et al., 2016, Markram et al., 2015, Potjans and Diesmann, 2014, Traub et al., 2005) that enable investigation of the mechanisms underlying circuit function. Biologically detailed circuit models are necessarily complex and typically have a large number of parameters. Experimental measurements from connectomics (Helmstaedter et al., 2013, Kasthuri et al., 2015), functional activity mapping (Ahrens et al., 2013), and multi-cell and automated patch clamping (Annecchino et al., 2017, Guzman et al., 2016), together with datasets from large-scale brain initiatives (Amunts et al., 2016, Hawrylycz et al., 2016, Insel et al., 2013, Kandel et al., 2013), are providing an increasingly wide range of data to constrain such models, thereby improving their accuracy. But confidence in the predictions from biologically detailed models is currently limited by their complexity and their perceived lack of constraint.

Constructing well-constrained models of neurons and circuits from a raw dataset takes a considerable amount of time and skill despite well-established simulation tools (Carnevale and Hines, 2006, Gewaltig and Diesmann, 2007, Goodman and Brette, 2008, Ray and Bhalla, 2008). Once built, the complexity of detailed models makes them difficult to modify for new scientific questions. Moreover, running large-scale circuit models often requires high-performance computing facilities, which may not be accessible to many end users and brings an additional layer of difficulty to setting up simulations and managing the resultant datasets. These technical barriers hinder access to the structural and functional properties of biologically detailed models, limiting scientific scrutiny and adoption of these powerful tools by the wider community.

Ensuring biologically detailed models are robust and error free is challenging given the length and complexity of their software implementations. Common errors include typos in equation definitions and parameter values, unit conversions, inconsistent use of temperature dependencies, and incorrect translation of reconstructed neuronal morphologies. In industry, open source software development is increasingly being used to create well-tested, modular software components and applications, which can be shared publicly using code development and collaboration platforms, such as GitHub (Perkel, 2016). GitHub records changes in the code and allows multiple users to manage and recombine different versions, track issues, and flag stable versions of the code. Errors can be minimized by regularly testing each modular component with automated routines and then assembling them into larger structures. In computational neuroscience, standardized modular frameworks (“model description languages”) have also been developed for specifying the biological components of circuits, such as ionic and synaptic conductances, neuronal morphologies, and synaptic connectivity (Cannon et al., 2014, Davison et al., 2009, Gleeson et al., 2010). These components could be used to build modular models that are easy to configure and test, facilitating their reuse for different scientific questions. However, adoption of strategies currently used in open source software engineering for creating, managing, testing, and validating modular code has been the exception rather than the rule for neural modeling in academia (Eglen et al., 2017).

To address these challenges, we have developed Open Source Brain (OSB) (http://www.opensourcebrain.org), a web-based collaborative resource for viewing, simulating, disseminating, and developing standardized models of neurons and circuits. OSB hosts a range of neuronal and circuit models from multiple brain regions, including the neocortex, cerebellum, and hippocampus. The morphology of modeled neurons, the structure and connectivity of networks, and the values of physiological parameters used can be automatically visualized in graphical form on OSB through a web browser. Moreover, functional properties can be explored by simulating models through the browser without installing programs or writing code. Unlike previous repositories, deep links between OSB and GitHub provide a collaborative resource for developing, refining, and automatically testing models, enabling them to evolve as new information becomes available. OSB functionality has been achieved by combining tools and best practices from the open source software development community, harnessing modern web technologies, and integrating them with standardized modular descriptions of models (Cannon et al., 2014, Davison et al., 2009, Gleeson et al., 2010). Using OSB and associated offline tools, it is possible to combine model components across different levels of biological detail. We illustrate this functionality by constructing models of multiscale inhibition-stabilized cortical networks (ISNs) (Tsodyks et al., 1997) and analyzing their robustness to different levels of biological detail. By making models more accessible and facilitating model development through collaboration, OSB provides an online resource of standardized models that can be critically evaluated and reused by the wider neuroscience community.

## Results

### The OSB Resource

**Figure 1:**
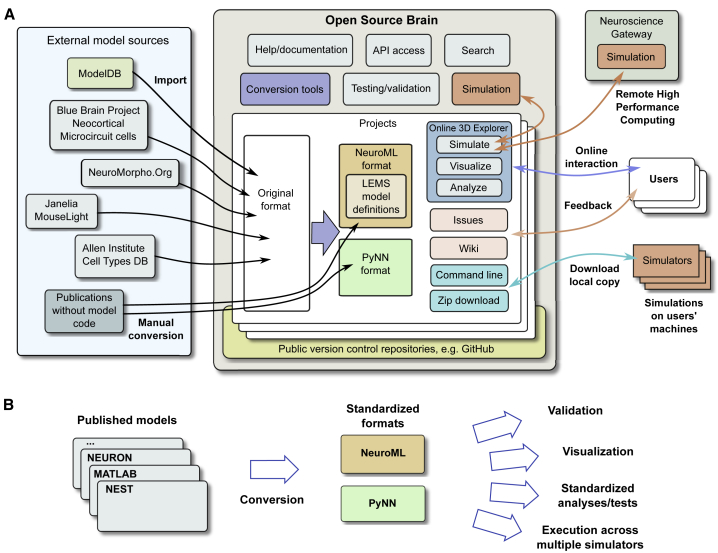
Overview of Open Source Brain (A) Functionality of Open Source Brain (OSB) and interactions with users and external resources. (Left) Model sources for OSB are shown. Center: OSB resources to facilitate conversion of models in open source repositories to standardized formats; to validate against the standards; to test model code; and to visualize, analyze, and simulate models through a web browser are shown. A search function is provided, together with an application programming interface (API). Right: user interaction with projects can be through the OSB web interface or by command line. Wikis enable feedback, and issues can be opened. Project code can be cloned, forked, or committed using standard open source workflows or downloaded as zipped releases. Simulations can be performed on the OSB server or submitted to the Neuroscience Gateway for execution on their supercomputing facilities. See also Figure S1. (B) Functionality following the conversion of published models from simulator-specific formats into standardized representations. This includes automated validation, visualization, analysis, and simulations on different platforms, using a variety of generic tools.

OSB is an online platform (http://www.opensourcebrain.org) that links open source repositories containing standardized models of neurons and circuits to users and developers. OSB provides powerful tools to visualize, analyze, simulate, develop, and test models through web browsers (Figure 1A). These features were made possible by defining models in the neuroscience model description languages NeuroML (Cannon et al., 2014, Gleeson et al., 2010) and PyNN (Davison et al., 2009). These standardized formats define the properties of models (e.g., biophysical parameters, cell morphology, and connectivity) in a modular, structured way. This enables model files to be automatically read by OSB and the physiological and anatomical details presented through the browser (Figure 1B). They also contain the information required to simulate the model, enabling the functional properties of individual neurons and networks (e.g., membrane potential and firing activity) to be explored. OSB provides access to metadata associated with the models, including the history of their development (provenance), and has links to wikis, allowing users to discuss their performance and any technical issues. The model code is hosted in public software development repositories (e.g., on GitHub), because these provide functionality to track and manage changes to the code. This combination of open source software development infrastructure and model standardization enables OSB to deliver up-to-date versions of models in accessible graphical formats (e.g., 3D views of cells and circuits, tables, and interactive plots) that can be understood and used by the wider neuroscience community.

**Figure 2:**
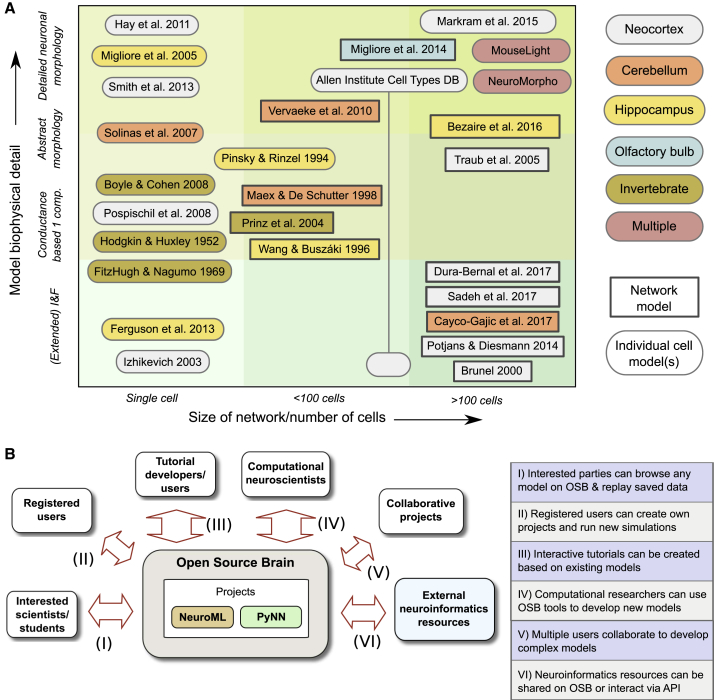
Standardized Multiscale Models on the OSB Platform, Together with User Interactions (A) NeuroML- and PyNN-based models on OSB, identified by author(s) of the original publications describing the models. The models have different levels of biophysical detail, ranging from simple point neuron models (e.g., integrate and fire [I&F]) to complex multicompartment cell models. Some projects contain single cells, and others contain multiple cell types or network models. Neuronal and circuit models cover a broad range of brain regions and include both vertebrate and invertebrate systems. More details on these models are given in Tables S1 and S2. See also Figure S2. (B) Usage scenarios for OSB projects containing standardized models in NeuroML or PyNN, depending on users’ goals and level of computational expertise.

OSB currently hosts standardized, curated models spanning a wide range of biophysical detail, varying from single cells up to large-scale networks with thousands of neurons (Figure 2A; Table S1). These models cover multiple regions of the brain, including neocortex (Brunel, 2000, Dura-Bernal et al., 2017, Hawrylycz et al., 2016, Hay et al., 2011, Izhikevich, 2003, Markram et al., 2015, Pospischil et al., 2008, Potjans and Diesmann, 2014, Sadeh et al., 2017, Smith et al., 2013, Traub et al., 2005), cerebellum (Cayco-Gajic et al., 2017, Maex and De Schutter, 1998, Solinas et al., 2007, Vervaeke et al., 2010), hippocampus (Bezaire et al., 2016, Ferguson et al., 2013, Migliore et al., 2005, Pinsky and Rinzel, 1994, Wang and Buzsáki, 1996), and olfactory bulb (Migliore et al., 2014). A number of invertebrate models have also been converted (Boyle and Cohen, 2008, Fitzhugh, 1961, Hodgkin and Huxley, 1952, Prinz et al., 2004). At the single-cell level, there are models from the Allen Institute Cell Types Database (Hawrylycz et al., 2016) and the Blue Brain Project (Markram et al., 2015) and reconstructed neuronal morphologies from the NeuroMorpho.Org (Ascoli et al., 2007) and Janelia MouseLight (Economo et al., 2016) databases. In addition to the standardized models presented here, there are a number of other user-contributed models on OSB that are in the process of conversion and curation. This community-driven approach encourages organic growth of models and components on OSB and ensures that the range of models available is determined by the interests of the users of the resource.

### User Interaction with OSB

There are a number of different ways users can interact with models on OSB, depending on their goals and level of expertise in computational neuroscience and in programming (Figure 2B). Scientists interested in rapidly learning about the properties of a model used in a scientific study can readily inspect model structure and parameters and replay previously recorded simulations through their browsers without registering as an OSB user. The main OSB projects page (http://www.opensourcebrain.org/projects) provides links to a wide range of models, including all of those presented here. After registering and logging in, users can run and store their own simulations for a more in-depth analysis of the functional properties of the model.

OSB can also be used to develop online resources for teaching neuroscience to students and researchers. This is facilitated through interactive help functionality and a framework for building tutorials, which can be used to illustrate the biophysical, anatomical, and physiological properties of a model and to help explain different mechanisms, such as the conductances underlying the action potential and synaptic integration.

Scientists wishing to use the infrastructure for model development, collaboration, testing, and dissemination form the core OSB user group (Figures 1A and 2B). Figure S1 provides an overview of the steps required to add a model to OSB and the tools we have developed to facilitate this. Once the model is converted to a standardized format, users are able to use the OSB tools for visualization, automated analysis, and testing to help evaluate the accuracy of their code and minimize errors. This facilitates model refinement by ensuring that the intended behavior is not disrupted after each modification. The OSB infrastructure and associated tools can also support larger scale collaborative projects to build and test more complex models. OSB interacts with other neuroinformatics platforms, enabling content to be shared between resources. For example, there are deep links between OSB and ModelDB (McDougal et al., 2017), an archive of neuronal models in their original published formats. These features of OSB enable neuroscientists from many backgrounds to explore and use biologically detailed models and lower the technical barriers to the more advanced features of the platform.

### Visualization and Analysis of Model Structure

**Figure 3:**
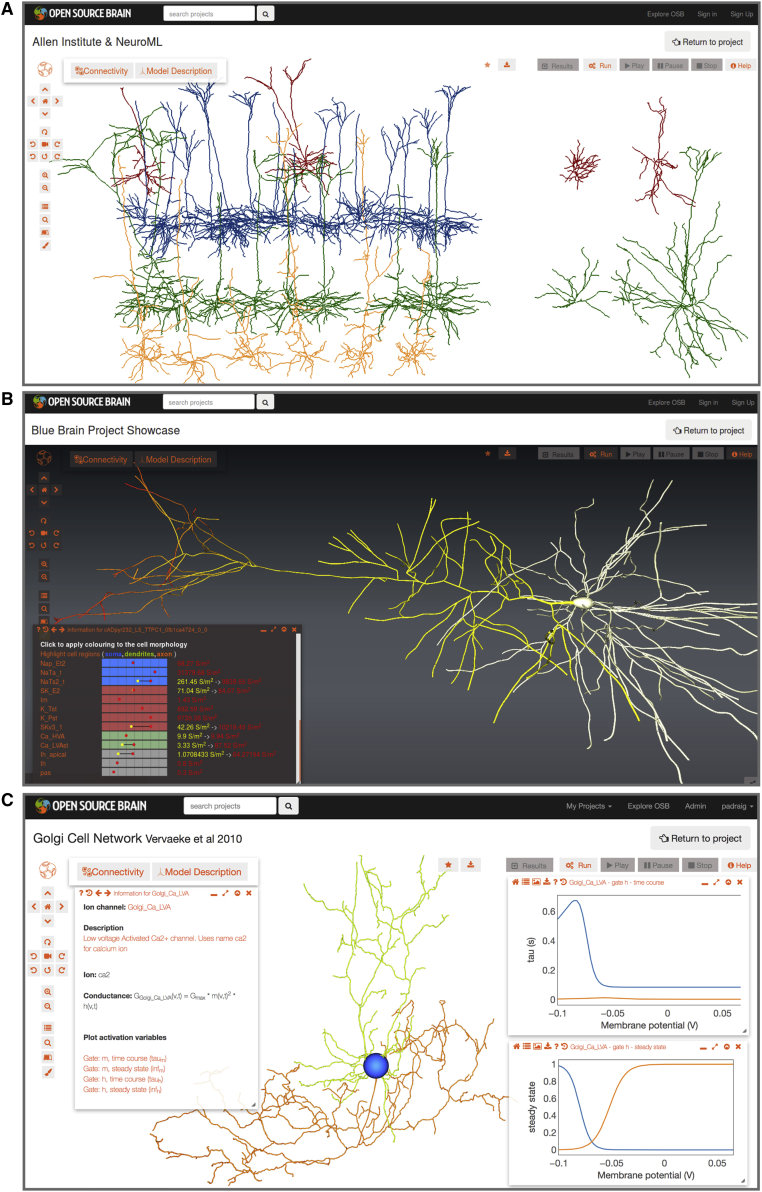
Visualization of Models through the Browser (A) Screenshot showing 37 cell models of visual cortex neurons from the Allen Cell Types Database on the OSB website, visualized in 3D through a browser. Spiny (32 on left) and aspiny (5 on right) cells from layers 2/3 (red), 4 (blue), 5 (green), and 6 (orange) are shown. (B) Layer 5 pyramidal cell from the Blue Brain Project neocortical microcircuit model. Bottom left: an information panel (opened via Model Description button) summarizing the types and densities of ionic conductances on the cell membrane is shown. Individual conductances can be clicked to highlight the regions of the cell where they are present. Cell morphology shows the non-uniform distribution of the hyperpolarization-activated conductance on the apical dendrite (low = yellow near soma; high = red in the distal dendrites). (C) Cerebellar Golgi cell model from Vervaeke et al. (2010). Cell regions have been highlighted (blue soma, green dendrites, and orange axon). Left information panel for a low-voltage-activated Ca^2+^ conductance (Ca LVA) present on the cell is shown, including conductance expression and gating variables. Right plots show voltage dependences of time constant (top) and steady-state value (bottom) for the activation (orange, m) and inactivation (blue, h) gates. Dendrite and axon diameters are increased for clarity of figure presentation in (A)–(C). See also Video S1.

To allow visualization of a model in the web browser, OSB searches for the standardized model descriptions (NeuroML files) in the repositories associated with the OSB project. This information is used to generate a 3D visual representation of the neuronal morphology and/or the circuit structure (Figure 3A). In addition, the spatial distribution of the density of ionic conductances can be viewed either in tabular form or as a pseudocolor density map superimposed on the neuronal morphology (Figure 3B). Because models of ionic conductances are also specified in NeuroML format, the underlying mathematical expressions defining the rates of activation and inactivation can be extracted and plotted (Figure 3C). Thus, the types, distributions, densities, and kinetic properties of the membrane conductances present in the model can be automatically exposed in graphical formats. Other useful information in the NeuroML files, such as authors of the files, references, and links to the original data sources, is also presented through the web interface. This facilitates transparency and enables the history of models and their authors to be recorded (provenance tracking).

**Figure 4:**
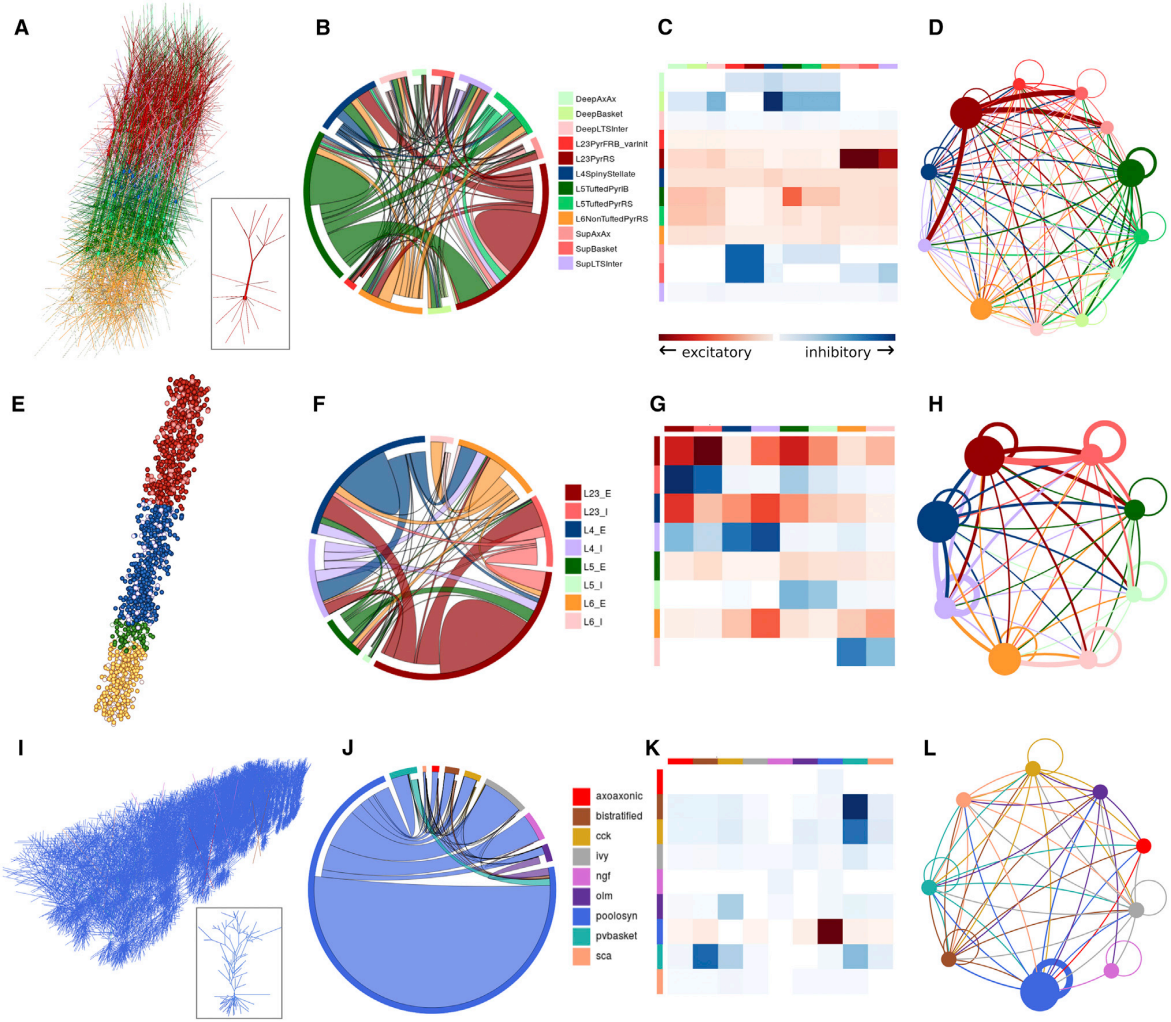
Analysis of Network Structure on OSB (A) Single-column thalamocortical network model from Traub et al. (2005), containing 336 cells (between 100 and 148 compartments each; 10% of full network) in 12 populations. Inset shows example layer 2/3 pyramidal cell. (B) Chord diagram showing projections between populations (1 × 10^5^ individual connections of 193 types). Outer ring color indicates population; the chords are attached to the presynaptic population outer ring segment, separated from the postsynaptic population ring segment and colored to match the postsynaptic population. Colors for populations match 3D view in (A). (C) Adjacency matrix; lines on left and top indicate pre- and postsynaptic population colors, respectively. Squares represent relative strengths of excitatory (red) or inhibitory (blue) inputs (average weighted conductance of synaptic input from the presynaptic population to each postsynaptic cell). (D) Connectivity graph between populations; relative size of populations indicated by circle diameter; connection line widths scaled by weight. Both circle size and line weight have minimum values for visual clarity. Line colors match presynaptic population. (E–H) 3D view of network (E), chord diagram (F), adjacency matrix (G), and connectivity graph (H) for point neuron spiking network model of Potjans and Diesmann (2014), with 1,539 cells in 8 populations (E [excitatory] and I [inhibitory] from layer 2/3, layer 4, layer 5, and layer 6; 5.96 × 10^4^ connections; 2% of full-scale network). (I–L) 3D view of network (I), chord diagram (J), adjacency matrix (K), and connectivity graph (L) for network model of hippocampal CA1 region from Bezaire et al. (2016) with 311 pyramidal cells (example cell in inset of I) and 24 interneurons of 8 different types. Network (0.1% of full scale) has over 1 × 10^6^ connections, primarily between pyramidal cells. All images are screenshots from browser visualization. See also Video S2.

The 3D structure of circuit models is often complex, as it can include multiple neuronal layers, a range of cell types distributed at different densities, and extensive synaptic connectivity. OSB facilitates visualization of network structure by automatically generating multiple types of connectivity diagrams. This is possible because NeuroML descriptions of such networks contain structured lists of 3D locations of somata and the subcellular location of chemical and electrical synapses. Figure 4A shows a single-column thalamocortical model consisting of multicompartmental neurons distributed over multiple cortical layers (Traub et al., 2005). The synaptic connectivity of such circuits can be inspected using automatically generated visualizations. A chord diagram (Figure 4B) provides a convenient way to assess the density or sparsity of the synaptic connectivity. In contrast, the connectivity matrix (Figure 4C) provides a more quantitative overview of the synaptic connections, showing the strength of excitatory and inhibitory connections between different cell populations. Lastly, the connectivity plot shown in Figure 4D combines these features in one plot, providing a way to visualize the size of the neuronal populations, the connections between them, and their relative strength. This functionality enables the easy comparison of network connectivity. For example, a cortical network consisting of point neurons (Potjans and Diesmann, 2014) can be analyzed and compared with the previous, more detailed cortical model (Figures 4E–4H). For large-scale networks with a high level of biological detail, such as the recently developed CA1 circuit model (Bezaire et al., 2016), OSB can progressively load parts of the network to speed visualization. For example, visualization of the gross structure of the circuit does not require loading the synaptic connectivity matrix (Figure 4I). However, this can be loaded in the background if required, enabling the properties of the synaptic connectivity to be visualized (Figures 4J–4L). These features substantially extend the options available for exploring model structure when compared to the original versions of these models, because this information was buried deep within the specialized code (Fortran, NEST SLI, and NEURON hoc in Figures 4A–4D, 4E–4H, and 4I–4L, respectively). Videos S1 and S2 illustrate interactive exploration on OSB of the models shown in Figures 3 and 4, respectively.

Video S1. Recording of Models Being Visualized on OSB, Related to Figure 3 Sequences captured from web browser showing visualization in 3D on OSB of: cell models from Allen Institute Cell Types Database (Hawrylycz et al., 2016) (Figure 3A); a layer 5 pyramidal cell from the Blue Brain Project (Markram et al., 2015) (Figure 3B) showing information on active conductance distributions; a cerebellar Golgi cell model (Vervaeke et al., 2010) (Figure 3C) with plots for channel gating variables. Some frames from recordings have been removed to shorten videos.

Video S2. Network Models Visualized and Analyzed on OSB, Related to Figure 4 Sequences captured from web browser with visualization and analysis of network models: thalamocortical network model (Traub et al., 2005) (Figure 4A) showing interactive adjacency matrix; point neuron cortical network (Potjans and Diesmann, 2014) (Figure 4E) illustrating highlighting of connections to/from individual cells and chord diagram of all connections; network model of CA1 region of hippocampus (Bezaire et al., 2016) (Figure 4I) showing how populations can be hidden and population connectivity graph. Some frames from recordings have been removed to shorten videos.

### Functional Properties of Models Revealed through Online Simulation

**Figure 5:**
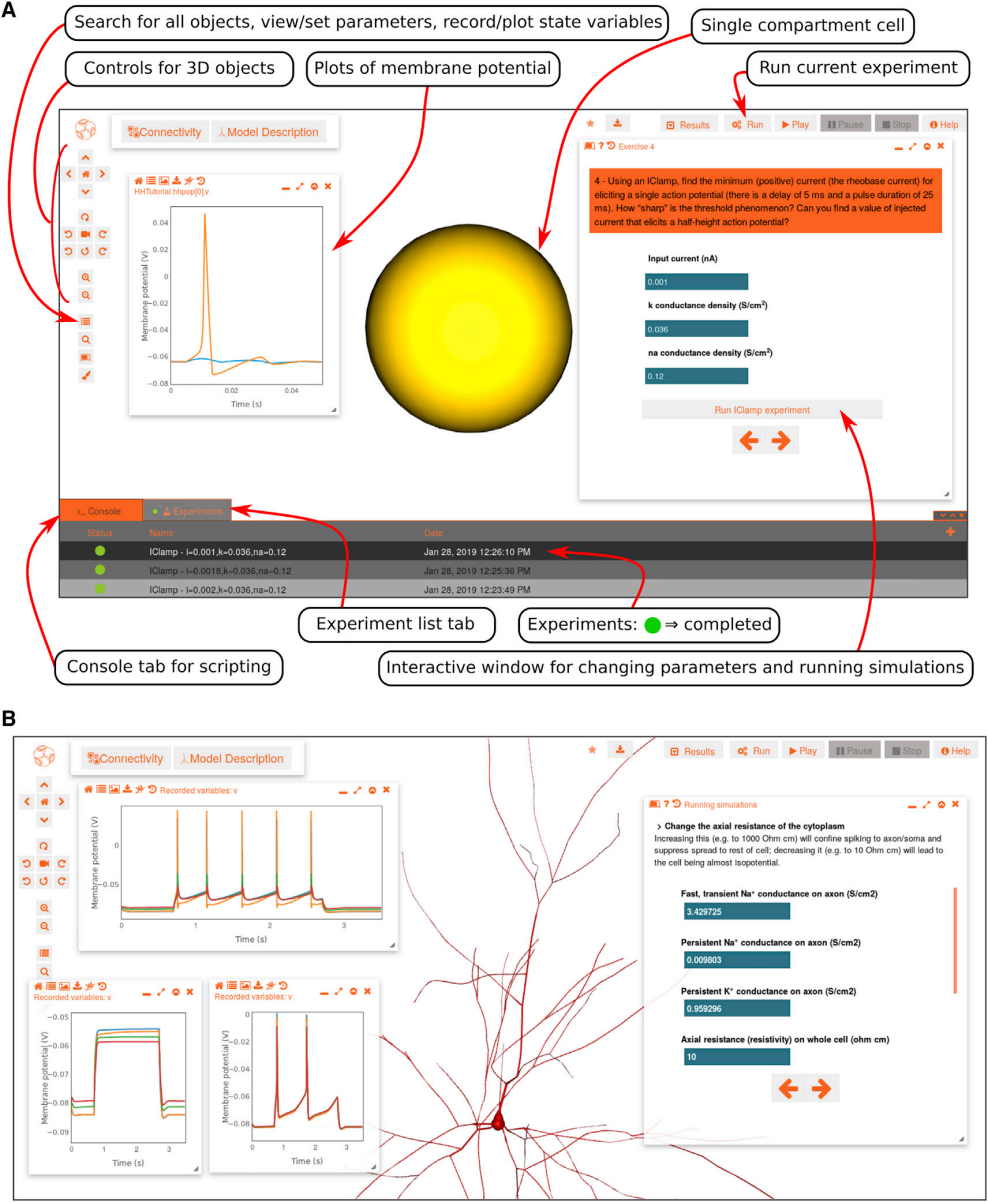
Interactive Online Tutorials Illustrating Control and Execution of Simulations (A) Annotated screenshot of an OSB project for a single-compartment neuron with Hodgkin-Huxley type conductances (Hodgkin and Huxley, 1952). Single-compartment model cell (yellow sphere), tutorial control panel for altering current, channel densities, and running simulations (right), list of previously run experiments with changed parameters (bottom), tab for enabling the interactive command line console (bottom left), and membrane potential plot showing spiking (orange) and subthreshold (blue) recordings (left) are shown. (B) Screenshot of interactive tutorial using a layer 2/3 pyramidal cell model (Markram et al., 2015) to illustrate how OSB represents biophysically detailed cells and how their functional properties can be explored. Right: interactive guide shows parameters that can be changed and suggestions for exploring behavior. Plots on left show membrane potential at multiple locations on cell (blue, soma; orange, end of axon; green and red, two dendritic locations) for 3 scenarios while 2-s current pulse is applied: original cell parameters (top); axonal sodium conductance removed (bottom left); and axial resistance reduced by factor of 10 (bottom right). See also Video S3.

**Figure 6:**
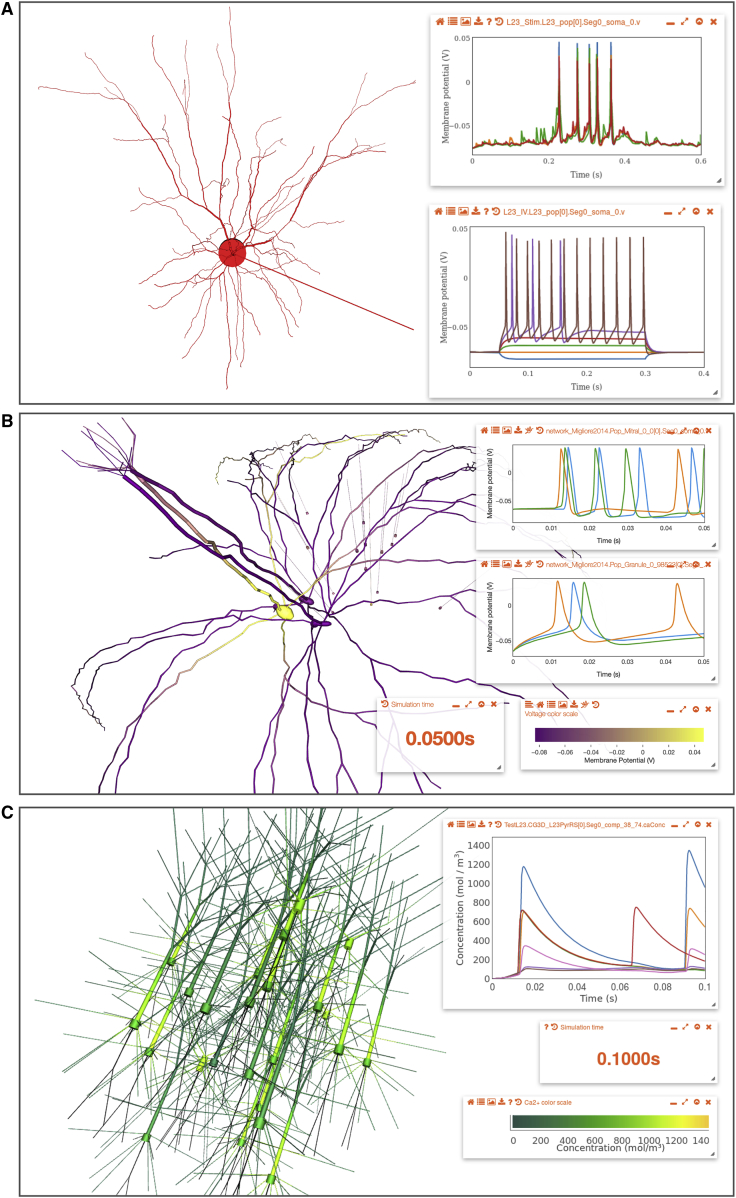
Visualization of Simulations through the Browser (A) Layer 2/3 pyramidal cell project from Smith et al. (2013). Left: 3D cell morphology is shown. Top right: membrane potential recorded at 5 locations from a simulation of this cell receiving background synaptic stimulation is shown. Bottom right: membrane potential at soma for increasing levels of current injected at the soma is shown. (B) Small network with 3 mitral and 15 granule cells from Migliore et al. (2014). Plots on right show somatic membrane potentials for 3 mitral (top) and 3 granule (bottom) cells. Panels below show current simulation time and color scale for the recorded membrane potentials, as displayed on the cell morphologies during simulation replay. (C) Left: small network of layer 2/3 pyramidal cells and interneurons from Traub et al. (2005). Right hand plot shows time course of somatic calcium concentration in 7 cells during the simulation. Right bottom, scale for recorded calcium concentration as overlaid on morphologies is shown. See also Video S3.

To make the functional properties of models of neurons and circuits more accessible to the wider community, we have developed browser-based simulations on OSB, which remove the requirement to write code. This functionality is enabled by the simulator-independent nature of the standardized formats of models on OSB. Instructions for simulating the model are fed to the OSB server, where the code for running the simulation is automatically generated and executed (typically using the NEURON simulator; see STAR Methods). Short simulations can be run quickly on computing resources provided by the OSB server, and larger scale computations can be easily submitted for execution through the Neuroscience Gateway at the San Diego Supercomputer Center (Sivagnanam et al., 2013), which provides parallel execution of models across hundreds of processors (Figure 1A; STAR Methods). Upon completion, the data generated are sent back to the browser for visualization (Figures 5 and 6). These features enable exploration of complex circuit models without the requirement for specialist knowledge to setup and run large-scale simulations (Video S3).

Video S3. Simulation Execution and Replay on OSB, Related to Figures 5 and 6 Sequences captured from web browser illustrating: manually executing and reloading a short simulation of a point neuron based on the Hodgkin Huxley model (Hodgkin and Huxley, 1952) (Figure 5); interaction with same model, but through custom tutorial panel allowing current and voltage clamp experiments to be set up and run; layer 2/3 pyramidal cell model (Smith et al., 2013) (Figure 6A) with multiple locations on the cell specified for recording; small network of cells from olfactory bulb model (Migliore et al., 2014) (Figure 6B) where all segments of multicompartmental cells have had membrane potential recorded, which can be replayed superimposed on cells; reduced version of thalamocortical network model (Traub et al., 2005) (Figure 6C) where [Ca^2+^] has been recorded and replayed on cells. Some frames from recordings have been removed to shorten videos.

Users can also alter values of model parameters through the browser, such as current injection levels and densities of ion conductances (Figure 5). By running multiple simulations, this enables characteristic neuronal properties to be investigated (Figure 6A). More substantial changes to the model, such as adding new conductances or changing the number of cells, currently require offline regeneration of the NeuroML files (Figure S1). Nevertheless, many changes can already be made to investigate cell and network behavior, such as setting a synaptic conductance to zero to remove the connection between two specific populations. The user also has control over the number of simulated variables recorded. For example, the membrane potential can be recorded from the soma or from all compartments in every cell. Recorded data can be replayed as variable-time plot or a pseudocolor representation can be used to indicate the voltage (Figure 6B) or calcium concentration (Figure 6C) across the morphology or across a population of neurons. The ability to analyze, visualize, and interact with models on OSB provides a unified online resource for accessing the structural and functional properties of complex models of brain function, thereby enabling greater scrutiny and insight into these powerful computational tools.

### Simulation Management and Tutorials

Simulations of neurons and circuits generate a large amount of data. Moreover, to examine behavior under different conditions, models must be run many times. To deal with these requirements, we have built a system for managing and storing simulation experiments on OSB. This enables registered users to run multiple simulations and to interactively explore the results (Figure 5). The simulation results generated through OSB can also be downloaded in a zip file to the user’s computer or automatically uploaded to Dropbox for more detailed offline analysis (STAR Methods). In addition, the layout of the visualization panels showing the 3D morphology of the model and associated analysis panels can be saved between sessions. All changes to the model and its graphical visualization are recorded as a series of text-based instructions, ensuring that the simulation, analysis, and presentation are fully documented (STAR Methods). These can be accessed through a popup console tab (Figure 5A, bottom left), which allows a series of instructions to be copied, pasted, and rerun, as well as direct control of OSB through scripting.

The web-based nature of OSB, together with its simulation and management features, make it well suited for demonstrating the principles of neurophysiology in an interactive and accessible format. To this end, we have built a framework for constructing online tutorials that can be used to explain concepts through presentation of figures and simulations. These features enable interactive tutorials and virtual experiments to be constructed that can be used to teach basic concepts in neurophysiology and computational neuroscience without the barrier of having to write code or install specialist simulators. To illustrate this functionality, we have extended a pre-existing tutorial on the Hodgkin Huxley model of the action potential for use on OSB (Figure 5A) and have created an interactive tutorial on modeling biophysically detailed, multicompartmental neurons using a layer 2/3 pyramidal cell from the Blue Brain Project (Markram et al., 2015; Figure 5B).

### Adding Models to OSB

When a new project is added to OSB in a standardized format, the contributor immediately benefits from the automated visualization, analysis, and simulation to showcase their own model. Standardized formats also aid analysis and comparison of the properties and behavior of the cells from different sources (Figure S2). Although some models are originally developed in standardized formats (Cayco-Gajic et al., 2017), most existing models have been developed and defined in simulator-specific languages (McDougal et al., 2017) and therefore require conversion to NeuroML or PyNN. NeuroML is a widely used standardized model description language that is sufficiently flexible to define a wide range of models in neuroscience (Cannon et al., 2014, Gleeson et al., 2010). Models defined in NeuroML can be automatically “read” and visualized or transformed into the instructions required to run simulations (Table S2). PyNN is a Python-based language for describing models that is compatible with a range of simulators, including NEURON (Carnevale and Hines, 2006), NEST (Gewaltig and Diesmann, 2007), Brian (Goodman and Brette, 2008), and neuromorphic hardware (Schemmel et al., 2010). Although PyNN and NeuroML have different approaches to model specification, they are interoperable: networks can be created with PyNN scripts and the structure exported to NeuroML format (e.g., Figures 4E–4H) and specific cell models in NeuroML can be used in PyNN scripts and run on supported simulators (STAR Methods).

We have developed a range of documentation and tools to facilitate the conversion of models into NeuroML and PyNN (Figures 1A and 1B). Figure S1 provides an overview of how these tools can be used at each stage of conversion of an existing model for use on OSB. A key advantage of the modular structure of NeuroML and PyNN is that model components can be automatically tested across multiple simulators using the OSB Model Validation (OMV) framework (STAR Methods). This allows automated tests to be run to check the expected behavior of models every time there is a change to the code in the repository and helps ensure the quality of the model components. Table S2 shows the range of simulator-specific tests on the OSB models discussed in this paper. To facilitate local execution and testing of models, we have created a self-contained software environment (a Docker image; STAR Methods; Table S3) with all simulator tools preconfigured (Table S4), as well as verified, stable releases of all models presented here. Using this tool, 351 individual tests across 23 simulator configurations in 27 projects can be run on any operating system supporting Docker (Table S3). This demonstrates the broad coverage of model types and simulators that can benefit from automated testing and will help ensure OSB models and components are reproducible.

### Creating New Models from Existing Components on OSB

A key reason to make models and components available on OSB is that they can be reused and adapted to address new scientific questions. NeuroML software libraries (STAR Methods) can be used to create new models by reusing pre-existing components. To illustrate this, we built new cortical network models with differing biological detail by combining existing components using the tools we have developed for construction and optimization of NeuroML-based models (Figure S1). Linking the resulting model to OSB then enabled the visualization, management, and testing functionality to be used to adapt and refine the models.

Based on the connectivity and functional properties of the neocortex, it has been suggested that cortical networks operate in a regime with high excitatory gain, which renders the excitatory subnetwork unstable in the absence of strong feedback inhibition (Tsodyks et al., 1997). There is considerable interest in such inhibition-stabilized network (ISN) models (Garcia Del Molino et al., 2017, Joglekar et al., 2018, Ozeki et al., 2009, Rubin et al., 2015, Sadeh et al., 2017), as high-gain network regimes are thought to contribute to important functions, like signal amplification, noise tolerance, and pattern completion, and could underlie certain pathological states, such as epilepsy (Avoli et al., 1995, Mann et al., 2009). Moreover, recent experimental studies on the superficial layers of visual and auditory cortex support the idea that they operate as ISNs (Adesnik, 2017, Kato et al., 2017, Moore et al., 2018). Networks operating in ISN regimes can be identified through their characteristic “signature,” which is a paradoxical inverse response of the inhibitory interneurons to alterations in excitatory drive (Tsodyks et al., 1997). This was predicted from highly simplified models where neuronal populations were modeled as single nodes and synaptic input was modeled as current. The simplicity of such models raises the question of whether more complex neuronal networks, composed of populations of excitatory and inhibitory neurons interconnected with more realistic recurrent synaptic connectivity and conductance-based signaling, behave in a similar manner (Sadeh et al., 2017). Moreover, real cortical neurons receive synaptic input onto extensive dendritic trees, which can exhibit nonlinear behavior (Stuart and Spruston, 2015). However, no previous model has explored whether ISN properties can be detected in neurons with realistic dendritic integration.

**Figure 7:**
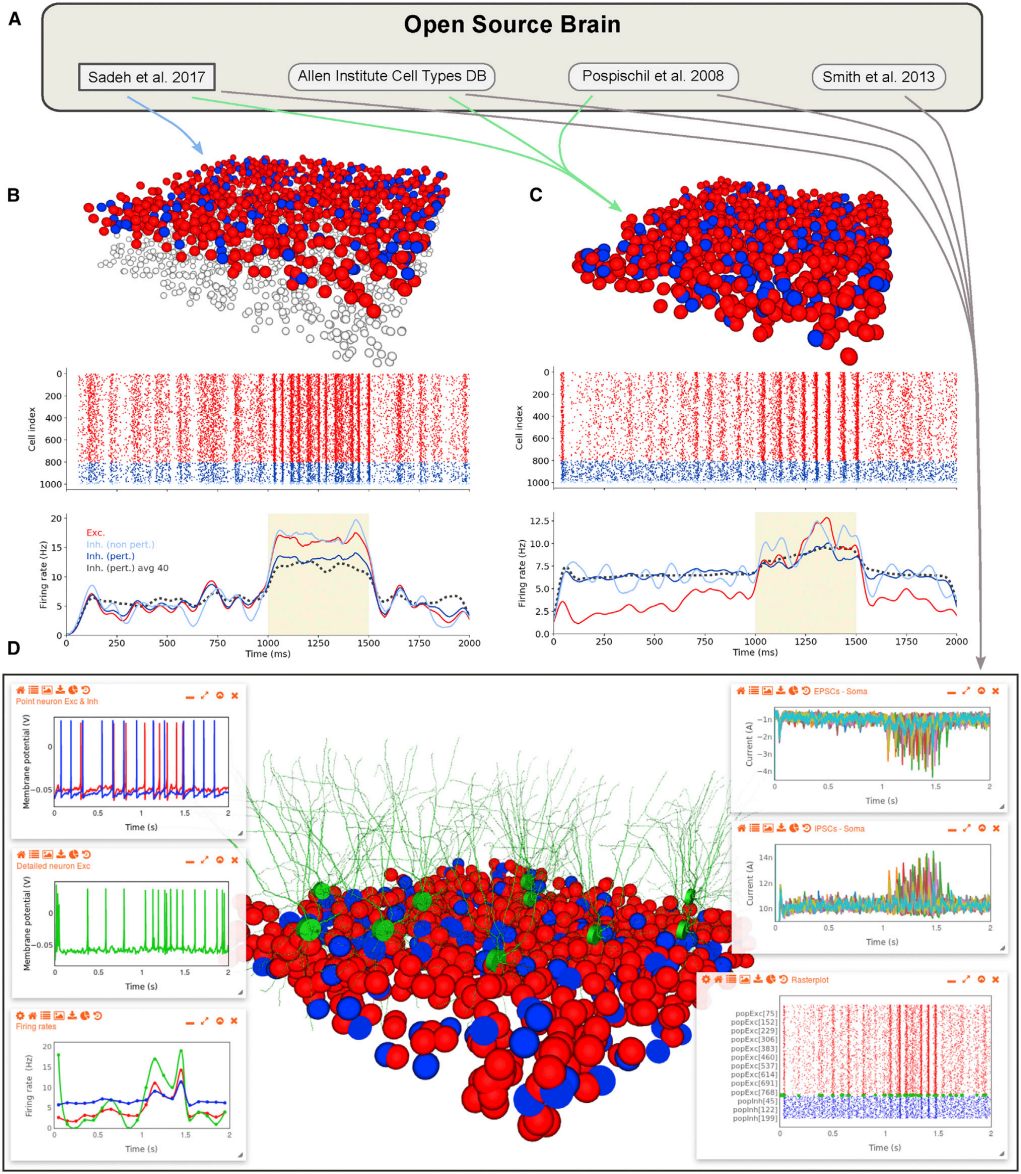
Creation of Biologically Detailed Models of Inhibition Stabilized Cortical Networks from Model Components on OSB (A) OSB models that have been reused to build inhibition stabilized network (ISN) models with different levels of biophysical detail. (B) Model of ISN created in PyNN, exported to NeuroML, and visualized on OSB (top; 800 excitatory [E; red], 200 inhibitory [I; blue] cells; external spiking inputs, modeled as explicit populations in PyNN, shown in white). Spiking behavior of cells (middle) and population rate plots (bottom) during reduced excitatory synaptic excitation to 90% of the I cells (during shaded period input rate was reduced from 9,600 Hz to 9,200 Hz in these cells) are shown. Population rate plots (bottom; average firing rate of subpopulations smoothened with Gaussian kernel of width 30 ms) reveal firing mean rate increases despite lower input to these cells (dark blue; black dotted line is average of 40 simulations), as well as the rate of non-perturbed I (light blue) and E cells (red), confirming the presence of a signature ISN response under these conditions. (C) Network model in NeuroML consisting of point neurons with voltage-gated membrane conductances from Pospischil et al. (2008) that were scaled to match the firing behavior of layer 2/3 spiny (E; red) and aspiny (I; blue) neurons from the Allen Cell Types Database. Network visualization, spike raster, and firing rate plots during a decreased excitatory drive to 90% of inhibitory cells as for (B) are shown. See also Figures S3A–S3D. (D) Similar network to (C), with 10 of the E cells replaced by detailed layer 2/3 cell model from Smith et al. (2013); green cells in 3D view). A single screenshot of OSB showing the range of graphical elements that can be used to interactively investigate the behavior of the network. Top two plots on left show membrane potentials from a point E neuron (red) and an I cell (blue) as well as the detailed E cell (green). A spike raster plot (bottom right) and corresponding firing rate traces (bottom left; average rate in 100-ms window per population) are also shown (population colors follow 3D network). The top and middle right-hand plots show currents from 10 independent network simulations when the somata of two morphologically complex cells were voltage clamped at −80 mV and 0 mV, revealing the excitatory and inhibitory postsynaptic currents, respectively. See also Figures S3B and S4.

To test whether ISN signature behavior is to be expected in real cortical circuits, we built three network models of increasing biological detail using NeuroML components present on OSB and the associated tools for model construction (Figure 7A; STAR Methods). Reimplementation of the spiking network model from Sadeh et al. (2017) in PyNN, with adaptive exponential integrate-and-fire (I&F) cells, exhibited *increases* in the firing when the excitatory drive onto inhibitory cells was *decreased* (Figure 7B; cf. Figure 10B in Sadeh et al., 2017), hence confirming that the paradoxical signature of ISN could also be observed in these I&F networks. To investigate whether ISN responses occur with more realistic conductance-based spiking mechanisms, we reused the combination of membrane conductances from cortical cell models in Pospischil et al. (2008) and created single-compartment cell models that matched experimentally recorded behavior of layer 2/3 spiny (putative excitatory) and aspiny (putative inhibitory) cells from the Allen Cell Types Database (STAR Methods; Figures S3A–S3D). A network model constructed with these cells and with synaptic connectivity from Sadeh et al. (2017) also exhibited the paradoxical effect of ISNs (Figure 7C).

To examine whether dendritic integration affects the ability to detect ISNs, we built a hybrid network model where some of the single-compartment excitatory cells were substituted with morphologically detailed layer 2/3 pyramidal cell models (Smith et al., 2013; Figures S3E and S4A). Figure 7D shows a screenshot of OSB with a 3D view of the hybrid network and a number of visualization panels showing the network activity, including raster plots, firing rate traces, and membrane potential plots for individual cells. As for the networks of point neurons, this hybrid network exhibited an increase in firing rate upon reduced excitatory drive to inhibitory cells, with a particularly strong effect observed in the morphologically detailed neurons. Voltage-clamp recordings from 2 of the 10 morphologically complex cells revealed a burst of excitatory and inhibitory synaptic currents during the period of reduced excitatory drive to a subset of inhibitory cells (Figure 7D, top and middle panels on right). Similar responses were observed when a hyperpolarizing current was applied to interneurons to mimic optogenetic inactivation by halorhodopsin (Figures S4B–S4D), consistent with recent voltage-clamp recordings from layer 2/3 cortical pyramidal neurons (Kato et al., 2017).

These results show that ISN signature responses can occur in network models with large populations of excitatory and inhibitory cells interconnected with the extensive recurrent synaptic connectivity and conductance-based signaling mechanisms as found in cortical networks. Moreover, they establish that the ISN signature responses can be detected from somatic voltage-clamp recordings in layer 2/3 pyramidal cells, even when the synaptic input is integrated across the dendritic arbor. These results demonstrate that components of NeuroML models on OSB can be reused with the new tools and infrastructure to build complex multiscale circuit models.

## Discussion

We have developed Open Source Brain, a web-based collaborative resource of standardized neuronal and circuit models together with tools and infrastructure for model development, testing, and reuse. The OSB platform enables web-browser-based visualization, analysis, and simulation of models without the need to install software or write code. This makes complex models accessible to the wider neuroscience community, enabling critical evaluation of model properties and behavior. The modular format used by OSB ensures that models and their components can more easily be reused for new scientific questions. By making neuronal and circuit models more accessible, transparent, and reliable, the OSB platform provides a powerful new resource for students, individual researchers, and collaborative research teams to learn about and investigate brain function in health and disease.

OSB’s browser-based visualization of models of neurons and circuits and automated analysis of their structural and functional properties provides a wealth of information about model properties that was largely inaccessible to non-specialists. Moreover, by removing the technical barriers of having to write code to run and configure simulations on high-performance computing facilities, OSB’s browser-controlled simulation functionality now makes it possible for anyone to explore the behavior of complex models. Advanced users have option of downloading any of the models and using the same standards-based toolchain on their own machines (STAR Methods). Academics can also use the OSB online tutorial building functionality to build interactive teaching resources that combine text and simulations to illustrate diverse neurophysiological phenomena. Allowing a wider range of users to access such detailed models will facilitate critical evaluation from the wider neuroscience community.

OSB is designed so that, when a new project is added by a contributor, they immediately benefit from functionality to showcase their own model (Figure S1). An important aspect of OSB is the provision of infrastructure to facilitate the continuous open source development, refinement, and testing of models. This is enabled through standardized model descriptions, new tools for automatically testing code, and deep integration with the code development platform GitHub, which enables collaborative software development. The OSB Model Validation framework, which uses code-testing methodologies from software engineering, helps ensure model behavior does not change when converted to standardized formats and when updates are made to the code. This can maintain code quality and consistency, enabling complex models to be kept up to date with new experimental results, without introducing errors. The large battery of tests that have already been applied across models and components by this framework (Table S2) enables an unprecedented level of reproducibility to be obtained on OSB.

The fact that model development and testing can be carried out without the need for OSB administrators to get involved will enable the resource to expand in a way that is determined by the user’s interests and research goals. Models contributed by individual researchers and labs are complemented with community-developed models, including the Human Brain Project (HBP) HippoCamp initiative, for which our CA1 network conversion (Figures 4I–4L) is a first major contribution, and the OpenWorm project (Sarma et al., 2018, Szigeti et al., 2014), which aims to create a detailed computational model of the nematode *C. elegans*.

The distinct functionality of OSB extends and complements that of ModelDB (McDougal et al., 2017), a well-established repository of models in computational neuroscience. ModelDB hosts model code in the original language in which it was developed and facilitates the reproduction of the results from their originating publications. OSB builds on this functionality by focusing on hosting standardized models that are independent of the simulator used, which enable users to interact with and analyze models in greater detail. Moreover, OSB is designed to reveal circuit-level properties, including connectivity and network dynamics (Figures 4 and 7). More fundamentally, models hosted on OSB are not static as they are in ModelDB and can instead be developed, refined, and improved using the infrastructure for open source software development, automated validation, and testing of models. Deep links between these resources allow users to find the same model on either platform. Indeed, OSB actively encourages researchers to first submit their model to ModelDB following publication (Figure S1). The step of moving a model onto OSB is an indication that one or more parties (who may not be the original developers) wish to standardize and further develop the model, making it more accessible to the wider neuroscience community and extending it for use beyond the original publication. OSB also supports sharing of model code prior to publication, e.g., the ongoing development of a large-scale network model of primary motor cortex (Dura-Bernal et al., 2017).

OSB interacts with other online resources that provide structured, annotated data that are invaluable for creating and validating neuronal models. For example, reconstructed neuronal morphologies from NeuroMorpho.Org (Ascoli et al., 2007) and the Janelia MouseLight project (Economo et al., 2016) can be automatically converted to NeuroML and visualized through OSB, and these morphologies can become the basis for new models when combined with cell-specific membrane conductances already expressed in NeuroML. The Allen Institute Cell Types Database (Hawrylycz et al., 2016) provides electrophysiological recordings and morphological reconstructions from cells in mouse visual cortex. Biophysically detailed cell models and point neuron models based on these data are present on OSB, and new NeuroML-based models can be generated from the source data (Figures S3A–S3D). Models currently being used in the HBP (Amunts et al., 2016) have also been converted, including cell models from the Blue Brain Project’s reconstruction of the microcircuitry of rat somatosensory cortex (Markram et al., 2015), as well as a reduced version of a cortical column (Potjans and Diesmann, 2014). Converting the neuronal models present in these networks to standardized formats provides a valuable resource for developing new models of cortical circuits from modular, well-tested building blocks.

As our development of biologically detailed cortical network models illustrates, new models can be built from the existing standardized components present on OSB by optimizing them against available data (Figure 7). By using model elements from different projects to build ISN network models with different degrees of biological detail, we show that the ISN signature responses that were predicted from highly simplified models (Sadeh et al., 2017, Tsodyks et al., 1997) are also expected in biologically detailed models that include conductance-based spiking mechanisms and can be detected in cells with complex dendritic morphologies. Predictions from these models can be used to refine experimental approaches for detecting ISNs (Adesnik, 2017, Kato et al., 2017, Moore et al., 2018), to explain why they might not be detectable under some experimental conditions, and to investigate how dendritic properties interact with the nonlinear dynamics of ISNs at the network level.

The models of neurons and circuits on the OSB platform complement standardization and online simulation in other areas of biology. In systems biology, biochemical signaling pathways, often expressed in standardized systems biology markup language (SBML) format (Hucka et al., 2003), can be analyzed and executed online by using, for example, VCell (Loew and Schaff, 2001) or on JWS (Olivier and Snoep, 2004). Expanding NeuroML’s existing functionality for interacting with SBML (Cannon et al., 2014) will allow easier integration of complex biochemical reactions into NeuroML-based models of neuronal and circuit models. It will also enable greater interoperability between OSB and databases such as BioModels (Le Novère et al., 2006) and the Physiome Model Repository (Yu et al., 2011). In addition to these subcellular model specifications, NeuroML is currently being extended to support more high-level, population-based models (e.g., the Wilson and Cowan model; Wilson and Cowan, 1972) which will potentially enable the analysis and simulation on OSB of brain-scale networks, as supported by a number of simulators, such as The Virtual Brain (Sanz Leon et al., 2013).

Building models from new and existing components currently involves configuring and optimizing the model offline and then loading the code to a repository on GitHub, where OSB can validate and test it prior to visualization, analysis, and simulation. In order to make this process easier, we plan to combine elements of the user interface of NetPyNE (a Python-based platform for model creation built on top of the NEURON simulator; Dura-Bernal et al., 2019) with OSB to provide an online model construction, optimization, and testing interface. This will be facilitated by the fact that the online visualization interfaces of both OSB and NetPyNE are built using the Geppetto platform (Cantarelli et al., 2018; STAR Methods). To further lower the barriers to model creation on OSB, we are also expanding the functionality of the platform to include tighter integration with the experimental data used to build models and test their performance. Standardized formats for experimental data, such as Neurodata without Borders (https://nwb.org), will be crucial for this. Hosting standardized models and the data from which they are built will provide all the necessary information for model optimization, thereby providing the functionality to refine and adapt models as new experimental results become available. Direct comparison of experimental data with model properties will provide a new level of transparency and scrutiny for data-driven models. These developments, together with the current functionality of OSB that facilitates accessibility and the construction of models from modular reusable components, will accelerate the pace of model building and reuse by the wider neuroscience community.

## STAR★Methods

### Key Resources Table

**Table:** 

REAGENT or RESOURCE	SOURCE	IDENTIFIER
**Software and Algorithms**		

OSB user management web interface	This paper	https://github.com/OpenSourceBrain/redmine
OSB visualization/simulation frontend	This paper	https://github.com/openworm/org.geppetto
NeuroML	Cannon et al., 2014	https://www.neuroml.org; RRID: SCR_003083
PyNN	Davison et al., 2009	http://neuralensemble.org/PyNN; RRID: SCR_002715
NEURON	Carnevale and Hines, 2006	https://www.neuron.yale.edu; RRID: SCR_005393
NetPyNE	Dura-Bernal et al., 2019	http://netpyne.org, RRID: SCR_014758
NEST	Gewaltig and Diesmann, 2007	https://www.nest-simulator.org; RRID: SCR_002963
Brian	Goodman and Brette, 2008	http://briansimulator.org; RRID: SCR_002998
MOOSE	Ray and Bhalla, 2008	https://moose.ncbs.res.in; RRID: SCR_008031

**Other**		

Allen Cell Types Database	Hawrylycz et al., 2016	http://celltypes.brain-map.org; RRID: SCR_015719
Blue Brain Project’s Neocortical Microcircuit Collaboration Portal	Ramaswamy et al., 2015	https://bbp.epfl.ch/nmc-portal; RRID: SCR_002994
NeuroMorpho.Org	Ascoli et al., 2007	http://www.NeuroMorpho.Org; RRID: SCR_002145
Janelia MouseLight project	Economo et al., 2016	http://ml-neuronbrowser.janelia.org; RRID: SCR_016669
Neuroscience Gateway	Sivagnanam et al., 2013	http://www.nsgportal.org; RRID: SCR_008915

### Contact for Reagent and Resource Sharing

For enquiries about any of the resources or models presented here, please contact the Lead Contact, R. Angus Silver, a.silver@ucl.ac.uk. For general queries about Open Source Brain, contact info@opesourcebrain.org.

### Method Details

#### OSB frontend: project and user management

The main OSB web interface (at http://www.opensourcebrain.org) is based on a heavily customized version of Redmine (http://www.redmine.org). This platform, developed in Ruby on Rails (https://rubyonrails.org), allows users to create accounts, make new projects and add other users to them, create user groups, and associate a version control repository (hosted on GitHub [https://github.com], BitBucket [https://bitbucket.org/], SourceForge [https://sourceforge.net], etc.) to each project. We have extended this framework with a new user interface, and added custom fields for projects with metadata relevant to the model, brain region, species, and simulators supported. Close integration with platforms hosting the version control repositories allows the files and change history to be deeply integrated into the OSB interface. Issues raised on GitHub, or forks of the repository are highlighted on the OSB page. Wikis describing the installation/usage of the models can be added directly on OSB, or content from README files in the associated repositories can be retrieved and displayed. An application programming interface (API) is provided to programmatically access information on all current projects and metadata (https://github.com/OpenSourceBrain/OSB_API). OSB project repositories are automatically searched for NeuroML files (in either XML or HDF5 format, see below) and these are presented to users for visualization through the browser.

#### OSB frontend: visualizing & simulating models

Visualization of models on OSB has been enabled through our contribution to the development of Geppetto (http://www.geppetto.org; Cantarelli et al., 2018), an open source modular framework built primarily in Java (server side) and JavaScript (browser side) that allows the content of files in various formats accessed on the server to be parsed, transformed, and visualized in the browser. The application was originally created in the OpenWorm project (Sarma et al., 2018, Szigeti et al., 2014), but it has developed into a modular platform with a number of parties contributing features (Cantarelli et al., 2018). The customized implementation of Geppetto for OSB provides extensive support for NeuroML models through the Java packages that we have developed (jNeuroML, see below). All that is required for interactive 3D visualization of these models in a web browser is WebGL (https://www.khronos.org/webgl), which is already present in most modern browsers. A number of JavaScript packages are used on the client side for 3D visualization (WebGL; three.js), user interaction (React; D3) and plotting (Plotly). In addition to NeuroML, Geppetto can interpret and display SWC format (Cannon et al., 1998) as well as 3D objects in OBJ format (http://www.martinreddy.net/gfx/3d/OBJ.spec).

The OSB implementation of Geppetto provides a canvas for displaying and interacting with 3D objects (Figures 3, 5, 6, and 7D) as well as a number of visualization panels for displaying textual information, interaction elements such as buttons and menu items, and plots. Connectivity information extracted from networks can also be displayed in custom visualization panels on OSB (Figure 4). Interaction with the 3D objects and visualization panels can be solely through the graphical interface, but OSB also provides an integrated console for interactions with all these elements through JavaScript (enabled by clicking on the Console tab at the bottom of the 3D view (Figure 5A)). An example usage is changing the representation of neuronal morphologies to cylinders and using a minimum radius for dendrites of 1 μm (as in Figure 3C): *network.setGeometryType(‘cylinders’,1)*.

#### NeuroML 2 & LEMS libraries

NeuroML version 2 (the version of the language supported by OSB) is built on a flexible low-level language, LEMS (Low Entropy Model Specification), which enables a wide range of physico-chemical processes to be defined (Cannon et al., 2014). To facilitate the use of NeuroML and LEMS models on OSB, we have developed a number of libraries for processing files in these standardized model description languages. These libraries have been developed in Java and Python, two of the most commonly used languages in computational neuroscience. A focus of the work has been on making these features available through easy to install packages. jNeuroML (https://github.com/NeuroML/jNeuroML) is a single package which gives access to all features which have been implemented in Java (Cannon et al., 2014). These include: natively parsing and simulating models specified in LEMS (including point neuron cell models and networks in NeuroML); converting NeuroML models to simulator-specific code (for currently supported simulators see Table S2); importing other structured formats to LEMS (particularly SBML (Hucka et al., 2003) models); validating NeuroML files, as well as performing basic tests for consistency; converting 3D models to SVG and PNG images.

libNeuroML (https://github.com/NeuralEnsemble/libNeuroML) is a Python package for reading, editing, and writing NeuroML files (Vella et al., 2014). pyNeuroML (https://github.com/NeuroML/pyNeuroML) is a Python package which builds on libNeuroML and bundles a copy of jNeuroML, allowing access to all of its functionality from Python scripts (most importantly converting NeuroML models to simulator code, running them, and reloading the results). Additionally, it has utility scripts for analyzing channel properties (*pynml-channelanalysis* for NeuroML channels; *pynml-modchananalysis* for NEURON channels) and *pynml-povray* for generating high resolution images and movies using POV-Ray (http://povray.org). Both jNeuroML and pyNeuroML can be used as command line applications (*jnml* and *pynml* respectively) or as libraries to give access to these features in other Java or Python applications (e.g., jNeuroML is bundled with neuroConstruct (Gleeson et al., 2007)). Both the Java and Python libraries can serialize NeuroML models as either XML or in binary HDF5 format (https://www.hdfgroup.org). The latter format has significant advantages in terms of file size (typically 10% of equivalent in XML) and speed of reading/writing, and can also be read from GitHub repositories for display in OSB.

Figure S1 illustrates how these libraries can be used during the process of converting and sharing models on OSB. All of these libraries are installed and configured for use with supported simulators in the OSB Docker image (Table S3; https://www.docker.com). Table S4 outlines the versions of the simulators, libraries, and programming languages used.

#### PyNN models

PyNN allows a single Python script to instantiate and run a network in either NEURON, NEST, Brian or on neuromorphic hardware (Schemmel et al., 2010); the only difference in the script is in the import of the package specific for that simulator (from *pyNN.neuron import ^∗^*, *from pyNN.nest import ^∗^*, etc.) (Davison et al., 2009). The NeuroML export from PyNN works similarly (*from pyNN.neuroml import ^∗^*) and will export the cell parameters, connectivity, and inputs to valid NeuroML. An example of this is the increasingly widely used (Cain et al., 2016, Lee et al., 2017, Schmidt et al., 2018a, Schmidt et al., 2018b, Schwalger et al., 2017) cortical column model (Potjans and Diesmann, 2014) in Figures 4E–4H. The original conversion of this model to PyNN was extended with 3D distributions for cell populations without changing the overall network behavior, but making the structure of the network clearer when visualized on OSB.

#### Executing simulations on OSB

Having models specified in NeuroML format on OSB means these can easily be converted to a number of simulator specific formats for execution (Table S2). A user who is signed in on OSB and is viewing a NeuroML model can “persist” the 3D view (orange star on top icon bar, e.g., Figure 3A, Video S3), so that that version of the NeuroML file (along with the layout/visual properties/currently open visualization panels) is stored for opening again at any time (there will be a list of persisted models on the user’s homepage when they log in). Simulations can be set up and run with a number of options including simulation duration, time step, what to record (e.g., all membrane potentials at cell somas), the numerical seed used for stochastic simulations, and which simulator to run it on. Currently supported simulators include NEURON, jNeuroML and NetPyNE (Dura-Bernal et al., 2019; http://netpyne.org). The latter is a Python package built on NEURON which greatly facilitates execution of robust, parallel network simulations in multiprocessor environments (see below). The OSB website is hosted on an Amazon Web Services (AWS, https://aws.amazon.com) instance, on which the supported simulators are installed. Short simulations can quickly be run on a single processor on this AWS platform. Users are presented with a list of running and completed simulations (Figure 5A) and can plot recorded values, overlay them on cells for visualization of cell and network dynamics (Figures 6B and 6C), or create rasterplots or firing rate traces to see population level activity (Figure 7D).

An alternative option for running simulations is enabled through OSB integration with the Neuroscience Gateway (Sivagnanam et al., 2013) (NSG, Figure 1A). OSB generates the simulator scripts and submits these through the RESTful API of NSG (NSG-R, http://www.nsgportal.org/guide.html). These are then submitted to the Extreme Science and Engineering Discovery Environment (XSEDE) high performance computing resources for execution, where NSG has pre-configured multiple neuronal simulation packages. Users on OSB do not require any account on NSG or XSEDE, and completed simulations appear in the OSB interface in the same way, albeit with more of a delay than running on OSB’s own servers. Because NSG provides access to parallel computing resources, OSB users can select NetPyNE as a target simulator, and opt to run network models across up to 256 processors, significantly speeding up the simulation time. This number can be increased in future as we gauge usage statistics/demand, to make greater use of the thousands of processors available via NSG. Scripts have also been developed which allow modelers to submit NeuroML and PyNN directly to NSG-R, bypassing the web interface (https://github.com/OpenSourceBrain/NSGPortalShowcase).

Data generated during simulations launched via OSB can be downloaded from the web interface, but can also be set to automatically be added to the user’s Dropbox (https://www.dropbox.com) folder, to enable local analysis of the simulation data. This is enabled by generating an API key on the Dropbox site and adding this to the user’s OSB account.

Existing tutorials for OSB are listed at http://www.opensourcebrain.org/tutorials. Documentation for those interested in developing and hosting tutorials and making use of the visualization and simulation features of OSB can be found at http://opensourcebrain.org/docs#Creating_Tutorials.

#### Interactions with neuroinformatics resources

The cell models used in the Blue Brain Project rat somatosensory microcircuitry network (Markram et al., 2015) have been made available in the original NEURON format on the Neocortical Microcircuit Collaboration Portal (NMCP) (Ramaswamy et al., 2015). We have developed scripts for automatically converting these cells to NeuroML and example models in this format have been made available on http://www.opensourcebrain.org/projects/blue-brain-project-showcase. We have also converted the ion channel models from the Channelpedia (Ranjan et al., 2011) database to NeuroML format in this OSB repository. Representative connectomes with point neurons used in recent studies of the Blue Brain Project microcircuit (Gal et al., 2017, Reimann et al., 2017) are available on the NMCP and a NeuroML HDF5 based version of this full connectome can be found on the OSB project. The full network (31346 cells, 7.6 million connections) cannot yet be displayed on OSB (though this is a target for future releases). However a scaled down version, with 5% of neurons in the original is available and can be visualized.

The Allen Cell Types Database (Hawrylycz et al., 2016) (http://celltypes.brain-map.org) contains neuronal reconstructions and electrophysiological recordings from multiple cells in mouse visual cortex (electrophysiological data samples shown in Figures S3A and S3C, see below). Computational models of these cells are also available on the website in both biophysically and morphologically detailed (implemented in NEURON simulator) and point neuron (Generalized Linear Integrate and Fire (GLIF) models in a custom Python simulator) formats. These have both been converted to NeuroML/LEMS formats on http://www.opensourcebrain.org/projects/alleninstituteneuroml. Each cell shown in Figure 3A has a unique complement of ionic conductance densities tuned to the original cell’s electrophysiological recordings.

Neuronal reconstructions from http://neuromorpho.org/ (Ascoli et al., 2007) are available in standardized SWC format (Cannon et al., 1998) and these files, containing information on the 3D locations, radii, connectivity, and type of the points in the reconstruction, can be visualized directly on OSB by placing them into a GitHub repository (see http://www.opensourcebrain.org/projects/neuromorpho). These files can be converted to NeuroML, to build them into spiking neuron models with active conductances, by using the application at https://github.com/pgleeson/Cvapp-NeuroMorpho.org or by loading the SWC into neuroConstruct (Gleeson et al., 2007), editing the cells and exporting to NeuroML. The Janelia MouseLight project (Economo et al., 2016) provides SWC versions of their neuronal reconstructions, in addition to a proprietary JSON file format with extra metadata. The OSB project for this (http://www.opensourcebrain.org/projects/mouselightshowcase) provides scripts for converting the JSON files to NeuroML while retaining the metadata.

For both the NeuroMorpho.Org and MouseLight repositories, a small set of example cell files have been converted to NeuroML and added for visualization on OSB, and instructions/scripts are included for converting any other cells from those resources to NeuroML.

#### Testing and model validation

Model development requires systematic testing of the code base. In software engineering, it is standard practice to run automated tests whenever a change is made to the code and it is committed to a repository (“continuous integration”). To facilitate this on OSB we have developed the OSB Model Validation framework (OMV, https://github.com/OpenSourceBrain/osb-model-validation), which allows test configuration files to be added to the repository for an OSB project. Expected behaviors (e.g., spike times, resting membrane potential), as well as consistency checks for parameters such as total membrane area and temperature are described in short Model Emergent Properties (mep) files. Then a number of OSB Model Test (omt) files are written for each of the simulator configurations (engines) which should produce that behavior, specifying the allowed tolerance. Simulator entries in the last 3 columns of Table S2 are generally associated with individual passing tests (one omt file per simulator) linked by producing the same behavior as described in a shared mep file.

OMV can be installed locally and run at command line (see Table S3) and is also used on the continuous integration (Duvall et al., 2007) service Travis-CI (https://travis-ci.org). Each time there is a commit to a GitHub repository for a model, OMV is launched during the test on Travis-CI, the appropriate simulators are installed and all of the OMV tests in that repository are set running. This ensures the full sequence of simulator installation, model execution, and validation is run on every change to the model code, quickly highlighting any deviations from expected behavior (or simulator compatibility issues).

#### Inhibition stabilized network models

The model shown in Figure 7B is a reimplementation of Sadeh et al. (2017) in PyNN (original spiking model developed in NEST). The networks consisted of 800 excitatory (E) and 200 inhibitory (I) neurons. E and I neurons had the same properties and were modeled using an exponential integrate-and-fire model (Brette and Gerstner, 2005), without adaptation. All neurons received a baseline input modeled as an independent homogeneous Poisson process with constant firing rate (9600 Hz). Recurrent connections were drawn from a binomial distribution, with average probability of 15% for E→{E,I} and 100% for I→{E,I} connections, reflecting the denser connectivity of inhibitory neurons as reported in the cortex (Hofer et al., 2011). The average connection strength of E→{E,I} and I→{E,I} connections, parameterized by the peak synaptic conductance, were set to 0.1 nS and 0.2 nS, and the reversal potentials for excitation and inhibition were 0 mV and −75 mV, respectively. The connection strength for background input was 0.1 nS. To detect ISN properties of the network, the baseline activity of the network was perturbed by changing the input to a fraction of inhibitory neurons. The perturbation was performed by *reducing* the baseline input to perturbed inhibitory neurons by 400 Hz (i.e., by ∼4%) and was repeated for multiple trials to obtain average firing rates before and after perturbation. For further details on the model, including detailed properties of neurons and connectivity, see Sadeh et al., 2017. Code is available in the *PyNN* subfolder of https://github.com/OpenSourceBrain/SadehEtAl2017-InhibitionStabilizedNetworks. The model was exported to NeuroML from PyNN to generate the 3D view on OSB.

In order to create more realistic conductance-based, ISN models, the exponential integrate-and-fire neuron model in Figure 7B was replaced with different neuron types in Figure 7C, while keeping other properties of the network connectivity and stimulation protocol similar. To this end, Hodgkin-Huxley type point neurons with voltage-gated membrane conductances from Pospischil et al. (2008) were scaled to match the firing behavior of layer 2/3 spiny (E, red) and aspiny (I, blue) neurons from the Allen Cell Types Database. Scripts were created to download electrophysiological data from mouse visual cortex neurons from the Allen Cell Types Database (subfolder *CellTypesDatabase/data* on https://github.com/OpenSourceBrain/AllenInstituteNeuroML). From membrane potential traces of cells receiving 1 s current pulse stimuli (examples shown in Figures S3A and S3C), information was extracted on input stimuli information, spike times, spike height, subthreshold steady states and these values were used to retune the model of Pospischil et al. (2008) (subfolder *CellTypesDatabase/tune* in above repository). This single compartment model featured a fast Na^+^, delayed rectifier and M-type K^+^, L-type Ca^2+^ and leak conductances. Conductance densities of active and leak channels, reversal potentials of ions (over a restricted, physiologically plausible range), cell capacitance and voltage dependence of the Na^+^ conductance were used as free parameters in the model tuning, for which the package Neurotune (https://github.com/NeuralEnsemble/neurotune) was used. Two cells were selected from Allen Cell Types Database for the E and I neurons in our network model. One cell from layer 2/3 which exhibited spiny dendrites and showed positive expression for the gene Slc17a6 (vesicular glutamate transporter) was chosen for the E cell (http://celltypes.brain-map.org/mouse/experiment/electrophysiology/477127614) and one with aspiny dendrites and positive expression for somatostatin was chosen for the I neuron (http://celltypes.brain-map.org/mouse/experiment/electrophysiology/476686112).

Figures S3A–S3D show the original electrophysiological data from these cells along with the equivalent behavior for the tuned cell models.

To address how ISN properties can be detected in more realistic scenarios involving detailed neurons equipped with nonlinear dendritic integration we built a hybrid model (Figure 7D). Here, the previous network (Figure 7C) was augmented by replacing 10 of the 800 E cells with the morphologically detailed cell model of Smith et al. (2013), while the axonal connections were omitted for these cells. Figure S3E shows the structure of the network and inputs/connections present, and Figure S4A shows connections generated onto the dendrites of one of the detailed cells. To detect the ISN signature in the detailed cells, the somata of two morphologically complex cells were voltage clamped at −80mV and 0 mV, revealing the E and I postsynaptic currents respectively (Figure 7D, inset). This process was repeated for 10 independent realizations of the model, to obtain a reliable estimate of the inputs. In order to generate multiple instances of a network model which had varying numbers of point neurons/detailed cells and configurable inputs, a script was created in Python (ISN.py, available in https://github.com/OpenSourceBrain/MultiscaleISN) which used libNeuroML to position the cells in 3D space, connect the populations and apply external spiking inputs. Both Figures 7C and 7D were generated from this script.

### Data and Software Availability

All of the code for the OSB platform and the models presented here is open source. The Ruby on Rails based frontend for OSB is at https://github.com/OpenSourceBrain/redmine (GNU General Public License v2) and the Geppetto repositories are listed at https://github.com/openworm/org.geppetto (MIT License). The repositories for the majority of models in Figure 2A can be found at https://github.com/OpenSourceBrain (generally released under MIT License, with information on how to cite the models when reused in CITATION files). Direct links to individual OSB projects can be found in Table S1 and also at http://www.opensourcebrain.org/projects.

## Supplemental Information

Document S1. Figures S1–S4 and Tables S1–S4

Document S2. Article plus Supplemental Information
