# Supplementary material for: Open Source Brain: A Collaborative Resource for Visualizing, Analyzing, Simulating, and Developing Standardized Models of Neurons and Circuits
Source: Neuron. 2019 Aug 7;103(3):395–411.e5. doi: 10.1016/j.neuron.2019.05.019 (PMC6693896; doi:10.1016/j.neuron.2019.05.019)
Supplement: Document S1. Figures S1–S4 and Tables S1–S4 [file mmc1.pdf]

Neuron, Volume *103*

Supplemental Information

Open Source Brain: A Collaborative Resource for Visualizing, Analyzing, Simulating, and Developing Standardized Models of Neurons and Circuits

Padraig Gleeson, Matteo Cantarelli, Boris Marin, Adrian Quintana, Matt Earnshaw, Sadra Sadeh, Eugenio Piasini, Justas Birgiolas, Robert C. Cannon, N. Alex Cayco-Gajic, Sharon Crook, Andrew P. Davison, Salvador Dura-Bernal, András Ecker, Michael L. Hines, Giovanni Idili, Frederic Lanore, Stephen D. Larson, William W. Lytton, Amitava Majumdar, Robert A. McDougal, Subhashini Sivagnanam, Sergio Solinas, Rokas Stanislovas, Sacha J. van Albada, Werner van Geit, and R. Angus Silver

Reference	Physiological properties	Model implementation	Link
Allen Institute Cell Types DB (Hawrylycz et al., 2016)	Morphologically detailed and point neuron models based on electrophysiological recordings from visual cortex neurons	Multicompartmental and Generalized Linear Integrate and Fire (GLIF) neuron models	URL
Brunel (2000)	Spiking network illustrating balance between excitation and inhibition	Integrate and Fire (I&F) neurons, abstract network; implementations in PyNN, NeuroML and NEST	URL
Hay et al. (2011)	Layer 5 pyramidal cell model constrained by somatic and dendritic recordings	Detailed neuronal morphology, non uniform channel distributions	URL
Izhikevich (2003)	Spiking neuron model reproducing wide range of neuronal activity	2 variable point neuron model	URL
Markram et al. (2015)	Cell models from Neocortical Microcircuit of Blue Brain Project	Multicompartmental cell models of multiple cortical classes each with unique complement of active conductances	URL
Pospischil et al. (2008)	HH based model for different classes of cortical and thalamic neurons	Single compartment model with 5 ion channels reproducing a range of neuronal spiking behaviors	URL
Potjans and Diesmann (2014)	Microcircuit model of sensory cortex with 8 populations across 4 layers	Current based I&F neurons; implementations in PyNN, NeuroML and NEST	URL
Dura-Bernal et al. (2017)	Model of mouse primary motor cortex (M1)	Point neurons connected in columnar network based on realistic connection density distributions	URL
Sadeh et al. (2017)	Point neuron model of Inhibition Stabilized Network	Current based I&F neurons; implementations in native NEST and PyNN	URL
Smith et al. (2013)	Layer 2/3 cell model used to investigate dendritic spikes	Multicompartmental cell model with AMPA-R/NMDA-R mediated synaptic inputs	URL
Traub et al. (2005)	Single column network model containing 14 cell populations from cortex and thalamus	Semi-realistic neuronal morphologies; cell region specific distributions of active conductances; chemical & electrical synapses	URL
Cayco-Gajic et al. (2017)	Cerebellar granule cell layer network	I&F based model for granule cell and anatomically constrained connectivity	URL
Maex and De Schutter (1998)	Cerebellar granule cell layer network	Conductance based point neuron models for granule and Golgi cells	URL
Solinas et al. (2007)	Cerebellar Golgi cell model	Conductance based model with abstract morphology	URL
Vervaeke et al. (2010)	Electrically connected cerebellar Golgi cell network model	Detailed Golgi cell model; realistic cell density & gap junction connectivity properties	URL
Bezaire et al. (2016)	Full scale network model of CA1 region of hippocampus	Detailed and abstract models of 10 cell types, realistic firing properties and connectivity parameters	URL
Ferguson et al. (2013)	Parvalbumin-positive interneuron from CA1	Model is customized form of Izhikevich cell model	URL
Migliore et al. (2005)	Pyramidal cell from CA1 region of hippocampus	Multicompartmental cell model as used in a number of studies by Migliore and colleagues	URL
Pinsky and Rinzel (1994)	Simplified model of CA3 pyramidal cell	2 compartment model; can be simulated as a single set of ODEs	URL
Wang and Buzsáki (1996)	Hippocampal interneuronal network model exhibiting gamma oscillations	Conductance based point neuron model used in 100 cell all-to-all network model	URL
Migliore et al. (2014)	Large scale olfactory bulb network	600 unique multicompartmental mitral cells models along with simplified granule cells	URL
Boyle and Cohen (2008)	Model of body wall muscle from *C. elegans*	Point neuron model with 3 active conductances and internal Ca^{2+} buffer	URL
FitzHugh (1961)	Simplified form of Hodgkin Huxley model	2 variable cell model	URL
Hodgkin and Huxley (1952)	Classic investigation of the ionic basis of the action potential	HH model cell with current and voltage clamp inputs; model can be explored with interactive tutorial	URL
Prinz et al. (2004)	Pyloric network of the lobster stomatogastric ganglion system	3 conductance based point neuron models connected via analogue (graded) synapses	URL
NeuroMorpho.Org	Digitally reconstructed neurons across multiple species and brain regions	All cells from NeuroMorpho.Org can be downloaded in NeuroML2; representative examples on OSB	URL
Janelia MouseLight	Reconstructed neurons with axons projecting across the whole mouse brain	Cells downloaded from MouseLight website can be converted to NeuroML2; examples on OSB	URL

Supplementary Table 1. Models on Open Source Brain. Related to Figure 2A. A brief description of the properties of neuronal systems being modeled and the key model implementation features. Colors in first column correspond to the color code for brain regions in **Figure 2A**. Links are provided for accessing the model project pages at http://www.opensourcebrain.org.

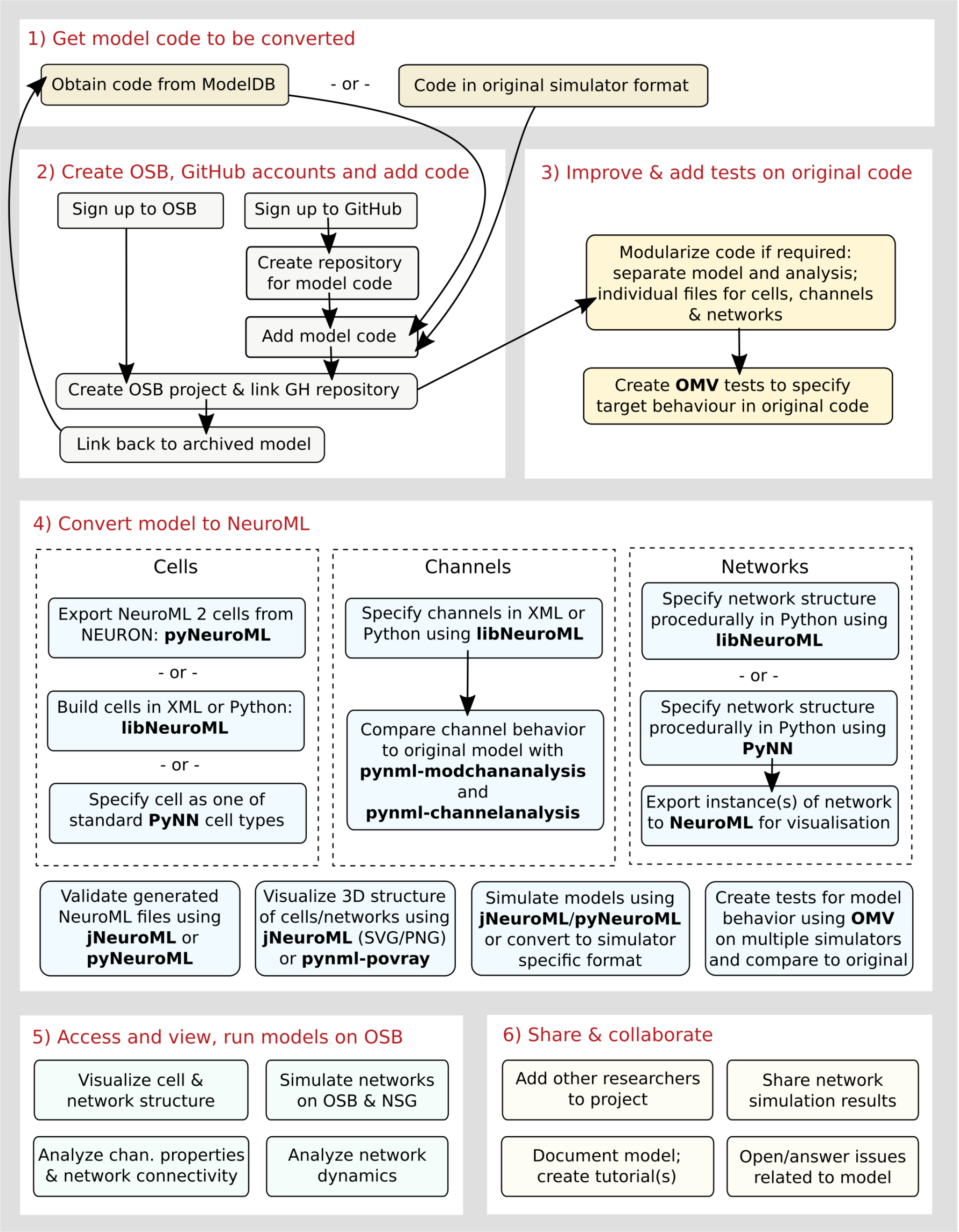

Supplementary Figure 1. Procedures and tools to convert models from native formats into NeuroML and PyNN on Open Source Brain. Related to Figure 1A. Outline of the steps required for converting a model from its native language into NeuroML/PyNN format in order to utilize the visualization and simulation functionality on Open Source Brain (OSB) and for utilizing automated validation and other collaborative development tools. 1) The model code in the original format is obtained from the model developer(s) or from repositories such as ModelDB (McDougal et al., 2017). 2) User accounts are created on OSB and on GitHub. A new repository is created on GitHub and linked to an OSB project for the model. 3) Optionally, the code should be cleaned up, further documented, and made more modular, with all changes recorded in the version control system on GitHub. OSB Model Validation framework (OMV) tests can be added to the scripts which record the behavior of cell(s) in the original model and which the converted model needs to replicate. 4) The numerous tools available to build, export (e.g. from NEURON), validate, visualize and simulate the NeuroML/PyNN version of the model elements on a user's local machine. As the structure of the model is replicated in NeuroML/PyNN, its properties can be tested against the behavior of the original model using OMV. 5) Once valid model elements are uploaded to GitHub, these can be visualized, analyzed and simulated on OSB. 6) Options available to make the model accessible, to get input from other users on the model and to share development responsibilities.

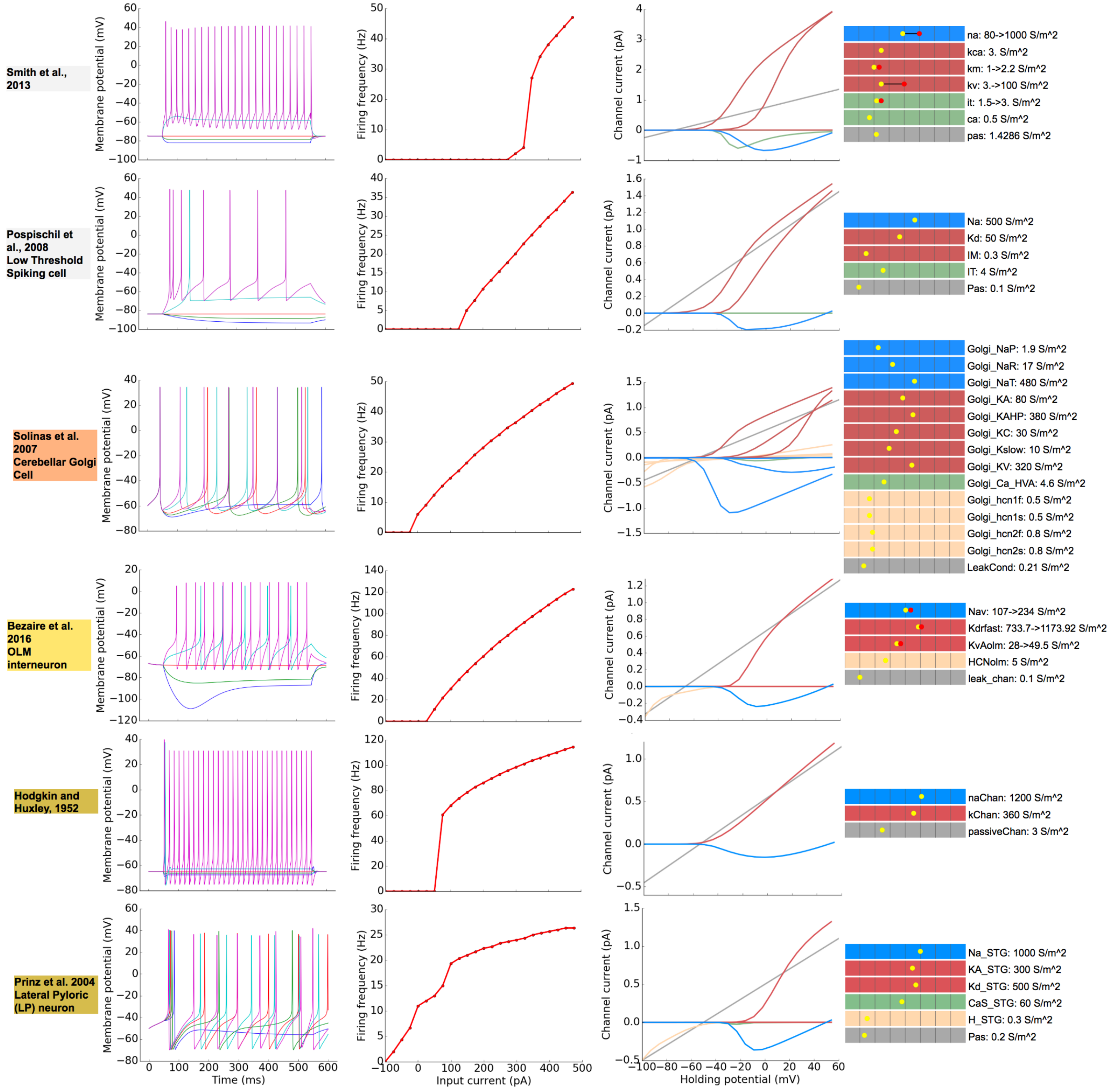

Supplementary Figure 2. **Comparison of the properties of cells and membrane conductances on Open Source Brain. Related to Figure 2A.** Once neuronal models have been converted to NeuroML format their physiological properties can be readily compared, using same scripts to simulate and analyze the properties of different models (e.g. in Python using libNeuroML (STAR Methods)). From left to right the columns show: the time course of the somatic membrane potential for 5 levels of somatic current injection (column 1); firing frequency as a function of current injected (column 2); currents through each conductance present in the soma when the cell is clamped at various holding potentials for the same conductance value (for the default minimal conductance of 10 pS in this case; column 3, colors correspond to colored scale in the next column); densities of the ionic conductances (column 4; logarithmic color scale shows densities of conductances grouped according to the ions transmitted: blue - Na^+, red - K^+, green - Ca^{2+}, light brown - non selective HCN channels, grey - passive conductances; yellow dot is value if conductance is uniform, yellow is minimum and red maximum if nonuniform).

Main publication	Valid NeuroML	PyNN	jNeuroML	jNeuroML to NEURON	Other tests
Allen Institute Cell Types DB (Hawrylycz et al. (2016)	✓		✓	✓	NEURON; NeuroML→ NetPyNE
Brunel (2000)	✓	Brian; NEURON; NEST; NeuroML	✓	✓	PyNEST; PyNN→ NeuroML
Hay et al. (2011)	✓		✓	✓	NeuroML→ NetPyNE
Izhikevich (2003)	✓	Brian; NEURON; NEST	✓	✓	Octave; PyNEURON
Markram et al. (2015)	✓		✓	✓	NEURON; NeuroML→ NetPyNE
Pospischil et al. (2008)	✓		✓	✓	NEURON; NeuroML→ Moose; NeuroML→ NetPyNE
Potjans and Diesmann (2014)	✓	NEST			NEST; PyNEST
Dura-Bernal et al. (2017)	✓		✓	✓	NetPyNE; NeuroML→ NetPyNE
Sadeh et al. (2017)		NEST			PyNEST
Smith et al. (2013)	✓		✓	✓	PyNEURON; NeuroML→ NetPyNE
Traub et al. (2005)	✓		✓	✓	neuroConstruct; NeuroML→ NetPyNE
Cayco-Gajic et al. (2017)			✓		
Maex and De Schutter (1998)	✓		✓	✓	NeuroML→ NetPyNE
Solinas et al. (2007)	✓		✓	✓	NEURON
Vervaeke et al. (2010)	✓			✓	
Bezaire et al. (2016)	✓		✓	✓	NEURON; NeuroML→ NetPyNE
Ferguson et al. (2013)		NeuroML→ PyNN→ NEURON	✓	✓	Brian; Brian2; NeuroML→ Brian2
Migliore et al. (2005)	✓		✓	✓	
Pinsky and Rinzel (1994)			✓		
Wang and Buzsaki (1996)			✓	✓	Brian; NEURON; NeuroML→ NetPyNE
Migliore et al. (2014)	✓			✓	PyNEURON; NeuroML→ NetPyNE
Boyle and Cohen (2008)	✓	NeuroML→ PyNN→ NEURON	✓	✓	Octave; NeuroML→ NetPyNE
FitzHugh (1961)	✓		✓	✓	NeuroML→ Brian; NeuroML→ Brian2
Hodgkin and Huxley (1952)	✓	NeuroML→ PyNN→ NEURON	✓	✓	NeuroML→ Brian2; NeuroML→ Moose; NeuroML→ NetPyNE
Prinz et al. (2004)	✓		✓	✓	NeuroML→ NetPyNE
NeuroMorpho.Org	✓				
Janelia MouseLight	✓				

Supplementary Table 2. Automated testing of models on Open Source Brain. Related to Figure 2A. The models shown in **Figure 2A** have been tested for the validity of their NeuroML implementations and against expected behavior across a number of simulators. Colors in the first column are those used in **Figure 2A**. Tests include: (column 2) validity of the NeuroML files in the repository; (column 3) behavior of the PyNN scripts on different simulator backends (e.g. Brunel (2000) can be run in Brian, NEURON and NEST and exported to NeuroML), or conversion of the NeuroML files to PyNN scripts and execution on a simulator (e.g. NEURON for Ferguson et al., 2013); (column 4) execution of the NeuroML models using jNeuroML's native simulator (point neuron models only); (column 5) execution of the NeuroML models in NEURON, translated using jNeuroML; (column 6) execution of original simulator code in the repository (e.g. NEURON, Brian or Octave scripts taken from ModelDB as used in original publications) or other simulator code (NetPyNE, Brian 2) produced by converting the NeuroML representation using jNeuroML. Note, some models are not fully valid NeuroML because they have custom LEMS components (e.g. Ferguson et al., 2013 uses a custom form of the Izhikevich model). The current testing status of these and all other projects on OSB can be found here: http://www.opensourcebrain.org/status.

Steps	Instructions
1) Install Docker	See https://www.docker.com; available for Windows, Mac OS or Linux.
2) Pull an image with all simulators and stable projects	`docker pull opensourcebrain/simulation:osb_models-v0.8.5`
3) Start a container with the image	`docker run -it opensourcebrain/simulation:osb_models-v0.8.5 /bin/bash`
4) Inside container, go to folder with models and run the tests using OMV	`cd coreprojects`<br>`omv all`

Supplementary Table 3. Obtaining all core OSB models and testing against supported simulators. Related to STAR Methods. A Docker image (preconfigured computational environment with files, libraries, etc.; https://www.docker.com) has been created containing all of the simulators currently supported by OSB, along with all of the models presented in **Figure 2** and **Supp. Table 1**. Once Docker is installed (1), the image can be pulled from the central Docker registry (2). A container using this image can be set running (3), and OMV (STAR Methods) used to run tests on each of the models (4), in all the simulators it supports. The commands in blue above need to be typed at the command line. Alternatively, the Kitematic application bundled with Docker can be used for steps 2) and 3), the image opensourcebrain/simulation:osb_models-v0.8.5 searched for and a new container created through the interface. The commands in (4) can then be entered into a command line terminal connected to this.

Software package	Version	Website
Brian	v1.4.4	http://briansimulator.org
Brian2	v2.2.1	http://briansimulator.org
MOOSE	v3.2-git	https://moose.ncbs.res.in/
NEST	v2.12.0	http://www.nest-simulator.org
NEURON	v7.4	https://www.neuron.yale.edu
NetPyNE	v0.7.9	http://www.netpyne.org
PyNN	v0.9.2	http://neuralensemble.org/PyNN
PyLEMS	v0.4.9.3	https://github.com/LEMS/pylems
jLEMS	v0.9.9.1	https://github.com/LEMS/jLEMS
jNeuroML	v0.8.4	https://github.com/NeuroML/jNeuroML
pyNeuroML	v0.3.15	https://github.com/NeuroML/pyNeuroML
Python	v2.7.9	https://www.python.org/
Java	v1.7.0	https://www.java.com
Octave	v3.8.2	https://www.gnu.org/software/octave

Supplementary Table 4. Libraries used in Docker container. Related to STAR Methods. The listed versions of the software packages shown for simulators (white rows), NeuroML/PyNN libraries (light grey) and software languages (blue) have been used to test the models presented here. These are also the versions included with the Docker container (**Supp. Table 3**).

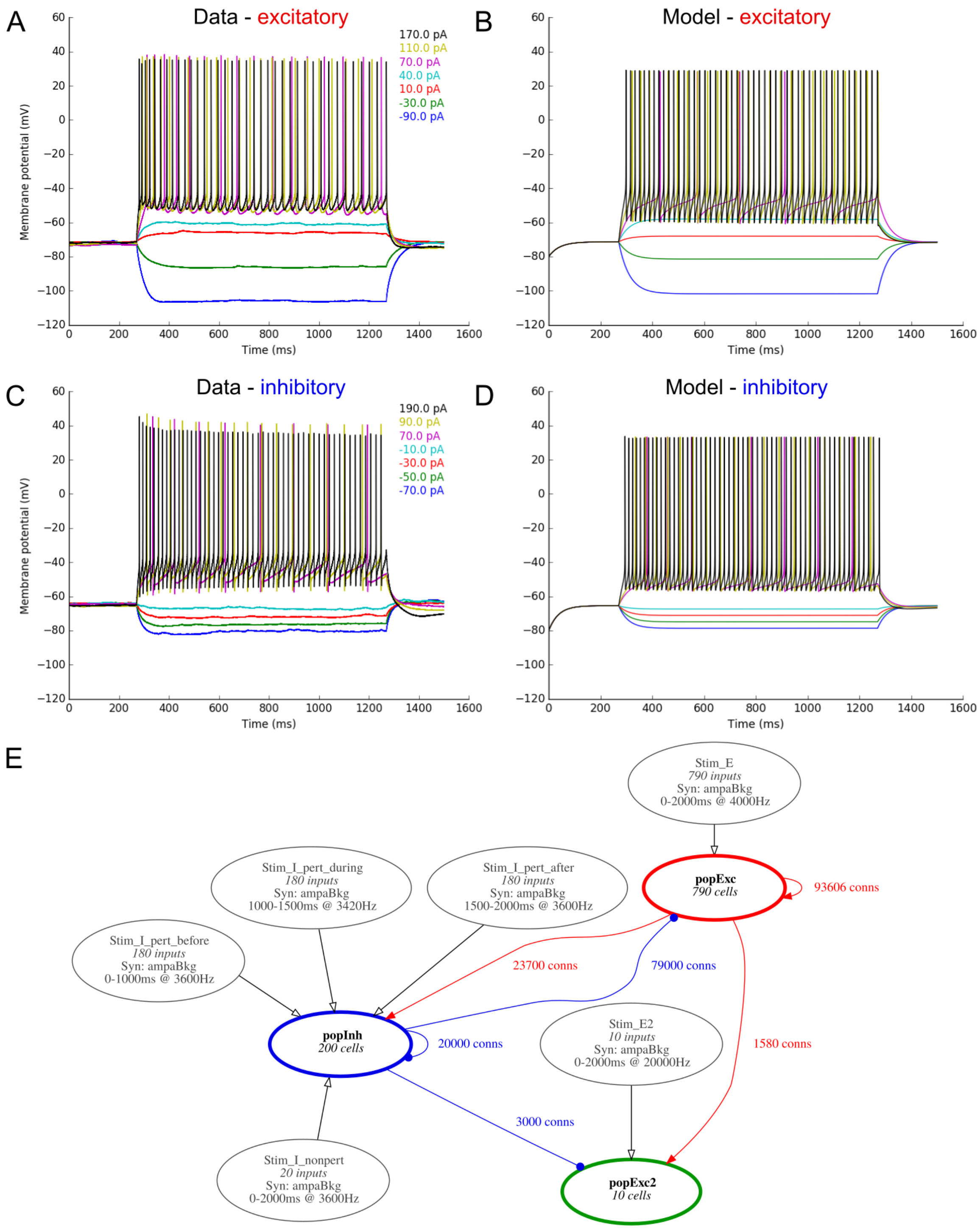

Supplementary Figure 3. Properties of single compartment neurons in hybrid model of inhibition stabilized network, together with connectivity. Related to Figure 7. (A-D) Plots of membrane potential traces from electrophysiological recordings of visual cortex neurons (A, C) and equivalent computational models (specified in NeuroML, executed in NEURON) generated to match the spiking behavior (B, D). Data in A and C taken from Allen Cell Types Database and are recordings of cells in Layer 2/3 of mouse visual (A: dataset 477127614, spiny cell; C: dataset 476686112, aspiny cell). Responses to 1 second current pulses of 7 different amplitudes are shown in each. B and D show the responses to the same inputs (as A and C respectively) of the single compartment computational models based on Pospischil et al., 2008 which have been tuned to reproduce the behavior of the experimental recordings (STAR Methods) and used in the network shown in **Figure 7C**. (E) Schematic diagram showing inputs to and connections between populations of excitatory point neurons (popExc), excitatory detailed neurons (popExc2) and inhibitory point neurons (popInh) for network shown in **Figure 7D**. 10% of the cells in popInh receive a steady input of 3600 Hz for the full 2 seconds, while 90% have a slightly lower input rate from 1-1.5 s. Voltage clamp inputs to 2 of 10 cells in popExc2 not shown for clarity.

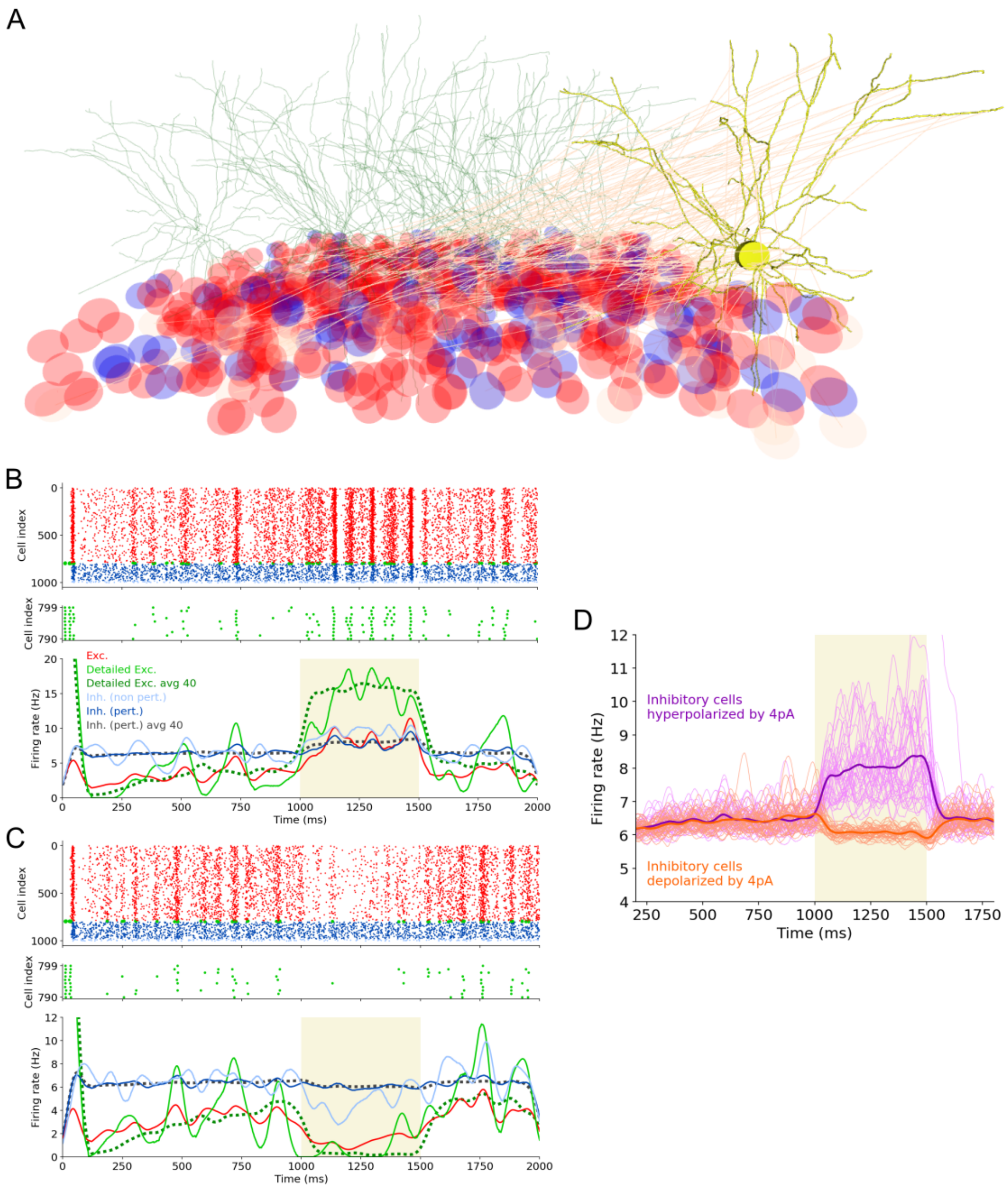

Supplementary Figure 4. Synaptic connectivity of hybrid model and response to the injection of hyperpolarizing and depolarizing currents into interneurons. Related to Figure 7D. (A) Screenshot from OSB of 3D network model (same model as shown in **Figure 7D**) showing connections from excitatory (red) and inhibitory (blue) point neurons onto the dendrites of a single detailed cell (color of selected cell set to yellow and dendrites given larger diameter; all other detailed cells in green). (B) Response of network under similar configuration to **Figure 7D/Supp. Figure 3E**, but with hyperpolarizing current of -4pA (1-1.5 s) applied to 90% of inhibitory cells, as opposed to a decrease in background firing rate. A similar increase in firing rates of all populations is seen (cells 790-799 are detailed cells, green points for these enlarged in upper rasterplot and shown in middle rasterplot; solid green line in lower plot is rate of firing of detailed cells; dashed green line is average of 40 simulations; other traces and calculation of firing rate same as **Figure 7B-C**). Hyperpolarizing current injection was designed to mimic optogenetic inactivation of interneurons with halorhodopsin (cf. Figure 5 of Kato et al., 2017). (C) Response of network to transient application of depolarizing current (4pA from 1-1.5 s). A decrease in firing of all populations is seen. Similar plots as for (B). (D) Plots of rate of firing in the 90% of stimulated interneurons when hyperpolarized (light purple lines: 40 individual simulations; thick dark purple line: average) and depolarized (light orange lines: 40 individual simulations; bold dark orange line: average). This shows the paradoxical effect of ISNs: when the interneurons receive a small positive current input they reduce their firing rates, when they receive a small negative current input they increase their firing rates.
