# Supplementary material for: Open Source Brain: A Collaborative Resource for Visualizing, Analyzing, Simulating, and Developing Standardized Models of Neurons and Circuits
Source: Neuron. 2019 Aug 7;103(3):395–411.e5. doi: 10.1016/j.neuron.2019.05.019 (PMC6693896; doi:10.1016/j.neuron.2019.05.019)
Supplement: Document S2. Article plus Supplemental Information [file mmc5.pdf]

NeuroResource

Neuron

Open Source Brain: A Collaborative Resource for Visualizing, Analyzing, Simulating, and Developing Standardized Models of Neurons and Circuits

Graphical Abstract

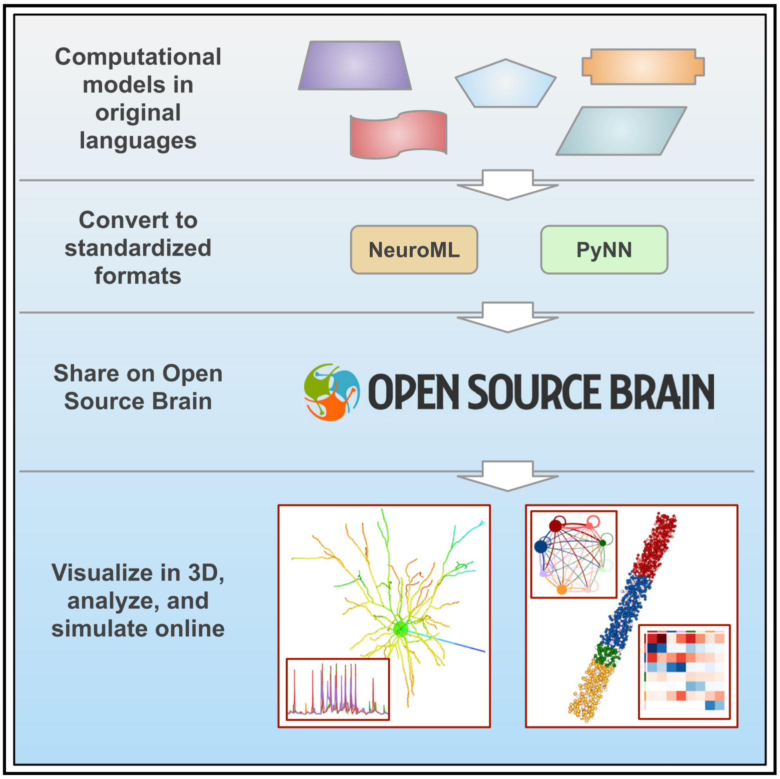

Authors

Padraig Gleeson, Matteo Cantarelli, Boris Marin, ..., Sacha J. van Albada, Werner van Geit, R. Angus Silver

Correspondence

a.silver@ucl.ac.uk

In Brief

Open Source Brain is an online resource of neuronal and circuit models that enables browser-based visualization, analysis, and simulation. Gleeson et al. describe how the resource and tools for collaborative model development provide accessible, up-to-date models from different brain regions.

Highlights

- Open Source Brain: an online resource of standardized models of neurons and circuits
- Automated 3D visualization, analysis, and simulation of models through the browser
- Open source infrastructure and tools for collaborative model development and testing
- Accessible, transparent, up-to-date models from different brain regions

Gleeson et al., 2019, Neuron *103*, 395–411
August 7, 2019 © 2019 The Authors. Published by Elsevier Inc.
https://doi.org/10.1016/j.neuron.2019.05.019

CellPress

CellPress

Open Source Brain: A Collaborative Resource for Visualizing, Analyzing, Simulating, and Developing Standardized Models of Neurons and Circuits

Padraig Gleeson,[1] Matteo Cantarelli,[1,2] Boris Marin,[1,3] Adrian Quintana,[1] Matt Earnshaw,[1] Sadra Sadeh,[1] Eugenio Piasini,[1,4] Justas Birgiolas,[5] Robert C. Cannon,[6] N. Alex Cayco-Gajic,[1] Sharon Crook,[5,7] Andrew P. Davison,[8] Salvador Dura-Bernal,[9] András Ecker,[1,10] Michael L. Hines,[11] Giovanni Idili,[2] Frederic Lanore,[1] Stephen D. Larson,[12] William W. Lytton,[9] Amitava Majumdar,[13] Robert A. McDougal,[11,14] Subhashini Sivagnanam,[13] Sergio Solinas,[15,16] Rokas Stanislovas,[1] Sacha J. van Albada,[17] Werner van Geit,[10] and R. Angus Silver[1,18,*]
[1]Department of Neuroscience, Physiology and Pharmacology, University College London, London, UK
[2]MetaCell Limited, Oxford, UK
[3]Centro de Matemática, Computação e Cognição, Universidade Federal do ABC, Santo André, Brazil
[4]Computational Neuroscience Initiative and Department of Physics and Astronomy, University of Pennsylvania, Philadelphia, PA, USA
[5]School of Life Sciences, Arizona State University, Tempe, AZ, USA
[6]Annotate Software Limited, Edinburgh, UK
[7]School of Mathematical and Statistical Sciences, Arizona State University, Tempe, AZ, USA
[8]Unité de Neuroscience, Information et Complexité, Centre National de la Recherche Scientifique, Paris, France
[9]SUNY Downstate Medical Center and Kings County Hospital, Brooklyn, NY, USA
[10]Blue Brain Project, Ecole Polytechnique Federale de Lausanne (EPFL), Lausanne, Switzerland
[11]Department of Neuroscience, Yale School of Medicine, New Haven, CT, USA
[12]OpenWorm Foundation, Boston, MA, USA
[13]University of California, San Diego, San Diego, CA, USA
[14]Center for Medical Informatics, Yale University, New Haven, CT, USA
[15]Department of Biomedical Science, University of Sassari, Sassari, Italy
[16]Institute of Neuroinformatics, University of Zurich and ETH Zurich, Zurich, Switzerland
[17]Institute of Neuroscience and Medicine (INM-6), Institute for Advanced Simulation (IAS-6) and JARA-Institut Brain Structure-Function Relationships (INM-10), Jülich Research Centre, Jülich, Germany
[18]Lead Contact
*Correspondence: a.silver@ucl.ac.uk
https://doi.org/10.1016/j.neuron.2019.05.019

SUMMARY

Computational models are powerful tools for exploring the properties of complex biological systems. In neuroscience, data-driven models of neural circuits that span multiple scales are increasingly being used to understand brain function in health and disease. But their adoption and reuse has been limited by the specialist knowledge required to evaluate and use them. To address this, we have developed Open Source Brain, a platform for sharing, viewing, analyzing, and simulating standardized models from different brain regions and species. Model structure and parameters can be automatically visualized and their dynamical properties explored through browser-based simulations. Infrastructure and tools for collaborative interaction, development, and testing are also provided. We demonstrate how existing components can be reused by constructing new models of inhibition-stabilized cortical networks that match recent experimental results. These features of Open Source Brain improve the accessibility, transparency, and reproducibility of models and facilitate their reuse by the wider community.

INTRODUCTION

Computational modeling is a powerful approach for investigating and understanding information processing in neural systems (Dayan and Abbott, 2001; Herz et al., 2006; Sejnowski et al., 1988). Such models have played a central role in elucidating the mechanisms underlying synaptic transmission (Del Castillo and Katz, 1954), the action potential (Hodgkin and Huxley, 1952), dendritic integration (Rall, 1962), and, more recently, circuit function (Bezaire et al., 2016; Billings et al., 2014; Cayco-Gajic et al., 2017; Diesmann et al., 1999; Markram et al., 2015; Potjans and Diesmann, 2014; Sadeh et al., 2017).

Models range widely in their level of biological detail, ranging from reduced "top-down" models that provide insights into high-level dynamical behavior of circuits to biologically detailed "bottom-up" models (Bezaire et al., 2016; Markram et al., 2015; Potjans and Diesmann, 2014; Traub et al., 2005) that enable investigation of the mechanisms underlying circuit function. Biologically detailed circuit models are necessarily complex and typically have a large number of parameters. Experimental

© 2019 The Authors. Published by Elsevier Inc.
This is an open access article under the CC BY license (http://creativecommons.org/licenses/by/4.0/).

measurements from connectomics (Helmstaedter et al., 2013; Kasthuri et al., 2015), functional activity mapping (Ahrens et al., 2013), and multi-cell and automated patch clamping (Annecchino et al., 2017; Guzman et al., 2016), together with datasets from large-scale brain initiatives (Amunts et al., 2016; Hawrylycz et al., 2016; Insel et al., 2013; Kandel et al., 2013), are providing an increasingly wide range of data to constrain such models, thereby improving their accuracy. But confidence in the predictions from biologically detailed models is currently limited by their complexity and their perceived lack of constraint.

Constructing well-constrained models of neurons and circuits from a raw dataset takes a considerable amount of time and skill despite well-established simulation tools (Carnevale and Hines, 2006; Gewaltig and Diesmann, 2007; Goodman and Brette, 2008; Ray and Bhalla, 2008). Once built, the complexity of detailed models makes them difficult to modify for new scientific questions. Moreover, running large-scale circuit models often requires high-performance computing facilities, which may not be accessible to many end users and brings an additional layer of difficulty to setting up simulations and managing the resultant datasets. These technical barriers hinder access to the structural and functional properties of biologically detailed models, limiting scientific scrutiny and adoption of these powerful tools by the wider community.

Ensuring biologically detailed models are robust and error free is challenging given the length and complexity of their software implementations. Common errors include typos in equation definitions and parameter values, unit conversions, inconsistent use of temperature dependencies, and incorrect translation of reconstructed neuronal morphologies. In industry, open source software development is increasingly being used to create well-tested, modular software components and applications, which can be shared publicly using code development and collaboration platforms, such as GitHub (Perkel, 2016). GitHub records changes in the code and allows multiple users to manage and recombine different versions, track issues, and flag stable versions of the code. Errors can be minimized by regularly testing each modular component with automated routines and then assembling them into larger structures. In computational neuroscience, standardized modular frameworks (''model description languages'') have also been developed for specifying the biological components of circuits, such as ionic and synaptic conductances, neuronal morphologies, and synaptic connectivity (Cannon et al., 2014; Davison et al., 2009; Gleeson et al., 2010). These components could be used to build modular models that are easy to configure and test, facilitating their reuse for different scientific questions. However, adoption of strategies currently used in open source software engineering for creating, managing, testing, and validating modular code has been the exception rather than the rule for neural modeling in academia (Eglen et al., 2017).

To address these challenges, we have developed Open Source Brain (OSB) (http://www.opensourcebrain.org), a web-based collaborative resource for viewing, simulating, disseminating, and developing standardized models of neurons and circuits. OSB hosts a range of neuronal and circuit models from multiple brain regions, including the neocortex, cerebellum, and hippocampus. The morphology of modeled neurons, the structure and connectivity of networks, and the values of physiological parameters used can be automatically visualized in graphical form on OSB through a web browser. Moreover, functional properties can be explored by simulating models through the browser without installing programs or writing code. Unlike previous repositories, deep links between OSB and GitHub provide a collaborative resource for developing, refining, and automatically testing models, enabling them to evolve as new information becomes available. OSB functionality has been achieved by combining tools and best practices from the open source software development community, harnessing modern web technologies, and integrating them with standardized modular descriptions of models (Cannon et al., 2014; Davison et al., 2009; Gleeson et al., 2010). Using OSB and associated offline tools, it is possible to combine model components across different levels of biological detail. We illustrate this functionality by constructing models of multiscale inhibition-stabilized cortical networks (ISNs) (Tsodyks et al., 1997) and analyzing their robustness to different levels of biological detail. By making models more accessible and facilitating model development through collaboration, OSB provides an online resource of standardized models that can be critically evaluated and reused by the wider neuroscience community.

RESULTS

The OSB Resource

OSB is an online platform (http://www.opensourcebrain.org) that links open source repositories containing standardized models of neurons and circuits to users and developers. OSB provides powerful tools to visualize, analyze, simulate, develop, and test models through web browsers (Figure 1A). These features were made possible by defining models in the neuroscience model description languages NeuroML (Cannon et al., 2014; Gleeson et al., 2010) and PyNN (Davison et al., 2009). These standardized formats define the properties of models (e.g., biophysical parameters, cell morphology, and connectivity) in a modular, structured way. This enables model files to be automatically read by OSB and the physiological and anatomical details presented through the browser (Figure 1B). They also contain the information required to simulate the model, enabling the functional properties of individual neurons and networks (e.g., membrane potential and firing activity) to be explored. OSB provides access to metadata associated with the models, including the history of their development (provenance), and has links to wikis, allowing users to discuss their performance and any technical issues. The model code is hosted in public software development repositories (e.g., on GitHub), because these provide functionality to track and manage changes to the code. This combination of open source software development infrastructure and model standardization enables OSB to deliver up-to-date versions of models in accessible graphical formats (e.g., 3D views of cells and circuits, tables, and interactive plots) that can be understood and used by the wider neuroscience community.

OSB currently hosts standardized, curated models spanning a wide range of biophysical detail, varying from single cells up to large-scale networks with thousands of neurons (Figure 2A; Table S1). These models cover multiple regions of the brain,

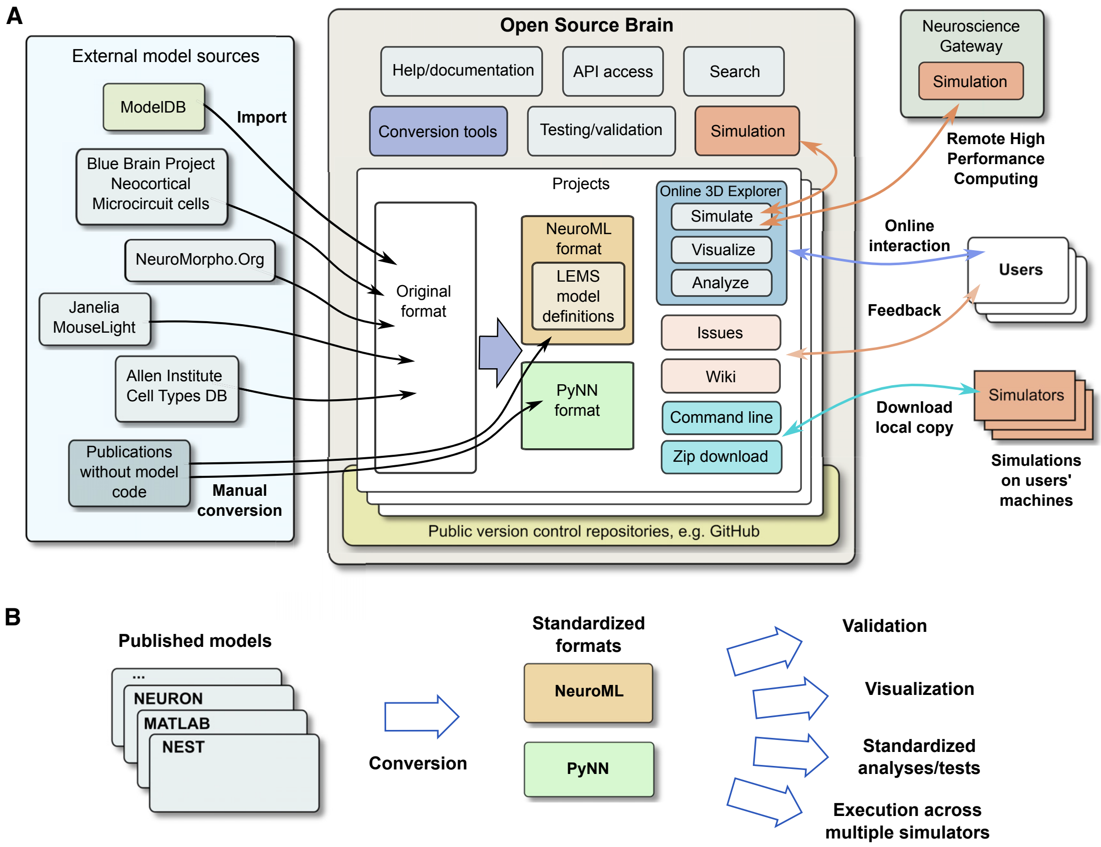

Figure 1. Overview of Open Source Brain

(A) Functionality of Open Source Brain (OSB) and interactions with users and external resources. (Left) Model sources for OSB are shown. Center: OSB resources to facilitate conversion of models in open source repositories to standardized formats; to validate against the standards; to test model code; and to visualize, analyze, and simulate models through a web browser are shown. A search function is provided, together with an application programming interface (API). Right: user interaction with projects can be through the OSB web interface or by command line. Wikis enable feedback, and issues can be opened. Project code can be cloned, forked, or committed using standard open source workflows or downloaded as zipped releases. Simulations can be performed on the OSB server or submitted to the Neuroscience Gateway for execution on their supercomputing facilities. See also Figure S1.

(B) Functionality following the conversion of published models from simulator-specific formats into standardized representations. This includes automated validation, visualization, analysis, and simulations on different platforms, using a variety of generic tools.

including neocortex (Brunel, 2000; Dura-Bernal et al., 2017; Hawrylycz et al., 2016; Hay et al., 2011; Izhikevich, 2003; Markram et al., 2015; Pospischil et al., 2008; Potjans and Diesmann, 2014; Sadeh et al., 2017; Smith et al., 2013; Traub et al., 2005), cerebellum (Cayco-Gajic et al., 2017; Maex and De Schutter, 1998; Solinas et al., 2007; Vervaeke et al., 2010), hippocampus (Bezaire et al., 2016; Ferguson et al., 2013; Migliore et al., 2005; Pinsky and Rinzel, 1994; Wang and Buzsáki, 1996), and olfactory bulb (Migliore et al., 2014). A number of invertebrate models have also been converted (Boyle and Cohen, 2008; Fitzhugh, 1961; Hodgkin and Huxley, 1952; Prinz et al., 2004). At the single-cell level, there are models from the Allen Institute Cell Types Database (Hawrylycz et al., 2016) and the Blue Brain Project (Markram et al., 2015) and reconstructed neuronal morphologies from the NeuroMorpho.Org (Ascoli et al., 2007) and Janelia MouseLight (Economo et al., 2016) databases. In addition to the standardized models presented here, there are a number of other user-contributed models on OSB that are in the process of conversion and curation. This community-driven approach encourages organic growth of models and components on OSB and ensures that the range of models available is determined by the interests of the users of the resource.

User Interaction with OSB

There are a number of different ways users can interact with models on OSB, depending on their goals and level of expertise in computational neuroscience and in programming (Figure 2B). Scientists interested in rapidly learning about the properties of a model used in a scientific study can readily inspect model structure and parameters and replay previously recorded simulations through their browsers without registering as an OSB user. The main OSB projects page (http://www.opensourcebrain.org/projects) provides links to a wide range of models, including all of those presented here. After registering and logging in, users can run and store their own simulations for a more in-depth analysis of the functional properties of the model.

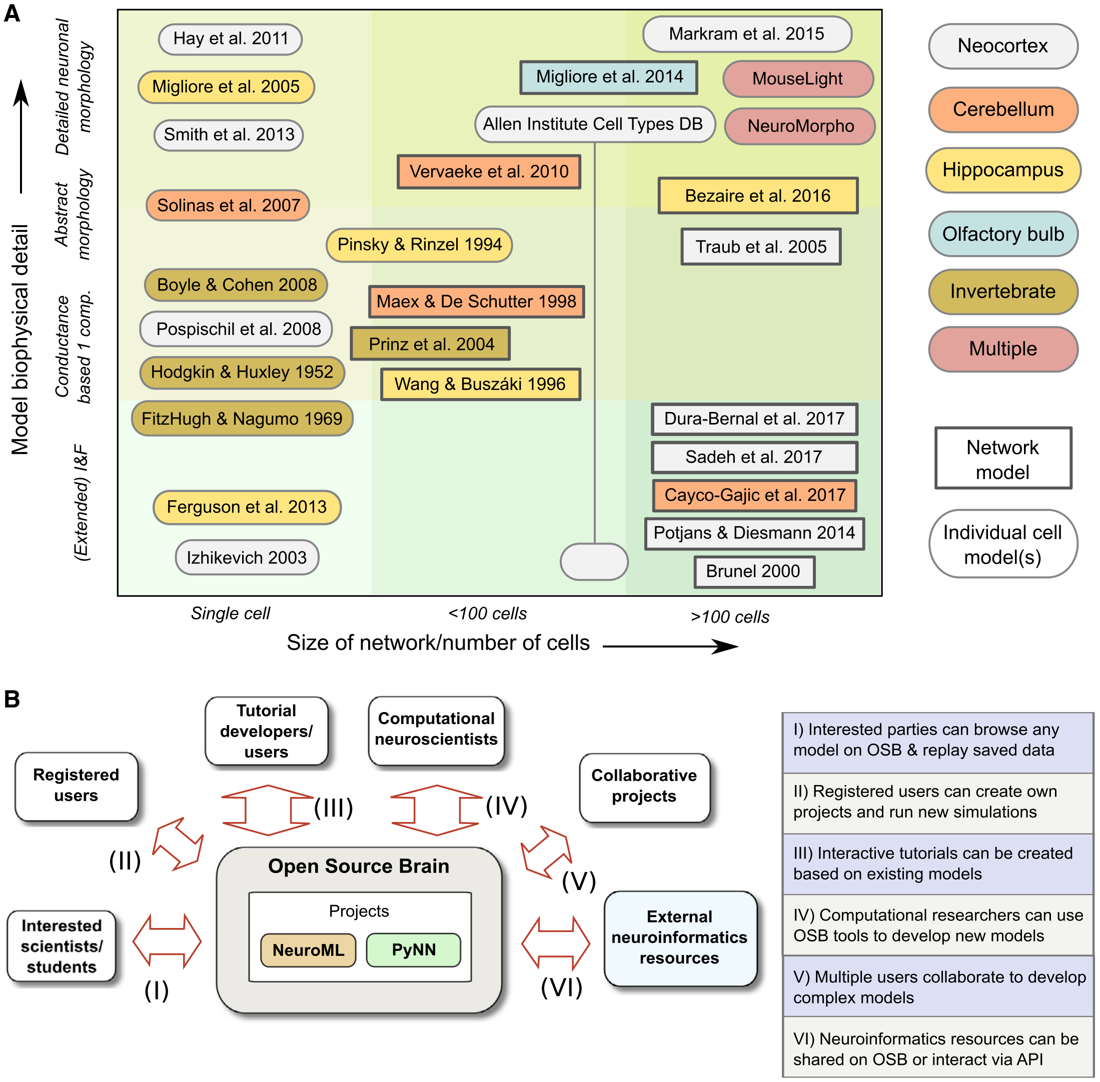

Figure 2. Standardized Multiscale Models on the OSB Platform, Together with User Interactions

(A) NeuroML- and PyNN-based models on OSB, identified by author(s) of the original publications describing the models. The models have different levels of biophysical detail, ranging from simple point neuron models (e.g., integrate and fire [I&F]) to complex multicompartment cell models. Some projects contain single cells, and others contain multiple cell types or network models. Neuronal and circuit models cover a broad range of brain regions and include both vertebrate and invertebrate systems. More details on these models are given in Tables S1 and S2. See also Figure S2.

(B) Usage scenarios for OSB projects containing standardized models in NeuroML or PyNN, depending on users' goals and level of computational expertise.

OSB can also be used to develop online resources for teaching neuroscience to students and researchers. This is facilitated through interactive help functionality and a framework for building tutorials, which can be used to illustrate the biophysical, anatomical, and physiological properties of a model and to help explain different mechanisms, such as the conductances underlying the action potential and synaptic integration.

Scientists wishing to use the infrastructure for model development, collaboration, testing, and dissemination form the core OSB user group (Figures 1A and 2B). Figure S1 provides an overview of the steps required to add a model to OSB and the tools we have developed to facilitate this. Once the model is converted to a standardized format, users are able to use the OSB tools for visualization, automated analysis, and testing to help evaluate the accuracy of their code and minimize errors. This facilitates model refinement by ensuring that the intended behavior is not disrupted after each modification. The OSB infrastructure and associated tools can also support larger scale collaborative projects to build and test more complex models. OSB interacts with other neuroinformatics platforms, enabling content to be shared between resources. For example, there are deep links between OSB and ModelDB (McDougal et al., 2017), an archive of neuronal models in their original published formats. These features of OSB enable neuroscientists from many backgrounds to explore and use biologically detailed models and lower the technical barriers to the more advanced features of the platform.

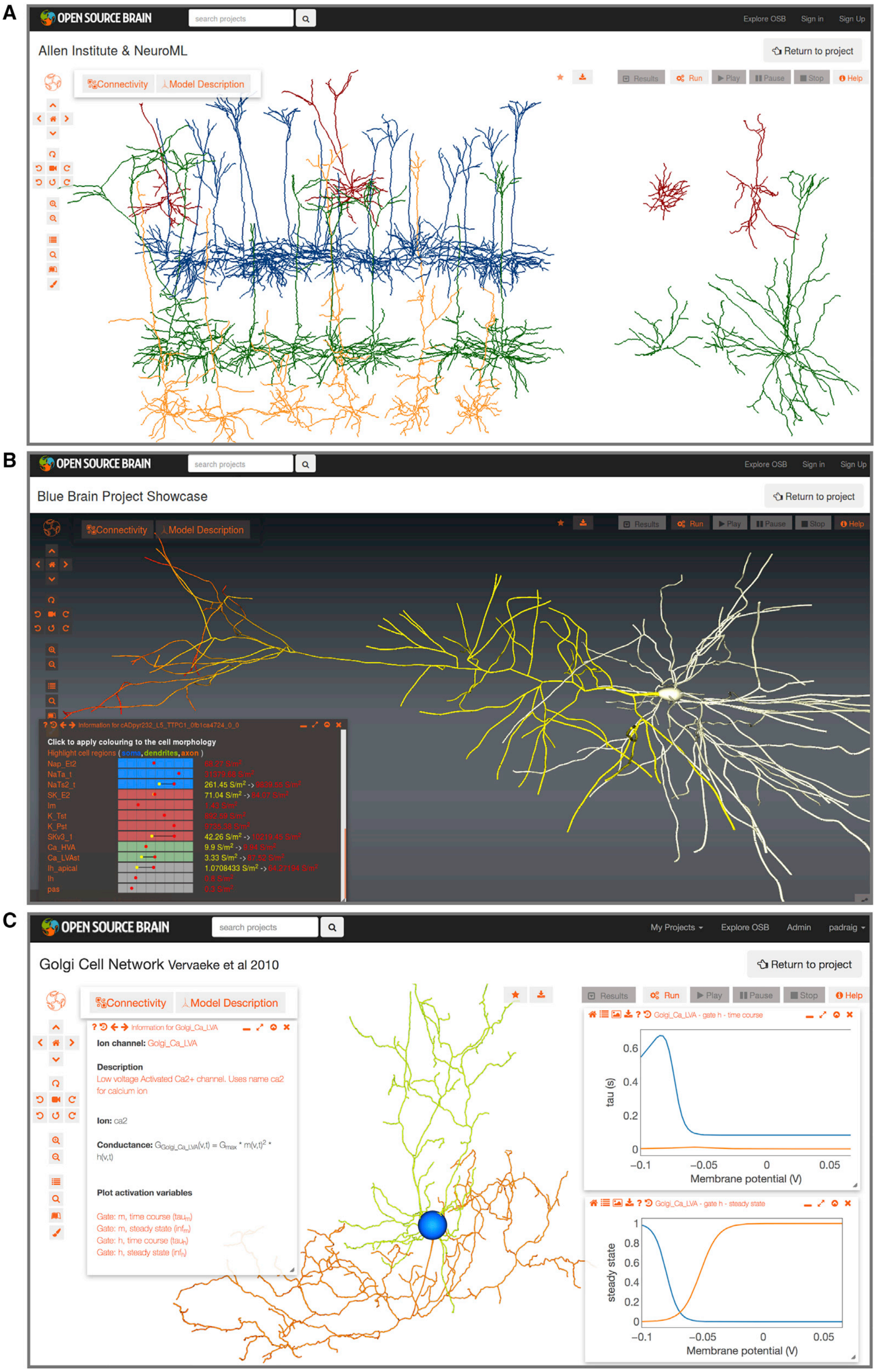

(legend on next page)

Visualization and Analysis of Model Structure

To allow visualization of a model in the web browser, OSB searches for the standardized model descriptions (NeuroML files) in the repositories associated with the OSB project. This information is used to generate a 3D visual representation of the neuronal morphology and/or the circuit structure (Figure 3A). In addition, the spatial distribution of the density of ionic conductances can be viewed either in tabular form or as a pseudocolor density map superimposed on the neuronal morphology (Figure 3B). Because models of ionic conductances are also specified in NeuroML format, the underlying mathematical expressions defining the rates of activation and inactivation can be extracted and plotted (Figure 3C). Thus, the types, distributions, densities, and kinetic properties of the membrane conductances present in the model can be automatically exposed in graphical formats. Other useful information in the NeuroML files, such as authors of the files, references, and links to the original data sources, is also presented through the web interface. This facilitates transparency and enables the history of models and their authors to be recorded (provenance tracking).

The 3D structure of circuit models is often complex, as it can include multiple neuronal layers, a range of cell types distributed at different densities, and extensive synaptic connectivity. OSB facilitates visualization of network structure by automatically generating multiple types of connectivity diagrams. This is possible because NeuroML descriptions of such networks contain structured lists of 3D locations of somata and the subcellular location of chemical and electrical synapses. Figure 4A shows a single-column thalamocortical model consisting of multicompartmental neurons distributed over multiple cortical layers (Traub et al., 2005). The synaptic connectivity of such circuits can be inspected using automatically generated visualizations. A chord diagram (Figure 4B) provides a convenient way to assess the density or sparsity of the synaptic connectivity. In contrast, the connectivity matrix (Figure 4C) provides a more quantitative overview of the synaptic connections, showing the strength of excitatory and inhibitory connections between different cell populations. Lastly, the connectivity plot shown in Figure 4D combines these features in one plot, providing a way to visualize the size of the neuronal populations, the connections between them, and their relative strength. This functionality enables the easy comparison of network connectivity. For example, a cortical network consisting of point neurons (Potjans and Diesmann, 2014) can be analyzed and compared with the previous, more detailed cortical model (Figures 4E–4H). For large-scale networks with a high level of biological detail, such as the recently developed CA1 circuit model (Bezaire et al., 2016), OSB can progressively load parts of the network to speed visualization. For example, visualization of the gross structure of the circuit does not require loading the synaptic connectivity matrix (Figure 4I). However, this can be loaded in the background if required, enabling the properties of the synaptic connectivity to be visualized (Figures 4J–4L). These features substantially extend the options available for exploring model structure when compared to the original versions of these models, because this information was buried deep within the specialized code (Fortran, NEST SLI, and NEURON hoc in Figures 4A–4D, 4E–4H, and 4I–4L, respectively). Videos S1 and S2 illustrate interactive exploration on OSB of the models shown in Figures 3 and 4, respectively.

Functional Properties of Models Revealed through Online Simulation

To make the functional properties of models of neurons and circuits more accessible to the wider community, we have developed browser-based simulations on OSB, which remove the requirement to write code. This functionality is enabled by the simulator-independent nature of the standardized formats of models on OSB. Instructions for simulating the model are fed to the OSB server, where the code for running the simulation is automatically generated and executed (typically using the NEURON simulator; see STAR Methods). Short simulations can be run quickly on computing resources provided by the OSB server, and larger scale computations can be easily submitted for execution through the Neuroscience Gateway at the San Diego Supercomputer Center (Sivagnanam et al., 2013), which provides parallel execution of models across hundreds of processors (Figure 1A; STAR Methods). Upon completion, the data generated are sent back to the browser for visualization (Figures 5 and 6). These features enable exploration of complex circuit models without the requirement for specialist knowledge to setup and run large-scale simulations (Video S3).

Users can also alter values of model parameters through the browser, such as current injection levels and densities of ion conductances (Figure 5). By running multiple simulations, this enables characteristic neuronal properties to be investigated (Figure 6A). More substantial changes to the model, such as adding new conductances or changing the number of cells, currently require offline regeneration of the NeuroML files (Figure S1). Nevertheless, many changes can already be made to investigate cell and network behavior, such as setting a synaptic conductance to zero to remove the connection between two

Figure 3. Visualization of Models through the Browser

(A) Screenshot showing 37 cell models of visual cortex neurons from the Allen Cell Types Database on the OSB website, visualized in 3D through a browser. Spiny (32 on left) and aspiny (5 on right) cells from layers 2/3 (red), 4 (blue), 5 (green), and 6 (orange) are shown.

(B) Layer 5 pyramidal cell from the Blue Brain Project neocortical microcircuit model. Bottom left: an information panel (opened via Model Description button) summarizing the types and densities of ionic conductances on the cell membrane is shown. Individual conductances can be clicked to highlight the regions of the cell where they are present. Cell morphology shows the non-uniform distribution of the hyperpolarization-activated conductance on the apical dendrite (low = yellow near soma; high = red in the distal dendrites).

(C) Cerebellar Golgi cell model from Vervaeke et al. (2010). Cell regions have been highlighted (blue soma, green dendrites, and orange axon). Left information panel for a low-voltage-activated Ca^{2+} conductance (Ca LVA) present on the cell is shown, including conductance expression and gating variables. Right plots show voltage dependences of time constant (top) and steady-state value (bottom) for the activation (orange, m) and inactivation (blue, h) gates. Dendrite and axon diameters are increased for clarity of figure presentation in (A)–(C).

See also Video S1.

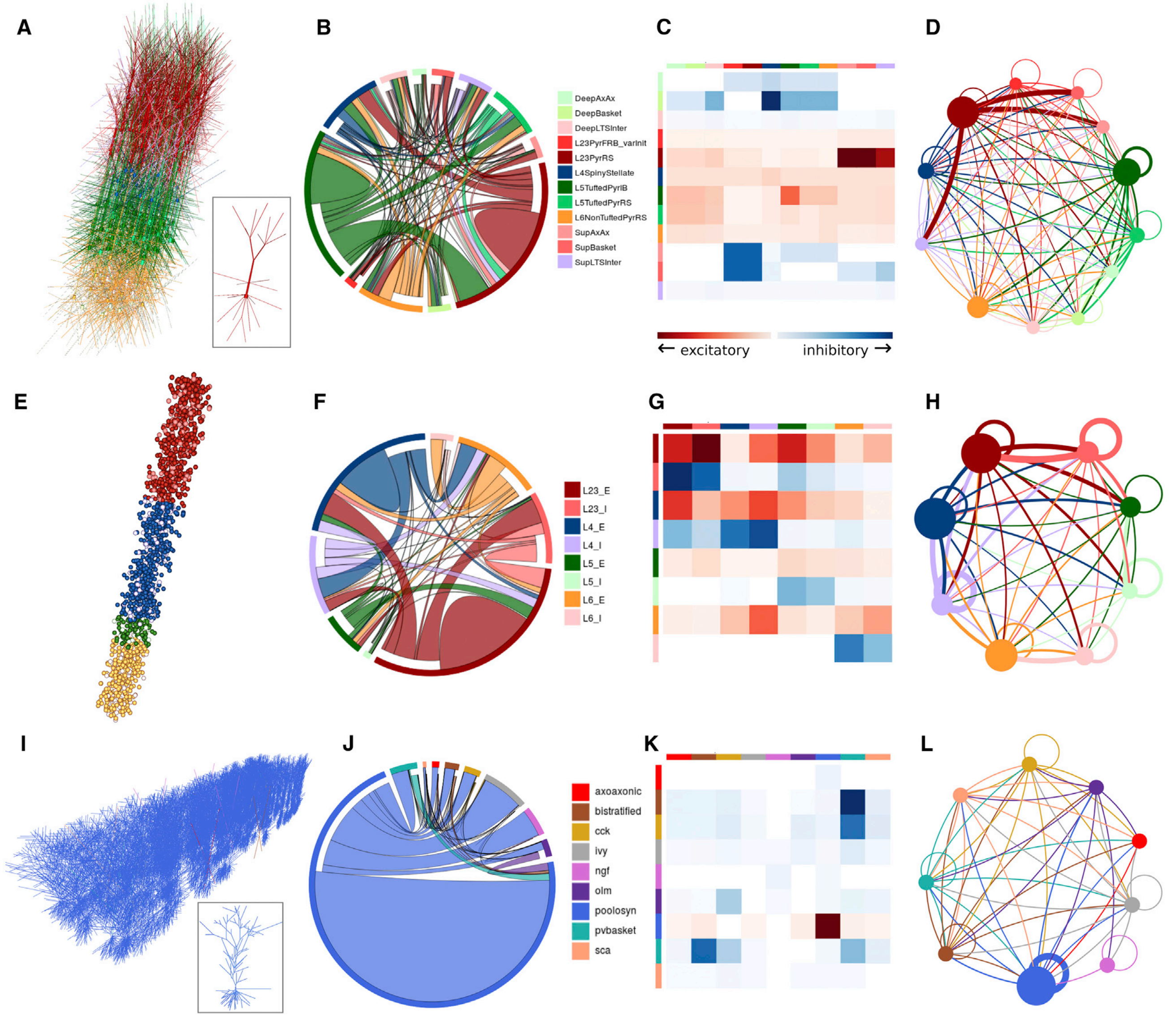

Figure 4. Analysis of Network Structure on OSB

(A) Single-column thalamocortical network model from Traub et al. (2005), containing 336 cells (between 100 and 148 compartments each; 10% of full network) in 12 populations. Inset shows example layer 2/3 pyramidal cell.

(B) Chord diagram showing projections between populations (1×10^5 individual connections of 193 types). Outer ring color indicates population; the chords are attached to the presynaptic population outer ring segment, separated from the postsynaptic population ring segment and colored to match the postsynaptic population. Colors for populations match 3D view in (A).

(C) Adjacency matrix; lines on left and top indicate pre- and postsynaptic population colors, respectively. Squares represent relative strengths of excitatory (red) or inhibitory (blue) inputs (average weighted conductance of synaptic input from the presynaptic population to each postsynaptic cell).

(D) Connectivity graph between populations; relative size of populations indicated by circle diameter; connection line widths scaled by weight. Both circle size and line weight have minimum values for visual clarity. Line colors match presynaptic population.

(E–H) 3D view of network (E), chord diagram (F), adjacency matrix (G), and connectivity graph (H) for point neuron spiking network model of Potjans and Diesmann (2014), with 1,539 cells in 8 populations (E [excitatory] and I [inhibitory] from layer 2/3, layer 4, layer 5, and layer 6; 5.96×10^4 connections; 2% of full-scale network).

(I–L) 3D view of network (I), chord diagram (J), adjacency matrix (K), and connectivity graph (L) for network model of hippocampal CA1 region from Bezaire et al. (2016) with 311 pyramidal cells (example cell in inset of I) and 24 interneurons of 8 different types. Network (0.1% of full scale) has over 1×10^6 connections, primarily between pyramidal cells. All images are screenshots from browser visualization.

See also Video S2.

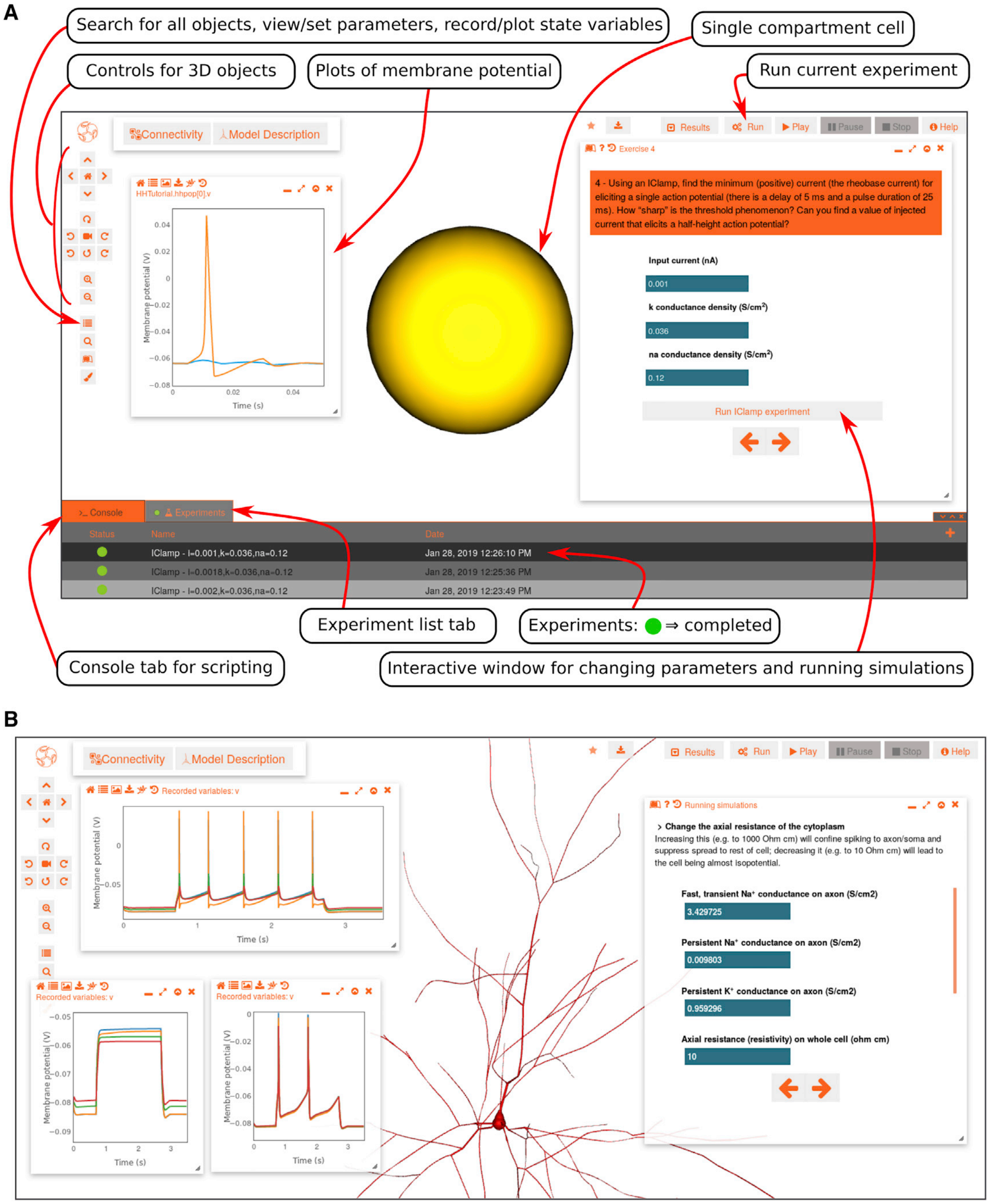

Figure 5. Interactive Online Tutorials Illustrating Control and Execution of Simulations

(A) Annotated screenshot of an OSB project for a single-compartment neuron with Hodgkin-Huxley type conductances (Hodgkin and Huxley, 1952). Single-compartment model cell (yellow sphere), tutorial control panel for altering current, channel densities, and running simulations (right), list of previously run experiments with changed parameters (bottom), tab for enabling the interactive command line console (bottom left), and membrane potential plot showing spiking (orange) and subthreshold (blue) recordings (left) are shown.

(B) Screenshot of interactive tutorial using a layer 2/3 pyramidal cell model (Markram et al., 2015) to illustrate how OSB represents biophysically detailed cells and how their functional properties can be explored. Right: interactive guide shows parameters that can be changed and suggestions for exploring behavior. Plots on left show membrane potential at multiple locations on cell (blue, soma; orange, end of axon; green and red, two dendritic locations) for 3 scenarios while 2-s current pulse is applied: original cell parameters (top); axonal sodium conductance removed (bottom left); and axial resistance reduced by factor of 10 (bottom right).

See also Video S3.

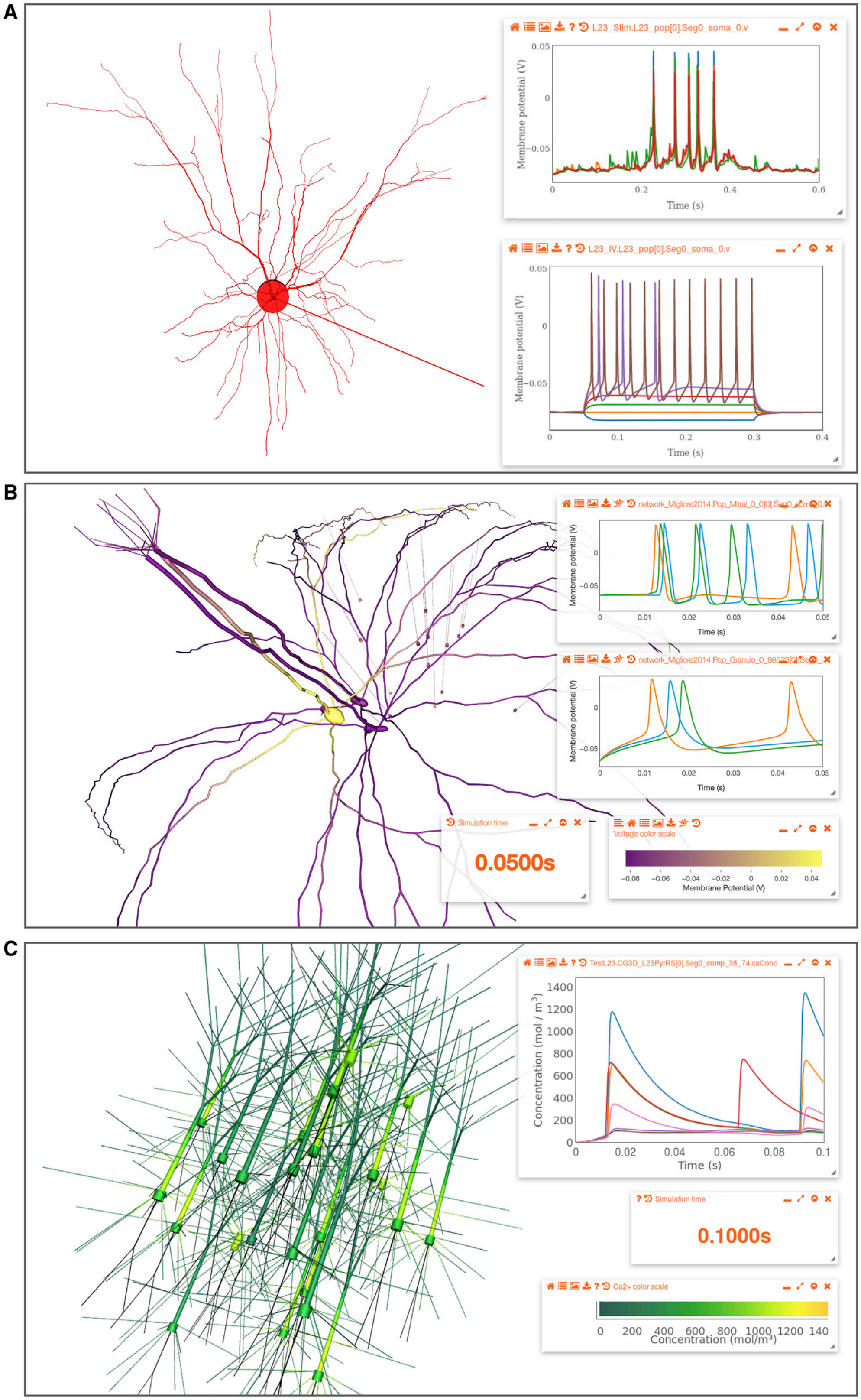

(legend on next page)

specific populations. The user also has control over the number of simulated variables recorded. For example, the membrane potential can be recorded from the soma or from all compartments in every cell. Recorded data can be replayed as variable-time plot or a pseudocolor representation can be used to indicate the voltage (Figure 6B) or calcium concentration (Figure 6C) across the morphology or across a population of neurons. The ability to analyze, visualize, and interact with models on OSB provides a unified online resource for accessing the structural and functional properties of complex models of brain function, thereby enabling greater scrutiny and insight into these powerful computational tools.

Simulation Management and Tutorials

Simulations of neurons and circuits generate a large amount of data. Moreover, to examine behavior under different conditions, models must be run many times. To deal with these requirements, we have built a system for managing and storing simulation experiments on OSB. This enables registered users to run multiple simulations and to interactively explore the results (Figure 5). The simulation results generated through OSB can also be downloaded in a zip file to the user's computer or automatically uploaded to Dropbox for more detailed offline analysis (STAR Methods). In addition, the layout of the visualization panels showing the 3D morphology of the model and associated analysis panels can be saved between sessions. All changes to the model and its graphical visualization are recorded as a series of text-based instructions, ensuring that the simulation, analysis, and presentation are fully documented (STAR Methods). These can be accessed through a popup console tab (Figure 5A, bottom left), which allows a series of instructions to be copied, pasted, and rerun, as well as direct control of OSB through scripting.

The web-based nature of OSB, together with its simulation and management features, make it well suited for demonstrating the principles of neurophysiology in an interactive and accessible format. To this end, we have built a framework for constructing online tutorials that can be used to explain concepts through presentation of figures and simulations. These features enable interactive tutorials and virtual experiments to be constructed that can be used to teach basic concepts in neurophysiology and computational neuroscience without the barrier of having to write code or install specialist simulators. To illustrate this functionality, we have extended a pre-existing tutorial on the Hodgkin Huxley model of the action potential for use on OSB (Figure 5A) and have created an interactive tutorial on modeling biophysically detailed, multicompartmental neurons using a layer 2/3 pyramidal cell from the Blue Brain Project (Markram et al., 2015; Figure 5B).

Adding Models to OSB

When a new project is added to OSB in a standardized format, the contributor immediately benefits from the automated visualization, analysis, and simulation to showcase their own model. Standardized formats also aid analysis and comparison of the properties and behavior of the cells from different sources (Figure S2). Although some models are originally developed in standardized formats (Cayco-Gajic et al., 2017), most existing models have been developed and defined in simulator-specific languages (McDougal et al., 2017) and therefore require conversion to NeuroML or PyNN. NeuroML is a widely used standardized model description language that is sufficiently flexible to define a wide range of models in neuroscience (Cannon et al., 2014; Gleeson et al., 2010). Models defined in NeuroML can be automatically "read" and visualized or transformed into the instructions required to run simulations (Table S2). PyNN is a Python-based language for describing models that is compatible with a range of simulators, including NEURON (Carnevale and Hines, 2006), NEST (Gewaltig and Diesmann, 2007), Brian (Goodman and Brette, 2008), and neuromorphic hardware (Schemmel et al., 2010). Although PyNN and NeuroML have different approaches to model specification, they are interoperable: networks can be created with PyNN scripts and the structure exported to NeuroML format (e.g., Figures 4E–4H) and specific cell models in NeuroML can be used in PyNN scripts and run on supported simulators (STAR Methods).

We have developed a range of documentation and tools to facilitate the conversion of models into NeuroML and PyNN (Figures 1A and 1B). Figure S1 provides an overview of how these tools can be used at each stage of conversion of an existing model for use on OSB. A key advantage of the modular structure of NeuroML and PyNN is that model components can be automatically tested across multiple simulators using the OSB Model Validation (OMV) framework (STAR Methods). This allows automated tests to be run to check the expected behavior of models every time there is a change to the code in the repository and helps ensure the quality of the model components. Table S2 shows the range of simulator-specific tests on the OSB models discussed in this paper. To facilitate local execution and testing of models, we have created a self-contained software environment (a Docker image; STAR Methods; Table S3) with all simulator tools preconfigured (Table S4), as well as verified, stable releases of all models presented here. Using this tool, 351 individual tests across 23 simulator configurations in 27 projects

Figure 6. Visualization of Simulations through the Browser

(A) Layer 2/3 pyramidal cell project from Smith et al. (2013). Left: 3D cell morphology is shown. Top right: membrane potential recorded at 5 locations from a simulation of this cell receiving background synaptic stimulation is shown. Bottom right: membrane potential at soma for increasing levels of current injected at the soma is shown.

(B) Small network with 3 mitral and 15 granule cells from Migliore et al. (2014). Plots on right show somatic membrane potentials for 3 mitral (top) and 3 granule (bottom) cells. Panels below show current simulation time and color scale for the recorded membrane potentials, as displayed on the cell morphologies during simulation replay.

(C) Left: small network of layer 2/3 pyramidal cells and interneurons from Traub et al. (2005). Right hand plot shows time course of somatic calcium concentration in 7 cells during the simulation. Right bottom, scale for recorded calcium concentration as overlaid on morphologies is shown.

See also Video S3.

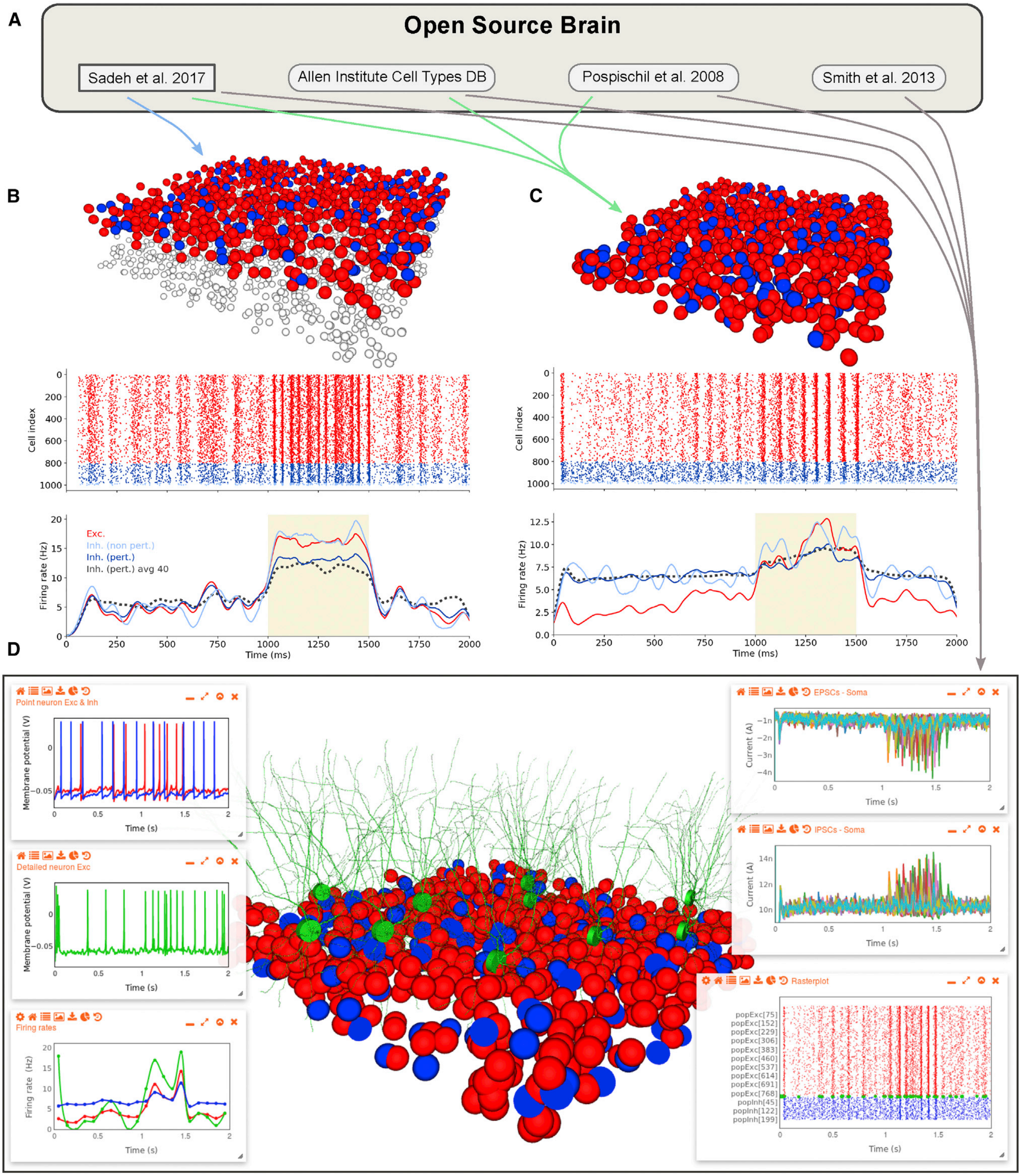

Figure 7. Creation of Biologically Detailed Models of Inhibition Stabilized Cortical Networks from Model Components on OSB

(A) OSB models that have been reused to build inhibition stabilized network (ISN) models with different levels of biophysical detail.

(B) Model of ISN created in PyNN, exported to NeuroML, and visualized on OSB (top; 800 excitatory [E; red], 200 inhibitory [I; blue] cells; external spiking inputs, modeled as explicit populations in PyNN, shown in white). Spiking behavior of cells (middle) and population rate plots (bottom) during reduced excitatory synaptic excitation to 90% of the I cells (during shaded period input rate was reduced from 9,600 Hz to 9,200 Hz in these cells) are shown. Population rate plots (bottom; average firing rate of subpopulations smoothened with Gaussian kernel of width 30 ms) reveal firing mean rate increases despite lower input to these cells (dark

(legend continued on next page)

can be run on any operating system supporting Docker (Table S3). This demonstrates the broad coverage of model types and simulators that can benefit from automated testing and will help ensure OSB models and components are reproducible.

Creating New Models from Existing Components on OSB

A key reason to make models and components available on OSB is that they can be reused and adapted to address new scientific questions. NeuroML software libraries (STAR Methods) can be used to create new models by reusing pre-existing components. To illustrate this, we built new cortical network models with differing biological detail by combining existing components using the tools we have developed for construction and optimization of NeuroML-based models (Figure S1). Linking the resulting model to OSB then enabled the visualization, management, and testing functionality to be used to adapt and refine the models.

Based on the connectivity and functional properties of the neocortex, it has been suggested that cortical networks operate in a regime with high excitatory gain, which renders the excitatory subnetwork unstable in the absence of strong feedback inhibition (Tsodyks et al., 1997). There is considerable interest in such inhibition-stabilized network (ISN) models (Garcia Del Molino et al., 2017; Joglekar et al., 2018; Ozeki et al., 2009; Rubin et al., 2015; Sadeh et al., 2017), as high-gain network regimes are thought to contribute to important functions, like signal amplification, noise tolerance, and pattern completion, and could underlie certain pathological states, such as epilepsy (Avoli et al., 1995; Mann et al., 2009). Moreover, recent experimental studies on the superficial layers of visual and auditory cortex support the idea that they operate as ISNs (Adesnik, 2017; Kato et al., 2017; Moore et al., 2018). Networks operating in ISN regimes can be identified through their characteristic "signature," which is a paradoxical inverse response of the inhibitory interneurons to alterations in excitatory drive (Tsodyks et al., 1997). This was predicted from highly simplified models where neuronal populations were modeled as single nodes and synaptic input was modeled as current. The simplicity of such models raises the question of whether more complex neuronal networks, composed of populations of excitatory and inhibitory neurons interconnected with more realistic recurrent synaptic connectivity and conductance-based signaling, behave in a similar manner (Sadeh et al., 2017). Moreover, real cortical neurons receive synaptic input onto extensive dendritic trees, which can exhibit nonlinear behavior (Stuart and Spruston, 2015). However, no previous model has explored whether ISN properties can be detected in neurons with realistic dendritic integration.

To test whether ISN signature behavior is to be expected in real cortical circuits, we built three network models of increasing biological detail using NeuroML components present on OSB and the associated tools for model construction (Figure 7A; STAR Methods). Reimplementation of the spiking network model from Sadeh et al. (2017) in PyNN, with adaptive exponential integrate-and-fire (I&F) cells, exhibited *increases* in the firing when the excitatory drive onto inhibitory cells was *decreased* (Figure 7B; cf. Figure 10B in Sadeh et al., 2017), hence confirming that the paradoxical signature of ISN could also be observed in these I&F networks. To investigate whether ISN responses occur with more realistic conductance-based spiking mechanisms, we reused the combination of membrane conductances from cortical cell models in Pospischil et al. (2008) and created single-compartment cell models that matched experimentally recorded behavior of layer 2/3 spiny (putative excitatory) and aspiny (putative inhibitory) cells from the Allen Cell Types Database (STAR Methods; Figures S3A–S3D). A network model constructed with these cells and with synaptic connectivity from Sadeh et al. (2017) also exhibited the paradoxical effect of ISNs (Figure 7C).

To examine whether dendritic integration affects the ability to detect ISNs, we built a hybrid network model where some of the single-compartment excitatory cells were substituted with morphologically detailed layer 2/3 pyramidal cell models (Smith et al., 2013; Figures S3E and S4A). Figure 7D shows a screenshot of OSB with a 3D view of the hybrid network and a number of visualization panels showing the network activity, including raster plots, firing rate traces, and membrane potential plots for individual cells. As for the networks of point neurons, this hybrid network exhibited an increase in firing rate upon reduced excitatory drive to inhibitory cells, with a particularly strong effect observed in the morphologically detailed neurons. Voltage-clamp recordings from 2 of the 10 morphologically complex cells revealed a burst of excitatory and inhibitory synaptic currents during the period of reduced excitatory drive to a subset of inhibitory cells (Figure 7D, top and middle panels on right). Similar responses were observed when a hyperpolarizing current was applied to interneurons to mimic optogenetic inactivation by halorhodopsin (Figures S4B–S4D), consistent with recent voltage-clamp recordings from layer 2/3 cortical pyramidal neurons (Kato et al., 2017).

These results show that ISN signature responses can occur in network models with large populations of excitatory and

blue; black dotted line is average of 40 simulations), as well as the rate of non-perturbed I (light blue) and E cells (red), confirming the presence of a signature ISN response under these conditions.

(C) Network model in NeuroML consisting of point neurons with voltage-gated membrane conductances from Pospischil et al. (2008) that were scaled to match the firing behavior of layer 2/3 spiny (E; red) and aspiny (I; blue) neurons from the Allen Cell Types Database. Network visualization, spike raster, and firing rate plots during a decreased excitatory drive to 90% of inhibitory cells as for (B) are shown. See also Figures S3A–S3D.

(D) Similar network to (C), with 10 of the E cells replaced by detailed layer 2/3 cell model from Smith et al. (2013; green cells in 3D view). A single screenshot of OSB showing the range of graphical elements that can be used to interactively investigate the behavior of the network. Top two plots on left show membrane potentials from a point E neuron (red) and an I cell (blue) as well as the detailed E cell (green). A spike raster plot (bottom right) and corresponding firing rate traces (bottom left; average rate in 100-ms window per population) are also shown (population colors follow 3D network). The top and middle right-hand plots show currents from 10 independent network simulations when the somata of two morphologically complex cells were voltage clamped at −80 mV and 0 mV, revealing the excitatory and inhibitory postsynaptic currents, respectively.

See also Figures S3B and S4.

inhibitory cells interconnected with the extensive recurrent synaptic connectivity and conductance-based signaling mechanisms as found in cortical networks. Moreover, they establish that the ISN signature responses can be detected from somatic voltage-clamp recordings in layer 2/3 pyramidal cells, even when the synaptic input is integrated across the dendritic arbor. These results demonstrate that components of NeuroML models on OSB can be reused with the new tools and infrastructure to build complex multiscale circuit models.

DISCUSSION

We have developed Open Source Brain, a web-based collaborative resource of standardized neuronal and circuit models together with tools and infrastructure for model development, testing, and reuse. The OSB platform enables web-browser-based visualization, analysis, and simulation of models without the need to install software or write code. This makes complex models accessible to the wider neuroscience community, enabling critical evaluation of model properties and behavior. The modular format used by OSB ensures that models and their components can more easily be reused for new scientific questions. By making neuronal and circuit models more accessible, transparent, and reliable, the OSB platform provides a powerful new resource for students, individual researchers, and collaborative research teams to learn about and investigate brain function in health and disease.

OSB's browser-based visualization of models of neurons and circuits and automated analysis of their structural and functional properties provides a wealth of information about model properties that was largely inaccessible to non-specialists. Moreover, by removing the technical barriers of having to write code to run and configure simulations on high-performance computing facilities, OSB's browser-controlled simulation functionality now makes it possible for anyone to explore the behavior of complex models. Advanced users have option of downloading any of the models and using the same standards-based toolchain on their own machines (STAR Methods). Academics can also use the OSB online tutorial building functionality to build interactive teaching resources that combine text and simulations to illustrate diverse neurophysiological phenomena. Allowing a wider range of users to access such detailed models will facilitate critical evaluation from the wider neuroscience community.

OSB is designed so that, when a new project is added by a contributor, they immediately benefit from functionality to showcase their own model (Figure S1). An important aspect of OSB is the provision of infrastructure to facilitate the continuous open source development, refinement, and testing of models. This is enabled through standardized model descriptions, new tools for automatically testing code, and deep integration with the code development platform GitHub, which enables collaborative software development. The OSB Model Validation framework, which uses code-testing methodologies from software engineering, helps ensure model behavior does not change when converted to standardized formats and when updates are made to the code. This can maintain code quality and consistency, enabling complex models to be kept up to date with new experimental results, without introducing errors. The large battery of tests that have already been applied across models and components by this framework (Table S2) enables an unprecedented level of reproducibility to be obtained on OSB.

The fact that model development and testing can be carried out without the need for OSB administrators to get involved will enable the resource to expand in a way that is determined by the user's interests and research goals. Models contributed by individual researchers and labs are complemented with community-developed models, including the Human Brain Project (HBP) HippoCamp initiative, for which our CA1 network conversion (Figures 4I–4L) is a first major contribution, and the OpenWorm project (Sarma et al., 2018; Szigeti et al., 2014), which aims to create a detailed computational model of the nematode *C. elegans*.

The distinct functionality of OSB extends and complements that of ModelDB (McDougal et al., 2017), a well-established repository of models in computational neuroscience. ModelDB hosts model code in the original language in which it was developed and facilitates the reproduction of the results from their originating publications. OSB builds on this functionality by focusing on hosting standardized models that are independent of the simulator used, which enable users to interact with and analyze models in greater detail. Moreover, OSB is designed to reveal circuit-level properties, including connectivity and network dynamics (Figures 4 and 7). More fundamentally, models hosted on OSB are not static as they are in ModelDB and can instead be developed, refined, and improved using the infrastructure for open source software development, automated validation, and testing of models. Deep links between these resources allow users to find the same model on either platform. Indeed, OSB actively encourages researchers to first submit their model to ModelDB following publication (Figure S1). The step of moving a model onto OSB is an indication that one or more parties (who may not be the original developers) wish to standardize and further develop the model, making it more accessible to the wider neuroscience community and extending it for use beyond the original publication. OSB also supports sharing of model code prior to publication, e.g., the ongoing development of a large-scale network model of primary motor cortex (Dura-Bernal et al., 2017).

OSB interacts with other online resources that provide structured, annotated data that are invaluable for creating and validating neuronal models. For example, reconstructed neuronal morphologies from NeuroMorpho.Org (Ascoli et al., 2007) and the Janelia MouseLight project (Economo et al., 2016) can be automatically converted to NeuroML and visualized through OSB, and these morphologies can become the basis for new models when combined with cell-specific membrane conductances already expressed in NeuroML. The Allen Institute Cell Types Database (Hawrylycz et al., 2016) provides electrophysiological recordings and morphological reconstructions from cells in mouse visual cortex. Biophysically detailed cell models and point neuron models based on these data are present on OSB, and new NeuroML-based models can be generated from the source data (Figures S3A–S3D). Models currently being used in the HBP (Amunts et al., 2016) have also been converted, including cell models from the Blue Brain Project's reconstruction of the microcircuitry of rat somatosensory cortex

(Markram et al., 2015), as well as a reduced version of a cortical column (Potjans and Diesmann, 2014). Converting the neuronal models present in these networks to standardized formats provides a valuable resource for developing new models of cortical circuits from modular, well-tested building blocks.

As our development of biologically detailed cortical network models illustrates, new models can be built from the existing standardized components present on OSB by optimizing them against available data (Figure 7). By using model elements from different projects to build ISN network models with different degrees of biological detail, we show that the ISN signature responses that were predicted from highly simplified models (Sadeh et al., 2017; Tsodyks et al., 1997) are also expected in biologically detailed models that include conductance-based spiking mechanisms and can be detected in cells with complex dendritic morphologies. Predictions from these models can be used to refine experimental approaches for detecting ISNs (Adesnik, 2017; Kato et al., 2017; Moore et al., 2018), to explain why they might not be detectable under some experimental conditions, and to investigate how dendritic properties interact with the nonlinear dynamics of ISNs at the network level.

The models of neurons and circuits on the OSB platform complement standardization and online simulation in other areas of biology. In systems biology, biochemical signaling pathways, often expressed in standardized systems biology markup language (SBML) format (Hucka et al., 2003), can be analyzed and executed online by using, for example, VCell (Loew and Schaff, 2001) or on JWS (Olivier and Snoep, 2004). Expanding NeuroML's existing functionality for interacting with SBML (Cannon et al., 2014) will allow easier integration of complex biochemical reactions into NeuroML-based models of neuronal and circuit models. It will also enable greater interoperability between OSB and databases such as BioModels (Le Novère et al., 2006) and the Physiome Model Repository (Yu et al., 2011). In addition to these subcellular model specifications, NeuroML is currently being extended to support more high-level, population-based models (e.g., the Wilson and Cowan model; Wilson and Cowan, 1972) which will potentially enable the analysis and simulation on OSB of brain-scale networks, as supported by a number of simulators, such as The Virtual Brain (Sanz Leon et al., 2013).

Building models from new and existing components currently involves configuring and optimizing the model offline and then loading the code to a repository on GitHub, where OSB can validate and test it prior to visualization, analysis, and simulation. In order to make this process easier, we plan to combine elements of the user interface of NetPyNE (a Python-based platform for model creation built on top of the NEURON simulator; Dura-Bernal et al., 2019) with OSB to provide an online model construction, optimization, and testing interface. This will be facilitated by the fact that the online visualization interfaces of both OSB and NetPyNE are built using the Geppetto platform (Cantarelli et al., 2018; STAR Methods). To further lower the barriers to model creation on OSB, we are also expanding the functionality of the platform to include tighter integration with the experimental data used to build models and test their performance. Standardized formats for experimental data, such as Neurodata without Borders (https://nwb.org), will be crucial for this. Hosting standardized models and the data from which they are built will provide all the necessary information for model optimization, thereby providing the functionality to refine and adapt models as new experimental results become available. Direct comparison of experimental data with model properties will provide a new level of transparency and scrutiny for data-driven models. These developments, together with the current functionality of OSB that facilitates accessibility and the construction of models from modular reusable components, will accelerate the pace of model building and reuse by the wider neuroscience community.

STAR★METHODS

Detailed methods are provided in the online version of this paper and include the following:

- KEY RESOURCES TABLE
- CONTACT FOR REAGENT AND RESOURCE SHARING
- METHOD DETAILS
  - OSB frontend: project and user management
  - OSB frontend: visualizing & simulating models
  - NeuroML 2 & LEMS libraries
  - PyNN models
  - Executing simulations on OSB
  - Interactions with neuroinformatics resources
  - Testing and model validation
  - Inhibition stabilized network models
- DATA AND SOFTWARE AVAILABILITY

SUPPLEMENTAL INFORMATION

Supplemental Information can be found online at https://doi.org/10.1016/j.neuron.2019.05.019.

ACKNOWLEDGMENTS

This work was funded by the Wellcome Trust (086699, 101445, 212941, and 095667) and an ERC advanced grant (294667) to R.A.S., who is in receipt of a Wellcome Trust Principal Research Fellowship (203048). OSB and NSG integration was funded by the BBSRC-NSF/BIO program (BB/N005236/1 and NSF no. 1458840) and NSF no. 1458495. E.P. was supported on the EU Marie Curie Initial Training Network CEREBNET (FP7-ITN-PEOPLE-2008; 238686). S.D.-B. and W.W.L. were funded by NIBIB U01EB017695, DOH01-C32250GG-3450000, and NIH R01EB022903. M.L.H. and R.A.M. were funded by NIH grant DC009977 and M.L.H. by NIH NS11613. B.M. was funded by FAPESP, proc. 2017/04748-0. S.C. and J.B. were partially funded by NIH R01MH106674 and NIH R01EB021711. S.J.v.A. and A.P.D. were funded by EU 7th Framework Program (FP7/2007-2013) under grant agreement no. 604102 (Human Brain Project, ramp up phase) and EU Horizon 2020 grant no. 720270 (HBP, SGA1). A.E., J.B., and R.S. were partially funded by the Google Summer of Code program, coordinated through the INCF. We thank Marianne Bezaire for assistance in converting the CA1 network model and Eilif Muller for help converting the Blue Brain Project cells. We would like to thank the other developers who have made contributions to OSB models and supporting applications. Specific contributions can be found on individual GitHub repositories. We thank Andreas Schaefer, Tommy Younts, and Diccon Coyle for feedback on the manuscript.

AUTHOR CONTRIBUTIONS

P.G. and R.A.S. conceived the Open Source Brain project. P.G., R.A.S., S.C., R.C.C., M.L.H., B.M., J.B., and E.P. contributed to the NeuroML language and tools. P.G. and A.P.D. developed the PyNN/NeuroML toolchain. M.C. oversaw

development of Geppetto functionality, and M.C., G.I., A.Q., B.M., M.E., P.G., and R.A.S. contributed to design and development of Geppetto features. M.C., A.Q., B.M., M.E., E.P., P.G., G.I., and R.A.S. contributed to design and development of the OSB platform, including Redmine-based frontend. P.G., B.M., E.P., S. Sadeh, N.A.C.-G., R.S., A.E., A.P.D., J.B., S. Solinas, S.D.-B., S.J.v.A., W.v.G., S.D.L., and F.L. converted models to standardized formats. S. Sadeh, P.G., and R.A.S. developed the ISN network models. B.M. and P.G. developed OMV testing framework. P.G. and R.A.M. developed links between OSB and ModelDB. P.G., A.Q., A.M., and S. Sivagnanam developed interface with NSG. S.D.-B. and W.W.L. contributed to the NetPyNE mapping for models. P.G. and R.A.S. wrote the manuscript with input from all authors.

DECLARATION OF INTERESTS

The work carried out for this publication by R.C.C. was conducted under contract from UCL to his employer, Annotate Software Limited, in which he is also a shareholder. MetaCell Ltd. was also contracted by UCL to develop some of the features of the Open Source Brain software. M.C., G.I., and S.D.L. declare financial interest in MetaCell Ltd.

Received: July 5, 2018
Revised: March 4, 2019
Accepted: May 9, 2019
Published: June 11, 2019

REFERENCES

Adesnik, H. (2017). Synaptic mechanisms of feature coding in the visual cortex of awake mice. Neuron *95*, 1147–1159.e4.

Ahrens, M.B., Orger, M.B., Robson, D.N., Li, J.M., and Keller, P.J. (2013). Whole-brain functional imaging at cellular resolution using light-sheet microscopy. Nat. Methods *10*, 413–420.

Amunts, K., Ebell, C., Muller, J., Telefont, M., Knoll, A., and Lippert, T. (2016). The Human Brain Project: creating a European research infrastructure to decode the human brain. Neuron *92*, 574–581.

Annecchino, L.A., Morris, A.R., Copeland, C.S., Agabi, O.E., Chadderton, P., and Schultz, S.R. (2017). Robotic automation of in vivo two-photon targeted whole-cell patch-clamp electrophysiology. Neuron *95*, 1048–1055.e3.

Ascoli, G.A., Donohue, D.E., and Halavi, M. (2007). NeuroMorpho.Org: a central resource for neuronal morphologies. J. Neurosci. *27*, 9247–9251.

Avoli, M., Louvel, J., Drapeau, C., Pumain, R., and Kurcewicz, I. (1995). GABAA-mediated inhibition and in vitro epileptogenesis in the human neocortex. J. Neurophysiol. *73*, 468–484.

Bezaire, M.J., Raikov, I., Burk, K., Vyas, D., and Soltesz, I. (2016). Interneuronal mechanisms of hippocampal theta oscillations in a full-scale model of the rodent CA1 circuit. eLife *5*, e18566.

Billings, G., Piasini, E., Lőrincz, A., Nusser, Z., and Silver, R.A. (2014). Network structure within the cerebellar input layer enables lossless sparse encoding. Neuron *83*, 960–974.

Boyle, J.H., and Cohen, N. (2008). Caenorhabditis elegans body wall muscles are simple actuators. Biosystems *94*, 170–181.

Brette, R., and Gerstner, W. (2005). Adaptive exponential integrate-and-fire model as an effective description of neuronal activity. J. Neurophysiol. *94*, 3637–3642.

Brunel, N. (2000). Dynamics of sparsely connected networks of excitatory and inhibitory spiking neurons. J. Comput. Neurosci. *8*, 183–208.

Cain, N., Iyer, R., Koch, C., and Mihalas, S. (2016). The computational properties of a simplified cortical column model. PLoS Comput. Biol. *12*, e1005045.

Cannon, R.C., Turner, D.A., Pyapali, G.K., and Wheal, H.V. (1998). An on-line archive of reconstructed hippocampal neurons. J. Neurosci. Methods *84*, 49–54.

Cannon, R.C., Gleeson, P., Crook, S., Ganapathy, G., Marin, B., Piasini, E., and Silver, R.A. (2014). LEMS: a language for expressing complex biological models in concise and hierarchical form and its use in underpinning NeuroML 2. Front. Neuroinform. *8*, 79.

Cantarelli, M., Marin, B., Quintana, A., Earnshaw, M., Court, R., Gleeson, P., Dura-Bernal, S., Silver, R.A., and Idili, G. (2018). Geppetto: a reusable modular open platform for exploring neuroscience data and models. Philos. Trans. R. Soc. Lond. B Biol. Sci. *373*, 20170380.

Carnevale, N.T., and Hines, M.L. (2006). The NEURON Book (Cambridge University Press).

Cayco-Gajic, N.A., Clopath, C., and Silver, R.A. (2017). Sparse synaptic connectivity is required for decorrelation and pattern separation in feedforward networks. Nat. Commun. *8*, 1116.

Davison, A.P., Brüderle, D., Eppler, J., Kremkow, J., Muller, E., Pecevski, D., Perrinet, L., and Yger, P. (2009). PyNN: a common interface for neuronal network simulators. Front. Neuroinform. *2*, 11.

Dayan, P., and Abbott, L.F. (2001). Theoretical Neuroscience (MIT Press).

Del Castillo, J., and Katz, B. (1954). Quantal components of the end-plate potential. J. Physiol. *124*, 560–573.

Diesmann, M., Gewaltig, M.O., and Aertsen, A. (1999). Stable propagation of synchronous spiking in cortical neural networks. Nature *402*, 529–533.

Dura-Bernal, S., Neymotin, S.A., Kerr, C.C., Sivagnanam, S., Majumdar, A., Francis, J.T., and Lytton, W.W. (2017). Evolutionary algorithm optimization of biological learning parameters in a biomimetic neuroprosthesis. IBM J. Res. Dev. *61*, 6.1–6.14.

Dura-Bernal, S., Suter, B.A., Gleeson, P., Cantarelli, M., Quintana, A., Rodriguez, F., Kedziora, D.J., Chadderdon, G.L., Kerr, C.C., Neymotin, S.A., et al. (2019). NetPyNE, a tool for data-driven multiscale modeling of brain circuits. eLife *8*, e44494.

Duvall, P.M., Matyas, S., and Glover, A. (2007). Continuous Integration: Improving Software Quality and Reducing Risk (Pearson Education).

Economo, M.N., Clack, N.G., Lavis, L.D., Gerfen, C.R., Svoboda, K., Myers, E.W., and Chandrashekar, J. (2016). A platform for brain-wide imaging and reconstruction of individual neurons. eLife *5*, e10566.

Eglen, S.J., Marwick, B., Halchenko, Y.O., Hanke, M., Sufi, S., Gleeson, P., Silver, R.A., Davison, A.P., Lanyon, L., Abrams, M., et al. (2017). Toward standard practices for sharing computer code and programs in neuroscience. Nat. Neurosci. *20*, 770–773.

Ferguson, K.A., Huh, C.Y.L., Amilhon, B., Williams, S., and Skinner, F.K. (2013). Experimentally constrained CA1 fast-firing parvalbumin-positive interneuron network models exhibit sharp transitions into coherent high frequency rhythms. Front. Comput. Neurosci. *7*, 144.

Fitzhugh, R. (1961). Impulses and physiological states in theoretical models of nerve membrane. Biophys. J. *1*, 445–466.

Gal, E., London, M., Globerson, A., Ramaswamy, S., Reimann, M.W., Muller, E., Markram, H., and Segev, I. (2017). Rich cell-type-specific network topology in neocortical microcircuitry. Nat. Neurosci. *20*, 1004–1013.

Garcia Del Molino, L.C., Yang, G.R., Mejias, J.F., and Wang, X.-J. (2017). Paradoxical response reversal of top-down modulation in cortical circuits with three interneuron types. eLife *6*, e29742.

Gewaltig, M.-O., and Diesmann, M. (2007). NEST (NEural Simulation Tool). Scholarpedia *2*, 1430.

Gleeson, P., Steuber, V., and Silver, R.A. (2007). neuroConstruct: a tool for modeling networks of neurons in 3D space. Neuron *54*, 219–235.

Gleeson, P., Crook, S., Cannon, R.C., Hines, M.L., Billings, G.O., Farinella, M., Morse, T.M., Davison, A.P., Ray, S., Bhalla, U.S., et al. (2010). NeuroML: a language for describing data driven models of neurons and networks with a high degree of biological detail. PLoS Comput. Biol. *6*, e1000815.

Goodman, D., and Brette, R. (2008). Brian: a simulator for spiking neural networks in python. Front. Neuroinform. *2*, 5.

Guzman, S.J., Schlögl, A., Frotscher, M., and Jonas, P. (2016). Synaptic mechanisms of pattern completion in the hippocampal CA3 network. Science *353*, 1117–1123.

Hawrylycz, M., Anastassiou, C., Arkhipov, A., Berg, J., Buice, M., Cain, N., Gouwens, N.W., Gratiy, S., Iyer, R., Lee, J.H., et al.; MindScope (2016). Inferring cortical function in the mouse visual system through large-scale systems neuroscience. Proc. Natl. Acad. Sci. USA *113*, 7337–7344.

Hay, E., Hill, S., Schürmann, F., Markram, H., and Segev, I. (2011). Models of neocortical layer 5b pyramidal cells capturing a wide range of dendritic and perisomatic active properties. PLoS Comput. Biol. *7*, e1002107.

Helmstaedter, M., Briggman, K.L., Turaga, S.C., Jain, V., Seung, H.S., and Denk, W. (2013). Connectomic reconstruction of the inner plexiform layer in the mouse retina. Nature *500*, 168–174.

Herz, A.V.M., Gollisch, T., Machens, C.K., and Jaeger, D. (2006). Modeling single-neuron dynamics and computations: a balance of detail and abstraction. Science *314*, 80–85.

Hodgkin, A.L., and Huxley, A.F. (1952). A quantitative description of membrane current and its application to conduction and excitation in nerve. J. Physiol. *117*, 500–544.

Hofer, S.B., Ko, H., Pichler, B., Vogelstein, J., Ros, H., Zeng, H., Lein, E., Lesica, N.A., and Mrsic-Flogel, T.D. (2011). Differential connectivity and response dynamics of excitatory and inhibitory neurons in visual cortex. Nat. Neurosci. *14*, 1045–1052.

Hucka, M., Finney, A., Sauro, H.M., Bolouri, H., Doyle, J.C., Kitano, H., Arkin, A.P., Bornstein, B.J., Bray, D., Cornish-Bowden, A., et al.; SBML Forum (2003). The systems biology markup language (SBML): a medium for representation and exchange of biochemical network models. Bioinformatics *19*, 524–531.

Insel, T.R., Landis, S.C., and Collins, F.S.; The NIH BRAIN Initiative (2013). Research priorities. Science *340*, 687–688.

Izhikevich, E.M. (2003). Simple model of spiking neurons. IEEE Trans. Neural Netw. *14*, 1569–1572.

Joglekar, M.R., Mejias, J.F., Yang, G.R., and Wang, X.-J. (2018). Inter-areal balanced amplification enhances signal propagation in a large-scale circuit model of the primate cortex. Neuron *98*, 222–234.e8.

Kandel, E.R., Markram, H., Matthews, P.M., Yuste, R., and Koch, C. (2013). Neuroscience thinks big (and collaboratively). Nat. Rev. Neurosci. *14*, 659–664.

Kasthuri, N., Hayworth, K.J., Berger, D.R., Schalek, R.L., Conchello, J.A., Knowles-Barley, S., Lee, D., Vázquez-Reina, A., Kaynig, V., Jones, T.R., et al. (2015). Saturated reconstruction of a volume of neocortex. Cell *162*, 648–661.

Kato, H.K., Asinof, S.K., and Isaacson, J.S. (2017). Network-level control of frequency tuning in auditory cortex. Neuron *95*, 412–423.e4.

Le Novère, N., Bornstein, B., Broicher, A., Courtot, M., Donizelli, M., Dharuri, H., Li, L., Sauro, H., Schilstra, M., Shapiro, B., et al. (2006). BioModels Database: a free, centralized database of curated, published, quantitative kinetic models of biochemical and cellular systems. Nucleic Acids Res. *34*, D689–D691.

Lee, J.H., Koch, C., and Mihalas, S. (2017). A computational analysis of the function of three inhibitory cell types in contextual visual processing. Front. Comput. Neurosci. *11*, 28.

Loew, L.M., and Schaff, J.C. (2001). The Virtual Cell: a software environment for computational cell biology. Trends Biotechnol. *19*, 401–406.

Maex, R., and De Schutter, E. (1998). Synchronization of golgi and granule cell firing in a detailed network model of the cerebellar granule cell layer. J. Neurophysiol. *80*, 2521–2537.

Mann, E.O., Kohl, M.M., and Paulsen, O. (2009). Distinct roles of GABA(A) and GABA(B) receptors in balancing and terminating persistent cortical activity. J. Neurosci. *29*, 7513–7518.

Markram, H., Muller, E., Ramaswamy, S., Reimann, M.W., Abdellah, M., Sanchez, C.A., Ailamaki, A., Alonso-Nanclares, L., Antille, N., Arsever, S., et al. (2015). Reconstruction and simulation of neocortical microcircuitry. Cell *163*, 456–492.

McDougal, R.A., Morse, T.M., Carnevale, T., Marenco, L., Wang, R., Migliore, M., Miller, P.L., Shepherd, G.M., and Hines, M.L. (2017). Twenty years of ModelDB and beyond: building essential modeling tools for the future of neuroscience. J. Comput. Neurosci. *42*, 1–10.

Migliore, M., Ferrante, M., and Ascoli, G.A. (2005). Signal propagation in oblique dendrites of CA1 pyramidal cells. J. Neurophysiol. *94*, 4145–4155.

Migliore, M., Cavarretta, F., Hines, M.L., and Shepherd, G.M. (2014). Distributed organization of a brain microcircuit analyzed by three-dimensional modeling: the olfactory bulb. Front. Comput. Neurosci. *8*, 50.

Moore, A.K., Weible, A.P., Balmer, T.S., Trussell, L.O., and Wehr, M. (2018). Rapid rebalancing of excitation and inhibition by cortical circuitry. Neuron *97*, 1341–1355.e6.

Olivier, B.G., and Snoep, J.L. (2004). Web-based kinetic modelling using JWS Online. Bioinformatics *20*, 2143–2144.

Ozeki, H., Finn, I.M., Schaffer, E.S., Miller, K.D., and Ferster, D. (2009). Inhibitory stabilization of the cortical network underlies visual surround suppression. Neuron *62*, 578–592.

Perkel, J. (2016). Democratic databases: science on GitHub. Nature *538*, 127–128.

Pinsky, P.F., and Rinzel, J. (1994). Intrinsic and network rhythmogenesis in a reduced Traub model for CA3 neurons. J. Comput. Neurosci. *1*, 39–60.

Pospischil, M., Toledo-Rodriguez, M., Monier, C., Piwkowska, Z., Bal, T., Frégnac, Y., Markram, H., and Destexhe, A. (2008). Minimal Hodgkin-Huxley type models for different classes of cortical and thalamic neurons. Biol. Cybern. *99*, 427–441.

Potjans, T.C., and Diesmann, M. (2014). The cell-type specific cortical microcircuit: relating structure and activity in a full-scale spiking network model. Cereb. Cortex *24*, 785–806.

Prinz, A.A., Bucher, D., and Marder, E. (2004). Similar network activity from disparate circuit parameters. Nat. Neurosci. *7*, 1345–1352.

Rall, W. (1962). Electrophysiology of a dendritic neuron model. Biophys. J. *2*, 145–167.

Ramaswamy, S., Courcol, J.-D., Abdellah, M., Adaszewski, S.R., Antille, N., Arsever, S., Atenekeng, G., Bilgili, A., Brukau, Y., Chalimourda, A., et al. (2015). The neocortical microcircuit collaboration portal: a resource for rat somatosensory cortex. Front. Neural Circuits *9*, 44.

Ranjan, R., Khazen, G., Gambazzi, L., Ramaswamy, S., Hill, S.L., Schürmann, F., and Markram, H. (2011). Channelpedia: an integrative and interactive database for ion channels. Front. Neuroinform. *5*, 36.

Ray, S., and Bhalla, U.S. (2008). PyMOOSE: interoperable scripting in Python for MOOSE. Front. Neuroinform. *2*, 6.

Reimann, M.W., Horlemann, A.-L., Ramaswamy, S., Muller, E.B., and Markram, H. (2017). Morphological diversity strongly constrains synaptic connectivity and plasticity. Cereb. Cortex *27*, 4570–4585.

Rubin, D.B., Van Hooser, S.D., and Miller, K.D. (2015). The stabilized supralinear network: a unifying circuit motif underlying multi-input integration in sensory cortex. Neuron *85*, 402–417.

Sadeh, S., Silver, R.A., Mrsic-Flogel, T.D., and Muir, D.R. (2017). Assessing the role of inhibition in stabilizing neocortical networks requires large-scale perturbation of the inhibitory population. J. Neurosci. *37*, 12050–12067.

Sanz Leon, P., Knock, S.A., Woodman, M.M., Domide, L., Mersmann, J., McIntosh, A.R., and Jirsa, V. (2013). The Virtual Brain: a simulator of primate brain network dynamics. Front. Neuroinform. *7*, 10.

Sarma, G.P., Lee, C.W., Portegys, T., Ghayoomie, V., Jacobs, T., Alicea, B., Cantarelli, M., Currie, M., Gerkin, R.C., Gingell, S., et al. (2018). OpenWorm: overview and recent advances in integrative biological simulation of *Caenorhabditis elegans*. Philos. Trans. R. Soc. Lond. B Biol. Sci. *373*, 20170382.

Schemmel, J., Briiderle, D., Griibl, A., Hock, M., Meier, K., and Millner, S. (2010). A wafer-scale neuromorphic hardware system for large-scale neural modeling. In Proceedings of 2010 IEEE International Symposium on Circuits and Systems (IEEE), pp. 1947–1950.

Schmidt, M., Bakker, R., Hilgetag, C.C., Diesmann, M., and van Albada, S.J. (2018a). Multi-scale account of the network structure of macaque visual cortex. Brain Struct. Funct. *223*, 1409–1435.

Schmidt, M., Bakker, R., Shen, K., Bezgin, G., Diesmann, M., and van Albada, S.J. (2018b). A multi-scale layer-resolved spiking network model of resting-state dynamics in macaque visual cortical areas. PLoS Comput. Biol. *14*, e1006359.

Schwalger, T., Deger, M., and Gerstner, W. (2017). Towards a theory of cortical columns: From spiking neurons to interacting neural populations of finite size. PLoS Comput. Biol. *13*, e1005507.

Sejnowski, T.J., Koch, C., and Churchland, P.S. (1988). Computational neuroscience. Science *241*, 1299–1306.

Sivagnanam, S., Majumdar, A., Yoshimoto, K., Astakhov, V., Bandrowski, A., Martone, M.E., and Carnevale, N.T. (2013). Introducing the neuroscience gateway. In CEUR Workshop Proceedings (IWSG).

Smith, S.L., Smith, I.T., Branco, T., and Häusser, M. (2013). Dendritic spikes enhance stimulus selectivity in cortical neurons in vivo. Nature *503*, 115–120.

Solinas, S., Forti, L., Cesana, E., Mapelli, J., De Schutter, E., and D'Angelo, E. (2007). Computational reconstruction of pacemaking and intrinsic electroresponsiveness in cerebellar Golgi cells. Front. Cell. Neurosci. *1*, 2.

Stuart, G.J., and Spruston, N. (2015). Dendritic integration: 60 years of progress. Nat. Neurosci. *18*, 1713–1721.

Szigeti, B., Gleeson, P., Vella, M., Khayrulin, S., Palyanov, A., Hokanson, J., Currie, M., Cantarelli, M., Idili, G., and Larson, S. (2014). OpenWorm: an open-science approach to modeling Caenorhabditis elegans. Front. Comput. Neurosci. *8*, 137.

Traub, R.D., Contreras, D., Cunningham, M.O., Murray, H., LeBeau, F.E., Roopun, A., Bibbig, A., Wilent, W.B., Higley, M.J., and Whittington, M.A. (2005). Single-column thalamocortical network model exhibiting gamma oscillations, sleep spindles, and epileptogenic bursts. J. Neurophysiol. *93*, 2194–2232.

Tsodyks, M.V., Skaggs, W.E., Sejnowski, T.J., and McNaughton, B.L. (1997). Paradoxical effects of external modulation of inhibitory interneurons. J. Neurosci. *17*, 4382–4388.

Vella, M., Cannon, R.C., Crook, S., Davison, A.P., Ganapathy, G., Robinson, H.P.C., Silver, R.A., and Gleeson, P. (2014). libNeuroML and PyLEMS: using Python to combine procedural and declarative modeling approaches in computational neuroscience. Front. Neuroinform. *8*, 38.

Vervaeke, K., Lőrincz, A., Gleeson, P., Farinella, M., Nusser, Z., and Silver, R.A. (2010). Rapid desynchronization of an electrically coupled interneuron network with sparse excitatory synaptic input. Neuron *67*, 435–451.

Wang, X.-J., and Buzsáki, G. (1996). Gamma oscillation by synaptic inhibition in a hippocampal interneuronal network model. J. Neurosci. *16*, 6402–6413.

Wilson, H.R., and Cowan, J.D. (1972). Excitatory and inhibitory interactions in localized populations of model neurons. Biophys. J. *12*, 1–24.

Yu, T., Lloyd, C.M., Nickerson, D.P., Cooling, M.T., Miller, A.K., Garny, A., Terkildsen, J.R., Lawson, J., Britten, R.D., Hunter, P.J., and Nielsen, P.M. (2011). The Physiome Model Repository 2. Bioinformatics *27*, 743–744.

STAR★METHODS

KEY RESOURCES TABLE

REAGENT or RESOURCE	SOURCE	IDENTIFIER
Software and Algorithms		
OSB user management web interface	This paper	https://github.com/OpenSourceBrain/redmine
OSB visualization/simulation frontend	This paper	https://github.com/openworm/org.geppetto
NeuroML	Cannon et al., 2014	https://www.neuroml.org; RRID: SCR_003083
PyNN	Davison et al., 2009	http://neuralensemble.org/PyNN; RRID: SCR_002715
NEURON	Carnevale and Hines, 2006	https://www.neuron.yale.edu; RRID: SCR_005393
NetPyNE	Dura-Bernal et al., 2019	http://netpyne.org, RRID: SCR_014758
NEST	Gewaltig and Diesmann, 2007	https://www.nest-simulator.org; RRID: SCR_002963
Brian	Goodman and Brette, 2008	http://briansimulator.org; RRID: SCR_002998
MOOSE	Ray and Bhalla, 2008	https://moose.ncbs.res.in; RRID: SCR_008031
Other		
Allen Cell Types Database	Hawrylycz et al., 2016	http://celltypes.brain-map.org; RRID: SCR_015719
Blue Brain Project's Neocortical Microcircuit Collaboration Portal	Ramaswamy et al., 2015	https://bbp.epfl.ch/nmc-portal; RRID: SCR_002994
NeuroMorpho.Org	Ascoli et al., 2007	http://www.NeuroMorpho.Org; RRID: SCR_002145
Janelia MouseLight project	Economo et al., 2016	http://ml-neuronbrowser.janelia.org; RRID: SCR_016669
Neuroscience Gateway	Sivagnanam et al., 2013	http://www.nsgportal.org; RRID: SCR_008915

CONTACT FOR REAGENT AND RESOURCE SHARING

For enquiries about any of the resources or models presented here, please contact the Lead Contact, R. Angus Silver, a.silver@ucl.ac.uk. For general queries about Open Source Brain, contact info@opesourcebrain.org.

METHOD DETAILS

OSB frontend: project and user management

The main OSB web interface (at http://www.opensourcebrain.org) is based on a heavily customized version of Redmine (http://www.redmine.org). This platform, developed in Ruby on Rails (https://rubyonrails.org), allows users to create accounts, make new projects and add other users to them, create user groups, and associate a version control repository (hosted on GitHub [https://github.com], BitBucket [https://bitbucket.org/], SourceForge [https://sourceforge.net], etc.) to each project. We have extended this framework with a new user interface, and added custom fields for projects with metadata relevant to the model, brain region, species, and simulators supported. Close integration with platforms hosting the version control repositories allows the files and change history to be deeply integrated into the OSB interface. Issues raised on GitHub, or forks of the repository are highlighted on the OSB page. Wikis describing the installation/usage of the models can be added directly on OSB, or content from README files in the associated repositories can be retrieved and displayed. An application programming interface (API) is provided to programmatically access information on all current projects and metadata (https://github.com/OpenSourceBrain/OSB_API). OSB project repositories are automatically searched for NeuroML files (in either XML or HDF5 format, see below) and these are presented to users for visualization through the browser.

OSB frontend: visualizing & simulating models

Visualization of models on OSB has been enabled through our contribution to the development of Geppetto (http://www.geppetto.org; Cantarelli et al., 2018), an open source modular framework built primarily in Java (server side) and JavaScript (browser side) that allows the content of files in various formats accessed on the server to be parsed, transformed, and visualized in the browser. The application was originally created in the OpenWorm project (Sarma et al., 2018; Szigeti et al., 2014), but it has developed into a modular platform with a number of parties contributing features (Cantarelli et al., 2018). The customized implementation of Geppetto for OSB provides extensive support for NeuroML models through the Java packages that we have developed (jNeuroML, see below). All that is required for interactive 3D visualization of these models in a web browser is WebGL (https://www.khronos.org/webgl), which is already present in most modern browsers. A number of JavaScript packages are used on the client side for 3D visualization

(WebGL; three.js), user interaction (React; D3) and plotting (Plotly). In addition to NeuroML, Geppetto can interpret and display SWC format (Cannon et al., 1998) as well as 3D objects in OBJ format (http://www.martinreddy.net/gfx/3d/OBJ.spec).

The OSB implementation of Geppetto provides a canvas for displaying and interacting with 3D objects (Figures 3, 5, 6, and 7D) as well as a number of visualization panels for displaying textual information, interaction elements such as buttons and menu items, and plots. Connectivity information extracted from networks can also be displayed in custom visualization panels on OSB (Figure 4). Interaction with the 3D objects and visualization panels can be solely through the graphical interface, but OSB also provides an integrated console for interactions with all these elements through JavaScript (enabled by clicking on the Console tab at the bottom of the 3D view (Figure 5A)). An example usage is changing the representation of neuronal morphologies to cylinders and using a minimum radius for dendrites of 1 μm (as in Figure 3C): *network.setGeometryType('cylinders',1)*.

NeuroML 2 & LEMS libraries

NeuroML version 2 (the version of the language supported by OSB) is built on a flexible low-level language, LEMS (Low Entropy Model Specification), which enables a wide range of physico-chemical processes to be defined (Cannon et al., 2014). To facilitate the use of NeuroML and LEMS models on OSB, we have developed a number of libraries for processing files in these standardized model description languages. These libraries have been developed in Java and Python, two of the most commonly used languages in computational neuroscience. A focus of the work has been on making these features available through easy to install packages. jNeuroML (https://github.com/NeuroML/jNeuroML) is a single package which gives access to all features which have been implemented in Java (Cannon et al., 2014). These include: natively parsing and simulating models specified in LEMS (including point neuron cell models and networks in NeuroML); converting NeuroML models to simulator-specific code (for currently supported simulators see Table S2); importing other structured formats to LEMS (particularly SBML (Hucka et al., 2003) models); validating NeuroML files, as well as performing basic tests for consistency; converting 3D models to SVG and PNG images.

libNeuroML (https://github.com/NeuralEnsemble/libNeuroML) is a Python package for reading, editing, and writing NeuroML files (Vella et al., 2014). pyNeuroML (https://github.com/NeuroML/pyNeuroML) is a Python package which builds on libNeuroML and bundles a copy of jNeuroML, allowing access to all of its functionality from Python scripts (most importantly converting NeuroML models to simulator code, running them, and reloading the results). Additionally, it has utility scripts for analyzing channel properties (*pynml-channelanalysis* for NeuroML channels; *pynml-modchananalysis* for NEURON channels) and *pynml-povray* for generating high resolution images and movies using POV-Ray (http://povray.org). Both jNeuroML and pyNeuroML can be used as command line applications (*jnml* and *pynml* respectively) or as libraries to give access to these features in other Java or Python applications (e.g., jNeuroML is bundled with neuroConstruct (Gleeson et al., 2007)). Both the Java and Python libraries can serialize NeuroML models as either XML or in binary HDF5 format (https://www.hdfgroup.org). The latter format has significant advantages in terms of file size (typically 10% of equivalent in XML) and speed of reading/writing, and can also be read from GitHub repositories for display in OSB.

Figure S1 illustrates how these libraries can be used during the process of converting and sharing models on OSB. All of these libraries are installed and configured for use with supported simulators in the OSB Docker image (Table S3; https://www.docker.com). Table S4 outlines the versions of the simulators, libraries, and programming languages used.

PyNN models

PyNN allows a single Python script to instantiate and run a network in either NEURON, NEST, Brian or on neuromorphic hardware (Schemmel et al., 2010); the only difference in the script is in the import of the package specific for that simulator (from *pyNN.neuron import **, *from pyNN.nest import **, etc.) (Davison et al., 2009). The NeuroML export from PyNN works similarly (*from pyNN.neuroml import **) and will export the cell parameters, connectivity, and inputs to valid NeuroML. An example of this is the increasingly widely used (Cain et al., 2016; Lee et al., 2017; Schmidt et al., 2018a, 2018b; Schwalger et al., 2017) cortical column model (Potjans and Diesmann, 2014) in Figures 4E–4H. The original conversion of this model to PyNN was extended with 3D distributions for cell populations without changing the overall network behavior, but making the structure of the network clearer when visualized on OSB.

Executing simulations on OSB

Having models specified in NeuroML format on OSB means these can easily be converted to a number of simulator specific formats for execution (Table S2). A user who is signed in on OSB and is viewing a NeuroML model can "persist" the 3D view (orange star on top icon bar, e.g., Figure 3A, Video S3), so that that version of the NeuroML file (along with the layout/visual properties/currently open visualization panels) is stored for opening again at any time (there will be a list of persisted models on the user's homepage when they log in). Simulations can be set up and run with a number of options including simulation duration, time step, what to record (e.g., all membrane potentials at cell somas), the numerical seed used for stochastic simulations, and which simulator to run it on. Currently supported simulators include NEURON, jNeuroML and NetPyNE (Dura-Bernal et al., 2019; http://netpyne.org). The latter is a Python package built on NEURON which greatly facilitates execution of robust, parallel network simulations in multiprocessor environments

(see below). The OSB website is hosted on an Amazon Web Services (AWS, https://aws.amazon.com) instance, on which the supported simulators are installed. Short simulations can quickly be run on a single processor on this AWS platform. Users are presented with a list of running and completed simulations (Figure 5A) and can plot recorded values, overlay them on cells for visualization of cell and network dynamics (Figures 6B and 6C), or create rasterplots or firing rate traces to see population level activity (Figure 7D).

An alternative option for running simulations is enabled through OSB integration with the Neuroscience Gateway (Sivagnanam et al., 2013) (NSG, Figure 1A). OSB generates the simulator scripts and submits these through the RESTful API of NSG (NSG-R, http://www.nsgportal.org/guide.html). These are then submitted to the Extreme Science and Engineering Discovery Environment (XSEDE) high performance computing resources for execution, where NSG has pre-configured multiple neuronal simulation packages. Users on OSB do not require any account on NSG or XSEDE, and completed simulations appear in the OSB interface in the same way, albeit with more of a delay than running on OSB's own servers. Because NSG provides access to parallel computing resources, OSB users can select NetPyNE as a target simulator, and opt to run network models across up to 256 processors, significantly speeding up the simulation time. This number can be increased in future as we gauge usage statistics/demand, to make greater use of the thousands of processors available via NSG. Scripts have also been developed which allow modelers to submit NeuroML and PyNN directly to NSG-R, bypassing the web interface (https://github.com/OpenSourceBrain/NSGPortalShowcase).

Data generated during simulations launched via OSB can be downloaded from the web interface, but can also be set to automatically be added to the user's Dropbox (https://www.dropbox.com) folder, to enable local analysis of the simulation data. This is enabled by generating an API key on the Dropbox site and adding this to the user's OSB account.

Existing tutorials for OSB are listed at http://www.opensourcebrain.org/tutorials. Documentation for those interested in developing and hosting tutorials and making use of the visualization and simulation features of OSB can be found at http://opensourcebrain.org/docs#Creating_Tutorials.

Interactions with neuroinformatics resources

The cell models used in the Blue Brain Project rat somatosensory microcircuitry network (Markram et al., 2015) have been made available in the original NEURON format on the Neocortical Microcircuit Collaboration Portal (NMCP) (Ramaswamy et al., 2015). We have developed scripts for automatically converting these cells to NeuroML and example models in this format have been made available on http://www.opensourcebrain.org/projects/blue-brain-project-showcase. We have also converted the ion channel models from the Channelpedia (Ranjan et al., 2011) database to NeuroML format in this OSB repository. Representative connectomes with point neurons used in recent studies of the Blue Brain Project microcircuit (Gal et al., 2017; Reimann et al., 2017) are available on the NMCP and a NeuroML HDF5 based version of this full connectome can be found on the OSB project. The full network (31346 cells, 7.6 million connections) cannot yet be displayed on OSB (though this is a target for future releases). However a scaled down version, with 5% of neurons in the original is available and can be visualized.

The Allen Cell Types Database (Hawrylycz et al., 2016) (http://celltypes.brain-map.org) contains neuronal reconstructions and electrophysiological recordings from multiple cells in mouse visual cortex (electrophysiological data samples shown in Figures S3A and S3C, see below). Computational models of these cells are also available on the website in both biophysically and morphologically detailed (implemented in NEURON simulator) and point neuron (Generalized Linear Integrate and Fire (GLIF) models in a custom Python simulator) formats. These have both been converted to NeuroML/LEMS formats on http://www.opensourcebrain.org/projects/alleninstituteneuroml. Each cell shown in Figure 3A has a unique complement of ionic conductance densities tuned to the original cell's electrophysiological recordings.

Neuronal reconstructions from http://neuromorpho.org/ (Ascoli et al., 2007) are available in standardized SWC format (Cannon et al., 1998) and these files, containing information on the 3D locations, radii, connectivity, and type of the points in the reconstruction, can be visualized directly on OSB by placing them into a GitHub repository (see http://www.opensourcebrain.org/projects/neuromorpho). These files can be converted to NeuroML, to build them into spiking neuron models with active conductances, by using the application at https://github.com/pgleeson/Cvapp-NeuroMorpho.org or by loading the SWC into neuroConstruct (Gleeson et al., 2007), editing the cells and exporting to NeuroML. The Janelia MouseLight project (Economo et al., 2016) provides SWC versions of their neuronal reconstructions, in addition to a proprietary JSON file format with extra metadata. The OSB project for this (http://www.opensourcebrain.org/projects/mouselightshowcase) provides scripts for converting the JSON files to NeuroML while retaining the metadata.

For both the NeuroMorpho.Org and MouseLight repositories, a small set of example cell files have been converted to NeuroML and added for visualization on OSB, and instructions/scripts are included for converting any other cells from those resources to NeuroML.

Testing and model validation

Model development requires systematic testing of the code base. In software engineering, it is standard practice to run automated tests whenever a change is made to the code and it is committed to a repository ("continuous integration"). To facilitate this on OSB we have developed the OSB Model Validation framework (OMV, https://github.com/OpenSourceBrain/osb-model-validation), which

allows test configuration files to be added to the repository for an OSB project. Expected behaviors (e.g., spike times, resting membrane potential), as well as consistency checks for parameters such as total membrane area and temperature are described in short Model Emergent Properties (mep) files. Then a number of OSB Model Test (omt) files are written for each of the simulator configurations (engines) which should produce that behavior, specifying the allowed tolerance. Simulator entries in the last 3 columns of Table S2 are generally associated with individual passing tests (one omt file per simulator) linked by producing the same behavior as described in a shared mep file.

OMV can be installed locally and run at command line (see Table S3) and is also used on the continuous integration (Duvall et al., 2007) service Travis-CI (https://travis-ci.org). Each time there is a commit to a GitHub repository for a model, OMV is launched during the test on Travis-CI, the appropriate simulators are installed and all of the OMV tests in that repository are set running. This ensures the full sequence of simulator installation, model execution, and validation is run on every change to the model code, quickly highlighting any deviations from expected behavior (or simulator compatibility issues).

Inhibition stabilized network models

The model shown in Figure 7B is a reimplementation of Sadeh et al. (2017) in PyNN (original spiking model developed in NEST). The networks consisted of 800 excitatory (E) and 200 inhibitory (I) neurons. E and I neurons had the same properties and were modeled using an exponential integrate-and-fire model (Brette and Gerstner, 2005), without adaptation. All neurons received a baseline input modeled as an independent homogeneous Poisson process with constant firing rate (9600 Hz). Recurrent connections were drawn from a binomial distribution, with average probability of 15% for $E \rightarrow \{E,I\}$ and 100% for $I \rightarrow \{E,I\}$ connections, reflecting the denser connectivity of inhibitory neurons as reported in the cortex (Hofer et al., 2011). The average connection strength of $E \rightarrow \{E,I\}$ and $I \rightarrow \{E,I\}$ connections, parameterized by the peak synaptic conductance, were set to 0.1 nS and 0.2 nS, and the reversal potentials for excitation and inhibition were 0 mV and -75 mV, respectively. The connection strength for background input was 0.1 nS. To detect ISN properties of the network, the baseline activity of the network was perturbed by changing the input to a fraction of inhibitory neurons. The perturbation was performed by *reducing* the baseline input to perturbed inhibitory neurons by 400 Hz (i.e., by $\sim$4%) and was repeated for multiple trials to obtain average firing rates before and after perturbation. For further details on the model, including detailed properties of neurons and connectivity, see Sadeh et al., 2017. Code is available in the *PyNN* subfolder of https://github.com/OpenSourceBrain/SadehEtAl2017-InhibitionStabilizedNetworks. The model was exported to NeuroML from PyNN to generate the 3D view on OSB.

In order to create more realistic conductance-based, ISN models, the exponential integrate-and-fire neuron model in Figure 7B was replaced with different neuron types in Figure 7C, while keeping other properties of the network connectivity and stimulation protocol similar. To this end, Hodgkin-Huxley type point neurons with voltage-gated membrane conductances from Pospischil et al. (2008) were scaled to match the firing behavior of layer 2/3 spiny (E, red) and aspiny (I, blue) neurons from the Allen Cell Types Database. Scripts were created to download electrophysiological data from mouse visual cortex neurons from the Allen Cell Types Database (subfolder *CellTypesDatabase/data* on https://github.com/OpenSourceBrain/AllenInstituteNeuroML). From membrane potential traces of cells receiving 1 s current pulse stimuli (examples shown in Figures S3A and S3C), information was extracted on input stimuli information, spike times, spike height, subthreshold steady states and these values were used to retune the model of Pospischil et al. (2008) (subfolder *CellTypesDatabase/tune* in above repository). This single compartment model featured a fast Na^{+}, delayed rectifier and M-type K^{+}, L-type Ca^{2+} and leak conductances. Conductance densities of active and leak channels, reversal potentials of ions (over a restricted, physiologically plausible range), cell capacitance and voltage dependence of the Na^{+} conductance were used as free parameters in the model tuning, for which the package Neurotune (https://github.com/NeuralEnsemble/neurotune) was used. Two cells were selected from Allen Cell Types Database for the E and I neurons in our network model. One cell from layer 2/3 which exhibited spiny dendrites and showed positive expression for the gene Slc17a6 (vesicular glutamate transporter) was chosen for the E cell (http://celltypes.brain-map.org/mouse/experiment/electrophysiology/477127614) and one with aspiny dendrites and positive expression for somatostatin was chosen for the I neuron (http://celltypes.brain-map.org/mouse/experiment/electrophysiology/476686112).

Figures S3A–S3D show the original electrophysiological data from these cells along with the equivalent behavior for the tuned cell models.

To address how ISN properties can be detected in more realistic scenarios involving detailed neurons equipped with nonlinear dendritic integration we built a hybrid model (Figure 7D). Here, the previous network (Figure 7C) was augmented by replacing 10 of the 800 E cells with the morphologically detailed cell model of Smith et al. (2013), while the axonal connections were omitted for these cells. Figure S3E shows the structure of the network and inputs/connections present, and Figure S4A shows connections generated onto the dendrites of one of the detailed cells. To detect the ISN signature in the detailed cells, the somata of two morphologically complex cells were voltage clamped at -80mV and 0 mV, revealing the E and I postsynaptic currents respectively (Figure 7D, inset). This process was repeated for 10 independent realizations of the model, to obtain a reliable estimate of the inputs. In order to generate multiple instances of a network model which had varying numbers of point neurons/detailed cells and configurable inputs, a script was created in Python (ISN.py, available in https://github.com/OpenSourceBrain/MultiscaleISN) which used libNeuroML to position the cells in 3D space, connect the populations and apply external spiking inputs. Both Figures 7C and 7D were generated from this script.

DATA AND SOFTWARE AVAILABILITY

All of the code for the OSB platform and the models presented here is open source. The Ruby on Rails based frontend for OSB is at https://github.com/OpenSourceBrain/redmine (GNU General Public License v2) and the Geppetto repositories are listed at https://github.com/openworm/org.geppetto (MIT License). The repositories for the majority of models in Figure 2A can be found at https://github.com/OpenSourceBrain (generally released under MIT License, with information on how to cite the models when reused in CITATION files). Direct links to individual OSB projects can be found in Table S1 and also at http://www.opensourcebrain.org/projects.

Neuron, Volume *103*

Supplemental Information

Open Source Brain: A Collaborative Resource for Visualizing, Analyzing, Simulating, and Developing Standardized Models of Neurons and Circuits

Padraig Gleeson, Matteo Cantarelli, Boris Marin, Adrian Quintana, Matt Earnshaw, Sadra Sadeh, Eugenio Piasini, Justas Birgiolas, Robert C. Cannon, N. Alex Cayco-Gajic, Sharon Crook, Andrew P. Davison, Salvador Dura-Bernal, András Ecker, Michael L. Hines, Giovanni Idili, Frederic Lanore, Stephen D. Larson, William W. Lytton, Amitava Majumdar, Robert A. McDougal, Subhashini Sivagnanam, Sergio Solinas, Rokas Stanislovas, Sacha J. van Albada, Werner van Geit, and R. Angus Silver

Reference	Physiological properties	Model implementation	Link
Allen Institute Cell Types DB (Hawrylycz et al., 2016)	Morphologically detailed and point neuron models based on electrophysiological recordings from visual cortex neurons	Multicompartmental and Generalized Linear Integrate and Fire (GLIF) neuron models	URL
Brunel (2000)	Spiking network illustrating balance between excitation and inhibition	Integrate and Fire (I&F) neurons, abstract network; implementations in PyNN, NeuroML and NEST	URL
Hay et al. (2011)	Layer 5 pyramidal cell model constrained by somatic and dendritic recordings	Detailed neuronal morphology, non uniform channel distributions	URL
Izhikevich (2003)	Spiking neuron model reproducing wide range of neuronal activity	2 variable point neuron model	URL
Markram et al. (2015)	Cell models from Neocortical Microcircuit of Blue Brain Project	Multicompartmental cell models of multiple cortical classes each with unique complement of active conductances	URL
Pospischil et al. (2008)	HH based model for different classes of cortical and thalamic neurons	Single compartment model with 5 ion channels reproducing a range of neuronal spiking behaviors	URL
Potjans and Diesmann (2014)	Microcircuit model of sensory cortex with 8 populations across 4 layers	Current based I&F neurons; implementations in PyNN, NeuroML and NEST	URL
Dura-Bernal et al. (2017)	Model of mouse primary motor cortex (M1)	Point neurons connected in columnar network based on realistic connection density distributions	URL
Sadeh et al. (2017)	Point neuron model of Inhibition Stabilized Network	Current based I&F neurons; implementations in native NEST and PyNN	URL
Smith et al. (2013)	Layer 2/3 cell model used to investigate dendritic spikes	Multicompartmental cell model with AMPA-R/NMDA-R mediated synaptic inputs	URL
Traub et al. (2005)	Single column network model containing 14 cell populations from cortex and thalamus	Semi-realistic neuronal morphologies; cell region specific distributions of active conductances; chemical & electrical synapses	URL
Cayco-Gajic et al. (2017)	Cerebellar granule cell layer network	I&F based model for granule cell and anatomically constrained connectivity	URL
Maex and De Schutter (1998)	Cerebellar granule cell layer network	Conductance based point neuron models for granule and Golgi cells	URL
Solinas et al. (2007)	Cerebellar Golgi cell model	Conductance based model with abstract morphology	URL
Vervaeke et al. (2010)	Electrically connected cerebellar Golgi cell network model	Detailed Golgi cell model; realistic cell density & gap junction connectivity properties	URL
Bezaire et al. (2016)	Full scale network model of CA1 region of hippocampus	Detailed and abstract models of 10 cell types, realistic firing properties and connectivity parameters	URL
Ferguson et al. (2013)	Parvalbumin-positive interneuron from CA1	Model is customized form of Izhikevich cell model	URL
Migliore et al. (2005)	Pyramidal cell from CA1 region of hippocampus	Multicompartmental cell model as used in a number of studies by Migliore and colleagues	URL
Pinsky and Rinzel (1994)	Simplified model of CA3 pyramidal cell	2 compartment model; can be simulated as a single set of ODEs	URL
Wang and Buzsáki (1996)	Hippocampal interneuronal network model exhibiting gamma oscillations	Conductance based point neuron model used in 100 cell all-to-all network model	URL
Migliore et al. (2014)	Large scale olfactory bulb network	600 unique multicompartmental mitral cells models along with simplified granule cells	URL
Boyle and Cohen (2008)	Model of body wall muscle from *C. elegans*	Point neuron model with 3 active conductances and internal Ca^{2+} buffer	URL
FitzHugh (1961)	Simplified form of Hodgkin Huxley model	2 variable cell model	URL
Hodgkin and Huxley (1952)	Classic investigation of the ionic basis of the action potential	HH model cell with current and voltage clamp inputs; model can be explored with interactive tutorial	URL
Prinz et al. (2004)	Pyloric network of the lobster stomatogastric ganglion system	3 conductance based point neuron models connected via analogue (graded) synapses	URL
NeuroMorpho.Org	Digitally reconstructed neurons across multiple species and brain regions	All cells from NeuroMorpho.Org can be downloaded in NeuroML2; representative examples on OSB	URL
Janelia MouseLight	Reconstructed neurons with axons projecting across the whole mouse brain	Cells downloaded from MouseLight website can be converted to NeuroML2; examples on OSB	URL

Supplementary Table 1. Models on Open Source Brain. Related to Figure 2A. A brief description of the properties of neuronal systems being modeled and the key model implementation features. Colors in first column correspond to the color code for brain regions in **Figure 2A**. Links are provided for accessing the model project pages at http://www.opensourcebrain.org.

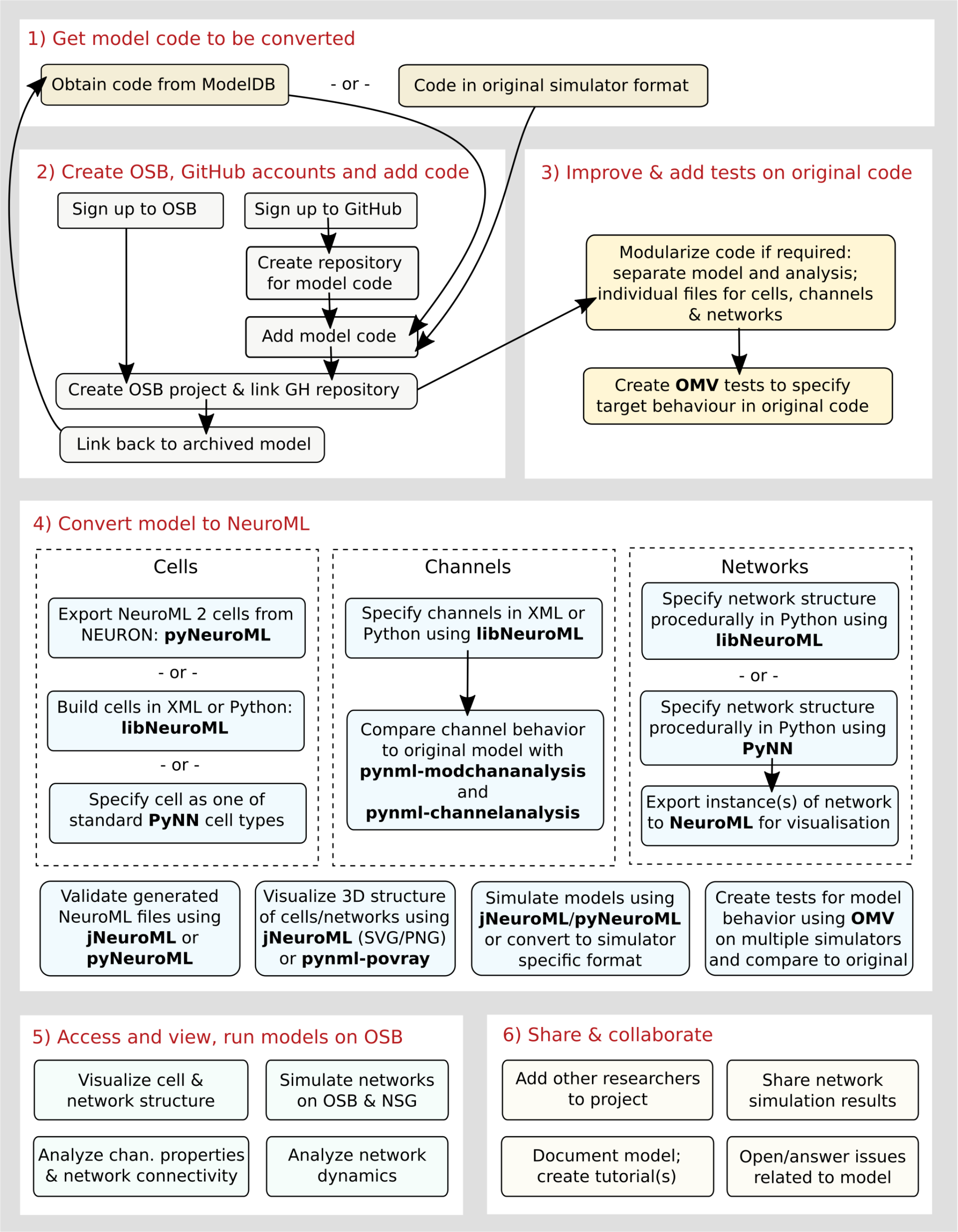

Supplementary Figure 1. Procedures and tools to convert models from native formats into NeuroML and PyNN on Open Source Brain. Related to Figure 1A. Outline of the steps required for converting a model from its native language into NeuroML/PyNN format in order to utilize the visualization and simulation functionality on Open Source Brain (OSB) and for utilizing automated validation and other collaborative development tools. 1) The model code in the original format is obtained from the model developer(s) or from repositories such as ModelDB (McDougal et al., 2017). 2) User accounts are created on OSB and on GitHub. A new repository is created on GitHub and linked to an OSB project for the model. 3) Optionally, the code should be cleaned up, further documented, and made more modular, with all changes recorded in the version control system on GitHub. OSB Model Validation framework (OMV) tests can be added to the scripts which record the behavior of cell(s) in the original model and which the converted model needs to replicate. 4) The numerous tools available to build, export (e.g. from NEURON), validate, visualize and simulate the NeuroML/PyNN version of the model elements on a user's local machine. As the structure of the model is replicated in NeuroML/PyNN, its properties can be tested against the behavior of the original model using OMV. 5) Once valid model elements are uploaded to GitHub, these can be visualized, analyzed and simulated on OSB. 6) Options available to make the model accessible, to get input from other users on the model and to share development responsibilities.

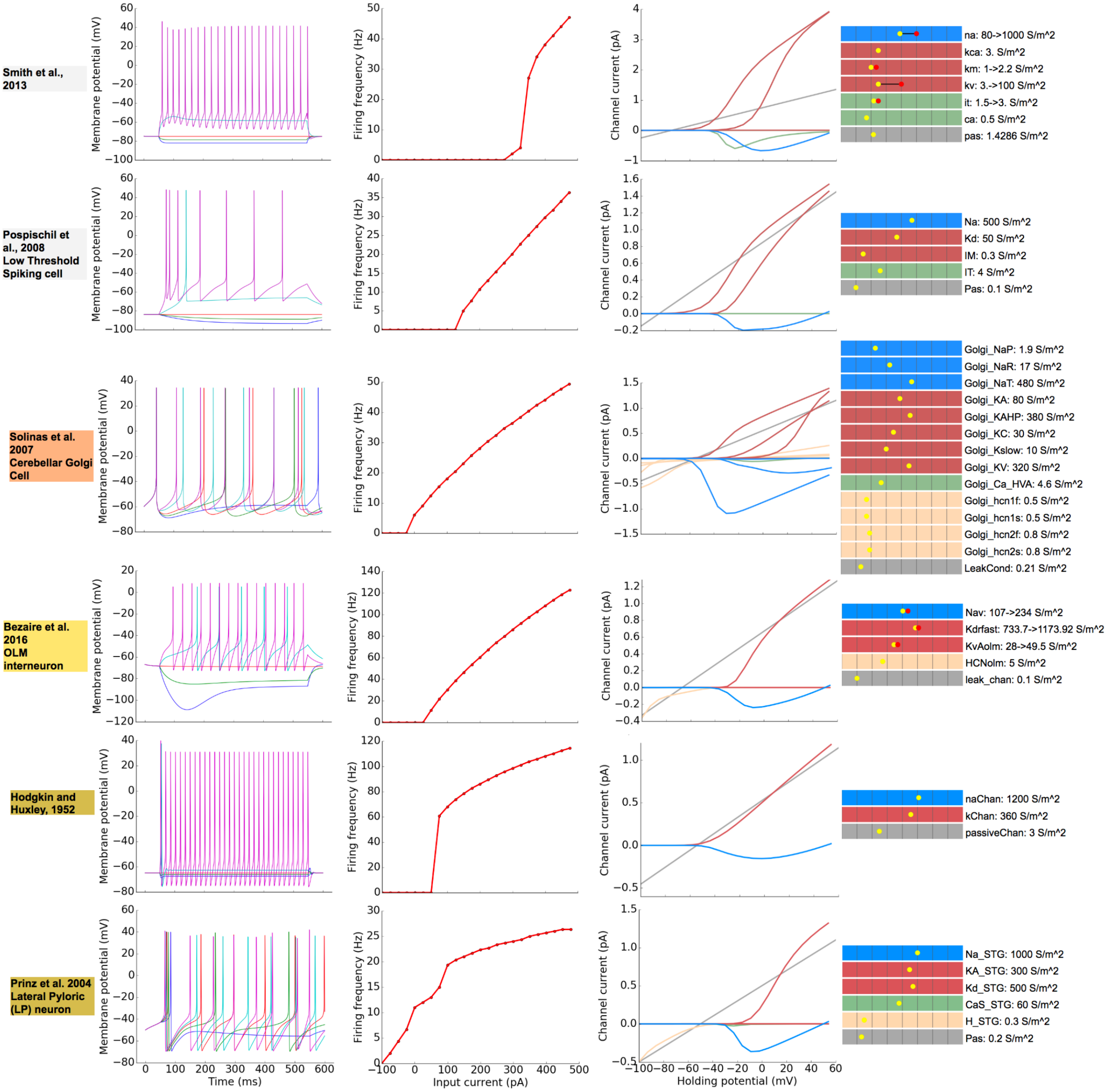

Supplementary Figure 2. Comparison of the properties of cells and membrane conductances on Open Source Brain. Related to Figure 2A. Once neuronal models have been converted to NeuroML format their physiological properties can be readily compared, using same scripts to simulate and analyze the properties of different models (e.g. in Python using libNeuroML (STAR Methods)). From left to right the columns show: the time course of the somatic membrane potential for 5 levels of somatic current injection (column 1); firing frequency as a function of current injected (column 2); currents through each conductance present in the soma when the cell is clamped at various holding potentials for the same conductance value (for the default minimal conductance of 10 pS in this case; column 3, colors correspond to colored scale in the next column); densities of the ionic conductances (column 4; logarithmic color scale shows densities of conductances grouped according to the ions transmitted: blue - Na^+, red - K^+, green - Ca^{2+}, light brown - non selective HCN channels, grey - passive conductances; yellow dot is value if conductance is uniform, yellow is minimum and red maximum if nonuniform).

Main publication	Valid NeuroML	PyNN	jNeuroML	jNeuroML to NEURON	Other tests
Allen Institute Cell Types DB (Hawrylycz et al. (2016)	✓		✓	✓	NEURON; NeuroML→ NetPyNE
Brunel (2000)	✓	Brian; NEURON; NEST; NeuroML	✓	✓	PyNEST; PyNN→ NeuroML
Hay et al. (2011)	✓		✓	✓	NeuroML→ NetPyNE
Izhikevich (2003)	✓	Brian; NEURON; NEST	✓	✓	Octave; PyNEURON
Markram et al. (2015)	✓		✓	✓	NEURON; NeuroML→ NetPyNE
Pospischil et al. (2008)	✓		✓	✓	NEURON; NeuroML→ Moose; NeuroML→ NetPyNE
Potjans and Diesmann (2014)	✓	NEST			NEST; PyNEST
Dura-Bernal et al. (2017)	✓		✓	✓	NetPyNE; NeuroML→ NetPyNE
Sadeh et al. (2017)		NEST			PyNEST
Smith et al. (2013)	✓		✓	✓	PyNEURON; NeuroML→ NetPyNE
Traub et al. (2005)	✓		✓	✓	neuroConstruct; NeuroML→ NetPyNE
Cayco-Gajic et al. (2017)			✓		
Maex and De Schutter (1998)	✓		✓	✓	NeuroML→ NetPyNE
Solinas et al. (2007)	✓		✓	✓	NEURON
Vervaeke et al. (2010)	✓			✓	
Bezaire et al. (2016)	✓		✓	✓	NEURON; NeuroML→ NetPyNE
Ferguson et al. (2013)		NeuroML→ PyNN→ NEURON	✓	✓	Brian; Brian2; NeuroML→ Brian2
Migliore et al. (2005)	✓		✓	✓	
Pinsky and Rinzel (1994)			✓		
Wang and Buzsaki (1996)			✓	✓	Brian; NEURON; NeuroML→ NetPyNE
Migliore et al. (2014)	✓			✓	PyNEURON; NeuroML→ NetPyNE
Boyle and Cohen (2008)	✓	NeuroML→ PyNN→ NEURON	✓	✓	Octave; NeuroML→ NetPyNE
FitzHugh (1961)	✓		✓	✓	NeuroML→ Brian; NeuroML→ Brian2
Hodgkin and Huxley (1952)	✓	NeuroML→ PyNN→ NEURON	✓	✓	NeuroML→ Brian2; NeuroML→ Moose; NeuroML→ NetPyNE
Prinz et al. (2004)	✓		✓	✓	NeuroML→ NetPyNE
NeuroMorpho.Org	✓				
Janelia MouseLight	✓				

Supplementary Table 2. Automated testing of models on Open Source Brain. Related to Figure 2A. The models shown in **Figure 2A** have been tested for the validity of their NeuroML implementations and against expected behavior across a number of simulators. Colors in the first column are those used in **Figure 2A**. Tests include: (column 2) validity of the NeuroML files in the repository; (column 3) behavior of the PyNN scripts on different simulator backends (e.g. Brunel (2000) can be run in Brian, NEURON and NEST and exported to NeuroML), or conversion of the NeuroML files to PyNN scripts and execution on a simulator (e.g. NEURON for Ferguson et al., 2013); (column 4) execution of the NeuroML models using jNeuroML's native simulator (point neuron models only); (column 5) execution of the NeuroML models in NEURON, translated using jNeuroML; (column 6) execution of original simulator code in the repository (e.g. NEURON, Brian or Octave scripts taken from ModelDB as used in original publications) or other simulator code (NetPyNE, Brian 2) produced by converting the NeuroML representation using jNeuroML. Note, some models are not fully valid NeuroML because they have custom LEMS components (e.g. Ferguson et al., 2013 uses a custom form of the Izhikevich model). The current testing status of these and all other projects on OSB can be found here: http://www.opensourcebrain.org/status.

Steps	Instructions
1) Install Docker	See https://www.docker.com; available for Windows, Mac OS or Linux.
2) Pull an image with all simulators and stable projects	`docker pull opensourcebrain/simulation:osb_models-v0.8.5`
3) Start a container with the image	`docker run -it opensourcebrain/simulation:osb_models-v0.8.5 /bin/bash`
4) Inside container, go to folder with models and run the tests using OMV	`cd coreprojects`<br>`omv all`

Supplementary Table 3. Obtaining all core OSB models and testing against supported simulators. Related to STAR Methods. A Docker image (preconfigured computational environment with files, libraries, etc.; https://www.docker.com) has been created containing all of the simulators currently supported by OSB, along with all of the models presented in **Figure 2** and **Supp. Table 1**. Once Docker is installed (1), the image can be pulled from the central Docker registry (2). A container using this image can be set running (3), and OMV (STAR Methods) used to run tests on each of the models (4), in all the simulators it supports. The commands in blue above need to be typed at the command line. Alternatively, the Kitematic application bundled with Docker can be used for steps 2) and 3), the image opensourcebrain/simulation:osb_models-v0.8.5 searched for and a new container created through the interface. The commands in (4) can then be entered into a command line terminal connected to this.

Software package	Version	Website
Brian	v1.4.4	http://briansimulator.org
Brian2	v2.2.1	http://briansimulator.org
MOOSE	v3.2-git	https://moose.ncbs.res.in/
NEST	v2.12.0	http://www.nest-simulator.org
NEURON	v7.4	https://www.neuron.yale.edu
NetPyNE	v0.7.9	http://www.netpyne.org
PyNN	v0.9.2	http://neuralensemble.org/PyNN
PyLEMS	v0.4.9.3	https://github.com/LEMS/pylems
jLEMS	v0.9.9.1	https://github.com/LEMS/jLEMS
jNeuroML	v0.8.4	https://github.com/NeuroML/jNeuroML
pyNeuroML	v0.3.15	https://github.com/NeuroML/pyNeuroML
Python	v2.7.9	https://www.python.org/
Java	v1.7.0	https://www.java.com
Octave	v3.8.2	https://www.gnu.org/software/octave

Supplementary Table 4. Libraries used in Docker container. Related to STAR Methods. The listed versions of the software packages shown for simulators (white rows), NeuroML/PyNN libraries (light grey) and software languages (blue) have been used to test the models presented here. These are also the versions included with the Docker container (**Supp. Table 3**).

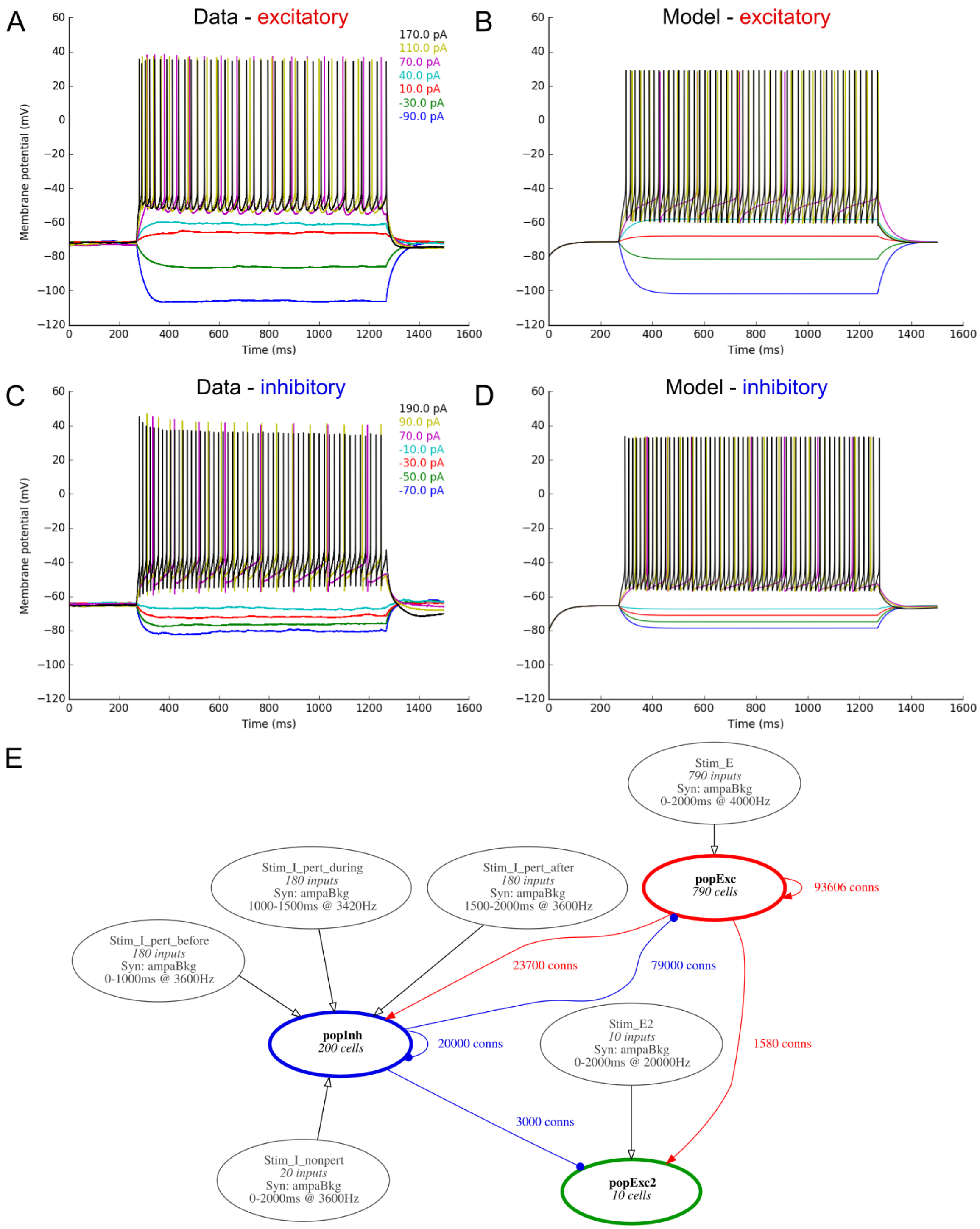

Supplementary Figure 3. Properties of single compartment neurons in hybrid model of inhibition stabilized network, together with connectivity. Related to Figure 7. (A-D) Plots of membrane potential traces from electrophysiological recordings of visual cortex neurons (A, C) and equivalent computational models (specified in NeuroML, executed in NEURON) generated to match the spiking behavior (B, D). Data in A and C taken from Allen Cell Types Database and are recordings of cells in Layer 2/3 of mouse visual (A: dataset 477127614, spiny cell; C: dataset 476686112, aspiny cell). Responses to 1 second current pulses of 7 different amplitudes are shown in each. B and D show the responses to the same inputs (as A and C respectively) of the single compartment computational models based on Pospischil et al., 2008 which have been tuned to reproduce the behavior of the experimental recordings (STAR Methods) and used in the network shown in **Figure 7C**. (E) Schematic diagram showing inputs to and connections between populations of excitatory point neurons (popExc), excitatory detailed neurons (popExc2) and inhibitory point neurons (popInh) for network shown in **Figure 7D**. 10% of the cells in popInh receive a steady input of 3600 Hz for the full 2 seconds, while 90% have a slightly lower input rate from 1-1.5 s. Voltage clamp inputs to 2 of 10 cells in popExc2 not shown for clarity.

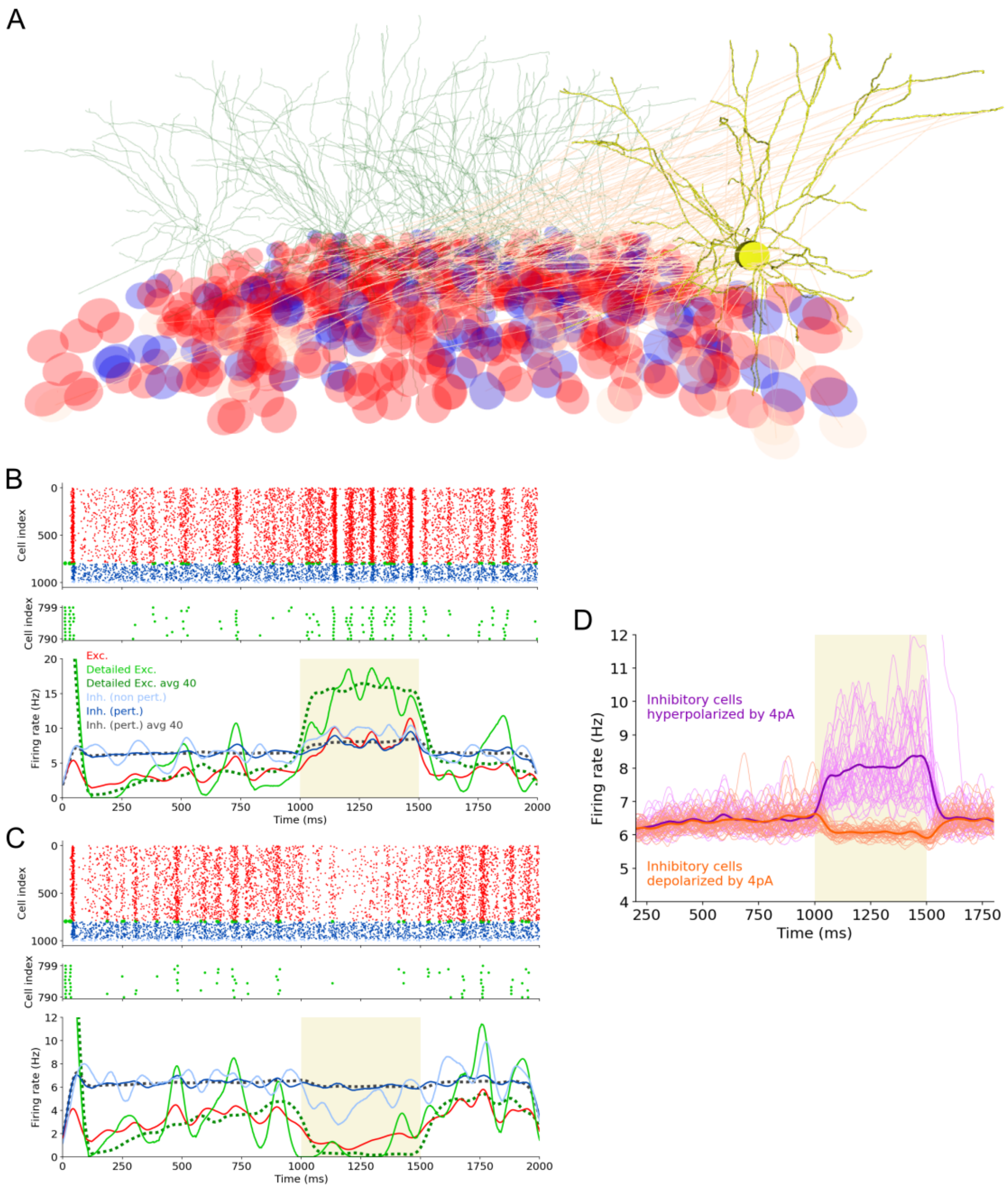

Supplementary Figure 4. Synaptic connectivity of hybrid model and response to the injection of hyperpolarizing and depolarizing currents into interneurons. Related to Figure 7D. (A) Screenshot from OSB of 3D network model (same model as shown in **Figure 7D**) showing connections from excitatory (red) and inhibitory (blue) point neurons onto the dendrites of a single detailed cell (color of selected cell set to yellow and dendrites given larger diameter; all other detailed cells in green). (B) Response of network under similar configuration to **Figure 7D/Supp. Figure 3E**, but with hyperpolarizing current of -4pA (1-1.5 s) applied to 90% of inhibitory cells, as opposed to a decrease in background firing rate. A similar increase in firing rates of all populations is seen (cells 790-799 are detailed cells, green points for these enlarged in upper rasterplot and shown in middle rasterplot; solid green line in lower plot is rate of firing of detailed cells; dashed green line is average of 40 simulations; other traces and calculation of firing rate same as **Figure 7B-C**). Hyperpolarizing current injection was designed to mimic optogenetic inactivation of interneurons with halorhodopsin (cf. Figure 5 of Kato et al., 2017). (C) Response of network to transient application of depolarizing current (4pA from 1-1.5 s). A decrease in firing of all populations is seen. Similar plots as for (B). (D) Plots of rate of firing in the 90% of stimulated interneurons when hyperpolarized (light purple lines: 40 individual simulations; thick dark purple line: average) and depolarized (light orange lines: 40 individual simulations; bold dark orange line: average). This shows the paradoxical effect of ISNs: when the interneurons receive a small positive current input they reduce their firing rates, when they receive a small negative current input they increase their firing rates.
